# Progress in Nanotechnology for Treating Ocular Surface Chemical Injuries: Reflecting on Advances in Ophthalmology

**DOI:** 10.1002/advs.202407340

**Published:** 2025-01-04

**Authors:** Qiaoran Qi, Dai Su, Shuqin Zhuang, Sunyuan Yao, Ludwig M. Heindl, Xianqun Fan, Ming Lin, Jin Li, Yan Pang

**Affiliations:** ^1^ Department of Ophthalmology Ninth People's Hospital Shanghai Jiao Tong University School of Medicine Shanghai 200011 China; ^2^ Shanghai Key Laboratory of Orbital Diseases and Ocular Oncology Center for Basic Medical Research and Innovation in Visual System Diseases Ministry of Education Shanghai 200011 China; ^3^ Department of Ophthalmology Faculty of Medicine and University Hospital Cologne University of Cologne 50937 Cologne Germany; ^4^ Center for Integrated Oncology (CIO) Aachen‐Bonn‐Cologne‐Duesseldorf Cologne Germany; ^5^ Shanghai Frontiers Science Center of Drug Target Identification and Delivery School of Pharmaceutical Sciences Shanghai Jiao Tong University Shanghai 200240 China

**Keywords:** drug delivery, nanotechnology, ocular surface chemical injury, therapy

## Abstract

Ocular surface chemical injuries often result in permanent visual impairment and necessitate complex, long‐term treatments. Immediate and extensive irrigation serves as the first‐line intervention, followed by various therapeutic protocols applied throughout different stages of the condition. To optimize outcomes, conventional regimens increasingly incorporate biological agents and surgical techniques. In recent years, nanotechnology has made significant strides, revolutionizing the management of ocular surface chemical injuries by enabling sustained drug release, enhancing treatment efficacy, and minimizing side effects. This review provides a comprehensive analysis of the etiology, epidemiology, classification, and conventional therapies for ocular chemical burns, with a special focus on nanotechnology‐based drug delivery systems in managing ocular surface chemical injuries. Twelve categories of nanocarrier platforms are examined, including liposomes, nanoemulsions, nanomicelles, nanowafers, nanostructured lipid carriers, nanoparticles, hydrogels, dendrimers, nanocomplexes, nanofibers, nanozymes, and nanocomposite materials, highlighting their advantages in targeted delivery, biocompatibility, and improved healing efficacy. Additionally, current challenges and limitations in the field are discussed and the future potential of nanotechnology in treating ocular diseases is explored. This review presents the most extensive examination of this topic to date, aiming to link recent advancements with broader therapeutic strategies.

## Graphical abstract

A review of progress in nanotechnology for treating ocular surface chemical injuries. This is the most detailed review on the topic, serving as a microcosm of advancements in the application of nanotechnology in ophthalmology. It analyzes 12 nanocarrier platforms, namely liposomes, nanoemulsions, nanomicelles, nanowafers, nanostructured lipid carriers, nanoparticles, hydrogels, dendrimers, nanocomplexes, nanofibers, nanozymes, and nanocomposite materials.

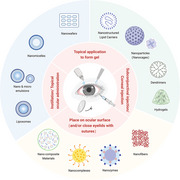

## 1 Introduction

Chemical injuries to the ocular surface are commonly encountered in ophthalmic emergencies, ranking as the second most prevalent injury after ocular foreign body incidents. These injuries are highly destructive, often leading to visual impairment, chronic pain, and prolonged treatment durations, making them some of the most challenging ocular surface conditions to manage. Alkali burns occur significantly more frequently than acid burns. Immediate and extensive irrigation is crucial minimizing the severity of the sequelae. Acute interventions aim to reduce the severity of the injury, promote re‐epithelialization, and control inflammation on the ocular surface. Chronic management focuses on suppressing inflammation, rehabilitating, and rebuilding the ocular surface. Additionally, treatment strategies aim to prevent neovascularization and stimulate ocular surface regeneration.

Conventional treatment primarily focuses on promoting healing, reducing inflammation, preventing infection, and lowering intraocular pressure (IOP). A variety of biological treatments and surgical procedures have been incorporated into standard treatment protocols to optimize outcomes. These include amniotic membrane transplants, platelet‐rich plasma, autologous cord serum and umbilical cord serum preparations, corneal limbal stem cell transplants, anti‐angiogenic agents, and corneal bandage lenses. In more severe instances, corneal disintegration may occur, requiring corneal grafting and enucleation in extreme instances, both of which significantly affect the individual's physical and psychological well‐being. Due to the limitation of traditional medications‐such as single‐action mechanisms, limited options, frequent dosing, side effects, and restricted efficacy‐nanotherapeutic delivery platforms have emerged as a solution to ensure sustained drug release. Over the past few years, nanotechnology‐based platforms have been proved to alleviate side effects dramatically with improved multifunctionality. At the same time, the development of nanotechnology for treating ocular surface chemical burns has advanced rapidly at an unprecedented pace. This review explores the application and advancements of nanotechnology in the medical management of ocular surface chemical burns. Additionally, various nano formulations comprising conventional medications and natural compounds are included.

Our review provides a detailed analysis of 12 categories of nanocarrier platforms used for treating ocular surface chemical injuries, including liposomes, nanoemulsions, nanomicelles, nanowafers (NWs), nanostructured lipid carriers (NLCs), nanoparticles (NPs), hydrogels, dendrimers, nanocomplexes, nanofibers, nanozymes, and nanocomposite materials. By examining how the application of nanotechnology enhances the efficacy of conventional drug treatments, this review stands as the most comprehensive examination of ocular surface chemical burn treatments to date. Furthermore, from the point regarding the development of nanotechnology in treating ocular surface chemical injuries, the platforms for treatment of other ocular related diseases will be inspired, serving as a microcosm in the field.

## 2 Ocular Surface Chemical Injuries

### 2.1 Etiology

The two most common types of ocular adnexa injuries are thermal and chemical, which differ in how the injury develops. Thermal burns stop causing tissue damage once the heat source is removed or the heat dissipates.^[^ ^1^ ^]^ However, immediate and effective treatment is crucial for chemical burns to the eyes. Chemical burns require prompt intervention to reverse damage and prevent further future injury to the ocular surface and deeper structures, as tissue damage can continue as long as the chemical remains in contact with the eye and surrounding adnexa. Chemical burns may result from exposure to common household substances such as ammonia, bleach, drain cleaner, laundry detergent, or oven cleaners.^[^ ^1^ ^]^ Industrial exposure to compounds like fertilizers, industrial acids, lye, lime, and cement can also cause injuries. Explosions from fireworks and other sources can inflict both thermal and chemical harm.^[^ ^2^ ^]^ In cases of blast injuries, special attention should be given to full‐thickness and penetrating wounds, as they may involve intraocular foreign bodies.

Alkali substances are more frequently responsible for chemical injuries to the eyes, with lime plaster being the most common cause.^[^ ^3^ ^]^ Almost two‐thirds of severe chemical burns to the eyes are thought to be caused by alkali, according to several reports in the literature.^[^ ^4^ ^]^ The pH is a crucial component in determining an alkali's toxicity, with a pH > 11.5 causing substantial corneal damage.^[^ ^5^ ^]^ It has long been known that alkali compounds penetrate more quickly than acids.^[^ ^3a^ ^]^ Ammonia has the quickest penetration rate (>3 min) among the various alkali chemicals that can result in serious eye burns. Following that are sodium hydroxide (NaOH) (3–5 min), calcium hydroxide (which varies due to its slow crystallization), and potassium hydroxide (>5 min).^[^ ^6^ ^]^ Therefore, most studies on ocular chemical injures use alkali burn animal models. Acid injuries generally cause less damage to the ocular surface than alkali injuries. Concentrated acids lead to denaturation and coagulation of proteins in the anterior stroma and corneal epithelium, resulting in corneal opacification. This coagulated protein barrier limits the acid's ability to penetrate deep into the corneal stroma.^[^ ^5b^ ^]^ Moreover, the anterior stroma and corneal epithelium have buffering capabilities, allowing for the neutralization of acidity.^[^ ^7^ ^]^ Sulfuric acid, the primary cause of acid‐related eye injuries, is less likely to cause severe ocular damage.

In this review, we concentrate on utilizing nanotechnology for treating chemical burns on the ocular surface. Alkali burn animal models are the preferred for ocular chemical burn research due to their severity. During the literature review on this topic, we found 103 studies of which 97% used a NaOH‐induced alkali burn model. In addition, silver nitrate cauterization‐induced acid burn model (1), absolute ethanol (3), 75% silver nitrate and 15% potassium nitrate (1), and vesicant‐exposed chemical burn models (1) are also used.

### 2.2 Epidemiology

Although ocular burns occur frequently, but the exact incidence rate remains unknown.^[^ ^8^ ^]^ Previous studies have found that 10.7 to 34.7% of all chemical burn injuries occur on the ocular surface.^[^ ^9^ ^]^ According to epidemiological data, chemical burns to the eyes account for ≈11.5–22.1% of all ocular injuries,^[^ ^3^, ^10^ ^]^ including unintentional incidents and intentional assaults.^[^ ^4^, ^11^ ^]^ Chemical burns are the second most common ocular injury, following foreign object trauma.^[^ ^1^ ^]^ These injuries occur more often in men than in women, although women tend to suffer them at a younger age.^[^ ^10^, ^12^ ^]^ Children aged 1 to 2 are affected twice as frequently as adults.^[^ ^10a^ ^]^ In one database, alkali injuries were more common, accounting for approximately 53.6% of cases, compared to 46.4% for acid injuries.^[^ ^10a^ ^]^ Some sources report that the ratio of acid to alkali burns is ≈1:2.^[^ ^5a^ ^]^ Acid burns are most frequently caused by sulfuric acid, while alkali burns are typically associated with substances containing ammonia.^[^ ^8^ ^]^

### 2.3 Classification

In clinical practice, there are various classification schemes for chemical burns to the ocular surface. The two most commonly employed classifications are the Roper–Hall classification and the classification proposed by Dua et al.^[^ ^13^ ^]^ It is very useful to have a standard classification to guide clinical treatment and prognosis. The categorization method initially proposed by Ballen et al.^[^ ^14^ ^]^ has been further developed through the refinement introduced by the Roper–Hall classification, as detailed in **Table** **1**.^[^ ^13a^ ^]^ The severity of this system is assessed by evaluating the degree of limbal conjunctival ischemia and corneal damage. Limbal conjunctival ischemia has traditionally served as a crucial marker for corneal limbal stem cell injury, with its level of ischemia bearing significant importance for both immediate and prolonged recovery of the eye's health.^[^ ^15^ ^]^

**Table 1:** Assessing the severity of chemical burns on the ocular surface using the Roper–Hall (Ballen) classification.

Cornea Injury and Opacity	Limbus			Prognosis	Grade
Cornea	Iris	Limbal ischemia (±) ^a)^	Ischemic Range		
Damage of corneal epithelial	–	–	–	Good	I
Corneal haze	Visible iris features	+	<1/3	Good	II
Loss of total epithelial, Haze in stroma	Obscured iris details	+	1/3–1/2	Guarded	III
Opaque cornea	Pupil & iris obscured	+	>1/2	Poor	IV

^a)^ + represents cases with limbal ischemia; ‐ represents no limbal ischemia.

To enhance prognostic stratification, especially for severe injuries, the classification system proposed by Dua et al. (**Table** **2**) was devised.^[^ ^13b^ ^]^ It was based on 67 chemical‐burn cases' (35 retrospective and 32 prospective). The method accounts for limbal injury (clock hours) and conjunctival injury (%).

**Table 2:** The classification system for chemical injuries to the ocular surface as introduced by Dua et al.

Grade	Prognosis	Clinical observations^a)^	Involvement of conjunctiva [%]	Analog scale [%]
I)	Very good	0	0	0/0
II)	Good	⩽3	⩽30	0.1–3/1–29.9
III)	Good	>3–6	>30–50	3.1–6/31–50
IV)	Good to guarded	>6–9	>50–75	6.1–9/51–75
V)	Guarded to poor	>9 < 12	>75 < 100	9.1–11.9/75.1–99.9
VI)	Very poor	12 (Total limbus involved)	100 (Total conjunctival involved)	12/100

^a)^ Showing the degree of limbal engagement in clock‐hour increments, range from 0 to 12.

The two most commonly used classifications, Roper‐Hall and Dua et al., have different strengths and weaknesses (**Table** **3**).^[^ ^13^ ^]^ The Roper‐Hall classification stands as a pragmatic, direct, and precise prognostic categorization scheme extensively utilized in clinical settings. However, it notably overlooks the evaluation of conjunctival impairment, a pivotal factor in forecasting the progression of symblepharon and corneal deterioration (see Table 3). Additionally, it has a limitation in that all injuries exhibiting over 50% limbal ischemia are categorized as grade IV. Despite this shortcoming, advancements in treatments, such as limbal stem cells transplantation, have positively impacted the prognosis for severe chemical injuries to the eye. It is also noteworthy that some therapeutic approaches were poorly delineated during the development of the Roper‐Hall classification. Furthermore, the classification technique proposed by Dua et al.^[^ ^13b^ ^]^ presents certain limitation; some healthcare practitioners find it challenging to apply, and it overlooks considerations regarding corneal involvement and limbal vascularity (see Table 3).^[^ ^13b^ ^]^

**Table 3:** Comparison of the Dua et al. and Roper‐Hall classifications.

Classification	Corneal involvement (±) ^a)^	Limbal involvement		Conjunctival involvement
		Injury	Vascularity	
Dua et al.	–	+	–	+
Roper‐Hall	+	–	+	–

^a)^ +, represent involved; −, represent not involved.

The Dua et al. and Roper‐Hall classifications employ distinct approaches to assess limbal stem cell damage. While the Dua et al. classification requires limbal fluorescein staining, the classification of Roper‐Hall relies on the presence of perilimbal ischemia. Dua et al. note that even mild limbal ischemia can indicate substantial stem cell loss, particularly in cases of total loss of limbal epithelium. The classification introduced by Dua et al. warrants attention due to its failure to account for corneal involvement.

Although the existing classification systems adequately evaluate the surface area of the injury, a notable limitation is their failure to illustrate the depth of the injury. Utilizing more advanced imaging techniques, such as anterior segment optical coherence tomography (AS‐OCT), in the current clinical setting may help to partially document the extent of involvement.^[^ ^16^ ^]^ Therefore, it is recommended to employ more visual techniques to assess and grade ocular surface chemical burns.

In a clinical setting, it is essential to document the initial extent of conjunctival, corneal, and limbal involvement at the time of injury, as well as to monitor and record any changes observed during the patient's treatment process.^[^ ^12^ ^]^ The prognosis may be graded to provide the patient with an overall impression.

### 2.4 Principle and Process of Conventional Treatment

According to previous research, conventional treatments of ocular surface chemical burns mainly include drug and surgical therapies. The concepts of early therapy of ocular chemical burns include removal of chemicals, adequate irrigation, suppression of excessive inflammatory response, promotion of epithelial regeneration, reduction of corneal lysis, and control of IOP. The principles of treatments in the late stage are to release lid adhesions, reconstruct the eyelid and ocular surface, and restore visual function and appearance.^[^ ^17^ ^]^

**Figure 1:**
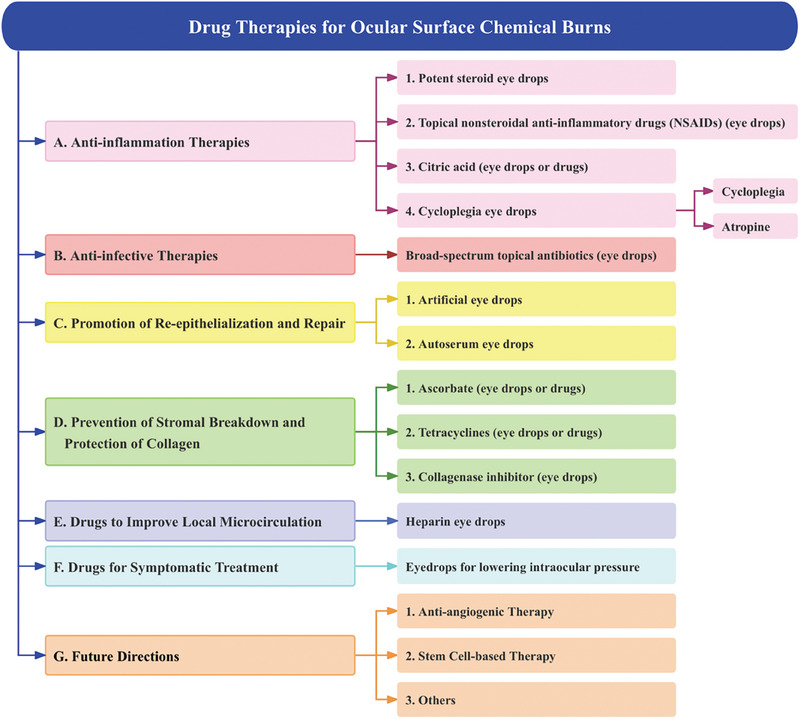
Drug therapy procedures for ocular surface chemical burns.

Anti‐inflammation therapies, anti‐infective therapies, promotion of re‐epithelialization and repair, prevention of stromal breakdown and protection of collagen, medications for improving local microcirculation and to relieve symptomatic are common drug therapies for ocular surface chemical burns (**Figure** **1**).^[^ ^17^, ^18^ ^]^ Recently, researchers pay more attention to the treatment of anti‐angiogenic and stem cell‐based therapy aiming to find more effective methods for ocular surface chemical burns, especially for the protection of the cornea.

**Figure 2:**
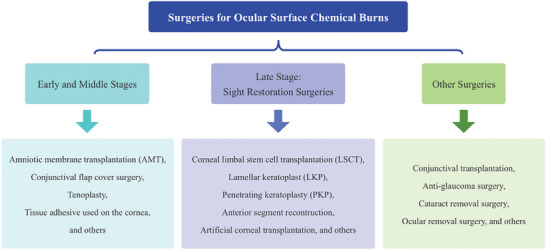
Surgical procedures for ocular surface chemical burns.

Approaches for managing ocular surface chemical burns differ depending on the stage of the condition (**Figure** **2**).^[^ ^17^ ^]^ When a substantial corneal epithelial defect is present, and the ulcer's depth increases with imminent or actual corneal perforation, surgical intervention is recommended. Early surgery can be necessary when corneal damage is caused by incomplete eyelid closure due to deformity. Surgery should be performed after managing and stabilizing ocular surface inflammation in patients whose wounds have healed. Amniotic membrane transplantation, conjunctival flap cover surgery, tenoplasty, and the use of tissue adhesive on the cornea are surgical options during the early and middle stages.^[^ ^17^, ^18^ ^]^ Procedures for late‐stage sight reconstruction encompass artificial corneal transplantation, anterior segment reconstruction, penetrating keratoplasty (PKP), lamellar keratoplasty (LKP), and corneal limbal stem cell transplantation.^[^ ^17^ ^]^ There are still other surgeries for chemical burn treatment, such as conjunctival transplantation, anti‐glaucoma surgery, cataract‐removal surgery, and ocular removal surgery.^[^ ^17^ ^]^

Treating ocular surface chemical burns often requires frequent medication, which inevitably leads to adverse effects such as keratitis, impaired vision, and corneal toxicity. To mitigate these side effects and enhance drug efficacy, emerging drug delivery technologies are gaining attention. Nano‐based drug delivery technologies are at the forefront of these advancements, working to extend the duration of drug efficacy on the ocular surface with reduced side effects. Although traditional treatment methods are crucial for managing ocular surface chemical burns, challenges remain in protecting the cornea and preventing vision loss and related impacts on physical and mental health. Nanotherapy, as an emerging field, shows promise in improving treatment outcomes and reducing adverse effects, particularly for ocular surface chemical burns. Despite this, there is currently very few comprehensive reviews summarizing the application of nanotherapeutics in this area. This review aims to provide an overview of the latest research on the use of nanotherapeutics for treating ocular surface chemical burns.

## 3 Nano Therapies for Addressing Ocular Surface Chemical Burns

**Figure 3:**
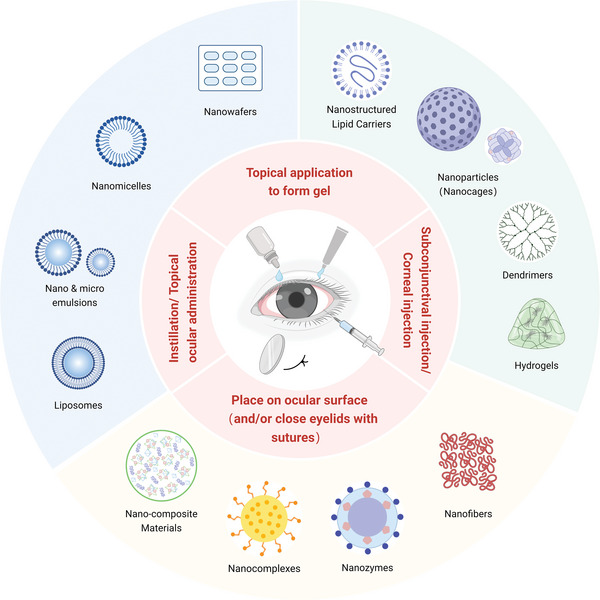
Exemplary schematic diagram illustrating nanocarrier systems employed in the management of ocular surface chemical burns (Created with BioRender.com).

Optimal drug delivery to the eye remains a challenge despite extensive research into treatment for ocular surface chemical burns. The anatomy and structure of the eye allow for the application of topical medications to facilitate localized distribution of drugs.^[^ ^19^ ^]^ This approach may reduce the likelihood of adverse reactions while also lengthening precorneal residence time and enhancing drug absorption in the eye.^[^ ^19^ ^]^ There is a diverse classification of nanocarriers, and nano‐drug delivery has become a promising aspect of the pharmaceutical sciences. The increased use of nanocarrier drug‐delivery systems stems from technological advancements and a deeper understanding of diseases at the cellular and molecular levels.^[^ ^19^ ^]^ Nanocarrier systems typically range in size from 1 to 1000 nm and are often hydrophobic.^[^ ^19^, ^20^ ^]^ In addition to their interaction with the ocular mucosa, nanocarrier systems possess a unique ability to enhance medication permeability across the cornea and conjunctiva.^[^ ^19^, ^21^ ^]^ Corneal and conjunctival cells readily uptake nanocarriers smaller than 200 nm.^[^ ^22^ ^]^ Numerous nanocarrier platforms, including liposomes, nanoemulsions, nanomicelles, NWs, NLC, NPs, hydrogels, dendrimers, nanocomplexes, nanofibers, nanozymes, and nano‐composite materials (**Figure** **3**), are currently being explored for the management of ocular surface chemical burns. These nanocarrier systems, extensively discussed in the literature, are poised to enhance the efficacy of existing medications and propose novel therapeutic avenues. Using PubMed and Google Scholar as the primary research tools, this section provides a thorough analysis of nanocarrier systems for the treatment of chemical burns at the ocular surface, covering in vitro, ex vivo, and in vivo investigations (**Table** **4**).

**Table 4:** Studies on nanocarrier application for ocular surface chemical burns.

Nanocarrier type	Loaded Agents/Materials	Objectives	Conclusion	Effects	Disease model	Therapy method	Reference, Year
Liposomes	Loaded Agents						
Liposomes	Rapamycin (RAPA)	To investigate the functional mechanism, the inhibitory effect of RAPA, and comparison with RAPA dissolved in bean oil to the rat alkali burn model's corneal neovascularization (CNV).	RAPA liposomes can better restrict the growth of CNV than RAPA solution in bean oil and may also reduce vascular endothelial growth factor (VEGF) expression through inhibition of the transcription factor hypoxia‐inducible factor 1‐alpha.	·Anti‐CNV	Corneal alkali burn (NaOH)/ Wistar rats	Instillation	[23], 2009
Liposomes	Verbascoside	To confirm the effectiveness of liposomal eyedrops containing verbascoside in treating corneal wounds caused by alkali exposure and to evaluate the corneal retention capacity of liposomes through manipulation of their surface charge.	Topical administration of verbascoside‐based liposomal eyedrops accelerates corneal epithelial wound healing, demonstrating a reduction in oxidative damage and apoptotic cells, while indicating a need for improved delivery efficiency for quicker therapeutic effects.	·Anti‐oxidation ·Promote corneal epithelial healing	Corneal alkali burn (NaOH)/ Adult hares	Instillation	[24], 2014
Liposomes	Usnic acid	To evaluate the safety and potential therapeutic efficacy of a gelatin membrane containing usnic acid/liposomes (UALs) for the healing of corneal wounds.	After the application of UALs through topical ocular means, it was observed that they were both safe and efficient in stimulating tissue restoration following chemical burns. This resulted in a notable decrease in injury occurrence and a heightened expression of VEGF and transforming growth factor beta 1 (TGF‐β1) in rabbit eyes. UAL treatment presents a promising pharmaceutical option for accelerating corneal healing, indicating its potential use in ophthalmic applications.	·Promote corneal healing	Corneal chemical burn (absolute ethanol), and perform epithelium removal/ Female rabbits	Topical ocular administration	[25], 2020
Liposomes	XAV939	Liposomes loaded with XAV939 (XAV939 NPs), an inhibitor of the Wnt/‐catenin pathway, were developed to show both anti‐inflammatory and antiangiogenic effects.	XAV939 NPs demonstrated superior efficacy over free XAV939 in mitigating corneal ulcers following corneal alkali burns, managing inflammation, and curtailing the progression of CNV. Administered via liposomal delivery, XAV939 NPs exhibited prolonged ocular surface retention and increased bioavailability compared to XAV939 alone, thereby enhancing therapeutic effectiveness while ensuring reliable systemic biosafety.	·Anti‐inflammation ·Anti‐CNV	Corneal alkali burn (NaOH)/ Female C57BL/6 mice	Instillation	[26], 2021
Liposomes	FK506	To enhance the efficacy of FK506 in treating CNV and inflammation by utilizing cationic FK506 liposomes, offering a novel approach to improving ocular drug‐delivery system bioavailability	The incorporation of liposomes alongside FK506 exhibited superior efficacy compared to FK506 alone, suggesting enhanced therapeutic outcomes in treating CNV and inflammation, thereby highlighting the advantages of utilizing liposomal formulations in ocular drug‐delivery systems.	·Anti‐inflammation ·Anti‐CNV ·Promote corneal epithelial healing	Corneal alkali burn (NaOH)/ C57BL/6 mice	Instillation	[27], 2021
Liposomes	Pirfenidone (PFD)	The physical and biological properties of a topical formulation containing PFD encapsulated in liposomes were assessed using a mouse model of corneal alkali injury.	The anti‐inflammatory and anti‐fibrotic properties of PFD encapsulated within liposomes were significantly enhanced compared to PFD dissolved in an aqueous solution when treating alkali burns affecting the cornea. This improvement was evidenced by reductions in corneal edema, haze dimensions and intensity, corneal thickness, and infiltration of corneal inflammation. These findings suggest promising prospects for effectively addressing corneal haze.	·Anti‐fibrotic ·Anti‐inflammation	Corneal alkali burn (NaOH)/ Male C57BL/6 mice	Instillation	[28], 2022
Liposomes	Ferrostatin‐1 (Fer‐1)	In vivo experiments were conducted to demonstrate the efficacy and safety of utilizing Fer‐1‐loaded liposomes (Fer‐1‐NPs) to enhance the bioavailability of Fer‐1 in the treatment of corneal alkali burns.	Fer‐1‐NPs offer a promising avenue for safe and effective treatment of corneal alkali burn by targeting ferroptosis, suppressing inflammation and neovascularization, with demonstrated efficacy comparable to dexamethasone (DEX) and no observed adverse effects, thus highlighting their potential as a novel therapeutic strategy.	·Anti‐ferroptosis ·Anti‐inflammation ·Anti‐CNV	Corneal alkali burn (NaOH)/ Male C57BL/6 mice	Instillation	[29], 2022
Nanoemulsions	Loaded Agents						
Nanoemulsions	Naringenin (NAR)	The objective is to enhance the formulation of NAR microemulsions (NAR‐MEs) by employing central composite design response surface methodology utilizing the design of experiments technique. This aims to assess their efficacy in vitro and in vivo as a topical ophthalmic delivery system for treating CNV in a mouse model, and to explore ocular pharmacokinetics in rabbits.	The enhanced drug release illustrated by the optimized NAR‐ME formulation resulted in elevated absorption within the cornea, ultimately boosting bioavailability. This, in turn, effectively suppressed CNV by reducing the expression of corneal VEGF and matrix metalloproteinase‐14 (MMP‐14). As a result, it emerges as a hopeful and secure method for delivering treatments targeting CNV.	·Anti‐CNV	Corneal alkali burn (NaOH)/ Male BALB/c mice	Instillation	[30], 2022
Nanoemulsions	Ro5‐3335	Examining the effectiveness of an eNano‐Ro5‐infused topical nanoemulsion formula, aimed at impeding the activity of runt‐related transcription factor 1 (RUNX1), in managing ocular neovascularization triggered by alkali burns.	The nanoemulsion within the cornea could effectively release RUNX1 and reach effective concentrations. After an alkali burn, the application of eNano‐Ro5 topically could considerably lessen CNV and speed up corneal wound healing.	·Anti‐CNV	Corneal alkali burn (NaOH)/ Male and female C57BL/6J mice	Topical ocular administration	[31], 2022
Nanoemulsions	Isoliquiritigenin (ISL)	To evaluate the anti‐vascular effects of a newly developed nanoemulsion containing ISL (ISL‐loaded nanoemulsion, ISL‐NE) in corneal alkali burn.	The novel ISL‐NE can help ISL overcome the limitation of poor aqueous solubility and insufficient bioavailability. ISL‐NE implementation demonstrates a promising, safe, and effective strategy in addressing CNV, inhibiting the expression of VEGF‐A and MMP‐2.	·Anti‐CNV	Corneal alkali burn (NaOH)/ Male BALB/c mice	Instillation	[32], 2022
Nanoemulsions	Sunitinib (STB)	To develop a topical STB‐ME eye drop for inhibiting CNV.	STB‐ME, exhibiting remarkable stability and bioavailability, efficiently inhibited CNV through topical application, indicating its promise as a platform for ocular drug delivery in CNV treatment.	·Anti‐CNV	Corneal alkali burn (NaOH)/ BALB/c mice	Instillation	[33], 2023
Nanomicelles	Loaded Agents						
Nanomicelles	Axitinib	To evaluate the efficacy of axitinib, administered through the amphiphilic copolymer MPEG‐PCL, in inhibiting angiogenesis, as compared to DEX employed as a reference standard, in the management of alkali‐induced CNV in rat specimens.	Micelles loaded with axitinib displayed improved dispersibility of the compound in water and showed anti‐angiogenic effects without causing evident tissue toxicity.	·Anti‐CNV	Corneal alkali burn (NaOH)/ Sprague Dawley (SD) rats	Instillation	[34], 2019
Nanomicelles	Pterostilbene (Pt)	To evaluate the therapeutic effectiveness of rebaudioside A (RA) micelles and their potential to enhance the therapeutic outcomes of Pt.	Self‐assembled ultrasmall nanomicelles based on RA offer a solution to the challenge posed by poorly water‐soluble drugs like Pt, showcasing significant promise in enhancing their ocular bioavailability.	·Antioxidation ·Anti‐inflammation	Corneal alkali burn (NaOH)/ C57BL/6 mice	Instillation	[35], 2020
Nanomicelles	Cabozantinib (Cabo)	To create micelles of cationic polypeptides (Cabo‐NPs) loaded with the lipophilic compound Cabo, aiming for prolonged release and the inhibition of CNV.	Cationic polypeptide micelles encapsulating Cabo offer a potential alternative for safe and efficient treatment of CNV, demonstrating enhanced therapeutic effects in preclinical models.	·Anti‐CNV	Corneal alkali burn (NaOH)/ Male C57BL/6 mice	Instillation	[36], 2020
Nanomicelles	DEX	To develop a therapeutic nanoplatform with bioadhesive properties using boric acid chemistry for extended trans‐corneal delivery of corticosteroids, with the objective of enhancing the efficacy of choroidal neovascularization treatment.	Nano‐formulations incorporating boric acid chemistry exhibit the capability to bolster increased drug volumes and prolong drug residency within the ocular environment. Consequently, this fosters enhanced transcorneal transportation of corticosteroids, thereby manifesting heightened therapeutic efficacy in addressing corneal alkali burn‐induced choroidal neovascularization.	·Anti‐CNV ·Anti‐inflammation	Corneal alkali burn (NaOH)/ SD rats	Instillation	[37], 2021
Nanomicelles	Rebamipide (RBM)	Investigating the capacity of dipotassium glycyrrhizinate (DG), a salt derived from glycyrrhizin dipotassium, as a nanocarrier to encapsulate RBM, with the objective of formulating an ophthalmic solution (DG‐RBM) aimed at enhancing therapeutic efficacy in the treatment of corneal alkali burns.	A novel ocular topical formulation, based on Glycyrrhizin‐derived DG‐RBM, shows significant potential for the treatment of corneal alkali burns through its targeted modulation of HMGB1 signaling pathways.	·Anti‐inflammation ·Anti‐ROS	Corneal alkali burn (NaOH)/ Male C57BL/6 mice	Instillation	[38], 2021
Nanomicelles	A cannabinoid receptor 2 (CB2r) agonist (TA‐A001)	To investigate the efficacy of a CB2r agonist, TA‐A001, encapsulated in SmartCelle™ micelles for treating moderate corneal alkali burns, comparing it with the corticosteroid prednisolone.	Micelle‐encapsulated TA‐A001 shows potential for modulating inflammation and promoting corneal wound healing, presenting a promising alternative to corticosteroids.	·Promote corneal healing ·Anti‐inflammation	Corneal alkali burn (NaOH)/ Female BALB/c mice	Instillation	[39], 2023
Nanomicelles	Empagliflozin (EMP)	To design a novel EMP@glycymicelle ophthalmic solution using EMP and glycyrrhizin to treat corneal alkali burns.	The EMP@glycymicelle ophthalmic solution offers a promising and effective nano‐delivery system for treating corneal alkali burns, significantly improving drug solubility and therapeutic outcomes.	·Antioxidant activities ·Anti‐CNV ·Anti‐inflammation ·Promote corneal healing	Corneal alkali burn (NaOH)/ Male C57BL/6 mice	Instillation	[40], 2023
Nanowafers (NWs)	Loaded Agents						
NWs	Axitinib	To establish the efficacy of NW containing axitinib in managing CNV within a mouse model experiencing ocular burns.	In mouse models of corneal alkali burn, the axitinib NW demonstrated superior efficacy in inhibiting CNV and augmenting suppression of proinflammatory and proangiogenic factors, surpassing the effectiveness of treatment with topical eye drops, even when administered less frequently.	·Anti‐CNV ·Anti‐inflammation	Corneal alkali burn (NaOH)/ Female C57BL/c mice	Place on the cornea each day	[41], 2015
NWs	DEX	Assessing the efficacy of a controlled‐release DEX delivery apparatus in a simulation model mimicking ocular burn and desiccation stress (OB+DS).	DEX‐NW, which administers DEX in a sustained‐release manner, when administered once daily, demonstrates efficacy similar to DEX drops administered four times daily. The efficiency of this method becomes evident in maintaining the clarity of the cornea and reducing the production of MMPs and inflammatory cytokines in the corneal tissues of mice suffering from the OB+DS model.	·Anti‐CNV ·Anti‐fibrosis ·Promote corneal healing	Corneal alkali burn (NaOH)+ subject to desiccating stress (DS) to create an OB+DS model/ Female C57BL/6J mice	Place in direct contact with cornea	[42], 2016
NWs	/	To elucidate the production process of a polymer wafer based on dextran sulfate (DS‐wafer) designed to impede fibrosis and inflammation, and to showcase its efficacy in two murine models of corneal damage: corneal abrasion and alkali‐induced ocular burn.	As a drug‐free polymer, DS‐wafer can improve therapeutic effectiveness for reducing CNV and corneal scarring as well as for accelerating wound healing.	·Anti‐CNV ·Anti‐fibrosis	Corneal alkali burn (NaOH)/ Female C57BL/6 mice	Place on the cornea each day	[43], 2021
Nanostructured Lipid Carriers (NLCs)	Loaded Agents						
NLCs	RAPA	Design a new drug administration platform to treat corneal alkali burn injuries by using NLCs to load RAPA.	Using NLCs to deliver RAPA to treat corneal injury could be regarded as a promising method to realize antifibrotic and angiogenic effect to protect the eyesight of patients.	·Anti‐fibrosis ·Alleviating corneal haze	Corneal alkali burn (NaOH)/ Male BALB/c mice	Instillation	[44], 2019
NLCs	Itraconazole (ITR)	Investigating the potential of NLCs loaded with ITR as an innovative approach for managing diabetic retinopathy (DR) and CNV.	NLCs in CS‐ITR showcased increased efficacy as transporters for delivering medication to the eyes, resulting in enhanced preservation and infiltration into ocular structures. Their potential as a promising therapeutic approach is indicated for addressing DR and the proliferation of blood vessels in the cornea.	·Anti‐CNV	Corneal alkali burn (NaOH)/ Swiss albino rats	Topical ocular administration	[45], 2019
NLCs	Dasatinib	Formulating dasatinib into a NLC with the purpose of targeting CNV in mice subjected to alkali burns.	Dasa‐NLC demonstrated significant potential in managing CNV.	·Anti‐CNV	Corneal alkali burn (NaOH)/ Male BALB/c mice	Instillation	[46], 2021
NLCs	Sorafenib (SRB)	To enhance drug solubility, bioavailability, and sustained release, to create SRB‐loaded NLCs for topical ocular delivery, focusing on effectively targeting CNV.	SRB‐NLCs exhibit efficient inhibition of CNV advancement by enhancing drug delivery, sustaining release, and improving ocular bioavailability, presenting a hopeful therapeutic strategy for managing CNV.	·Anti‐CNV	Corneal alkali burn (NaOH)/ Male BALB/c mice	Instillation	[47], 2022
Nanoparticles (NPs)	Loaded Agents						
Peptide and Protein NPs	Loaded Agents						
Protein NPs	pCMV.Flt23K	To find out if plasmid albumin NPs can be used to establish long‐term intraceptors expression and if they can prevent or reverse mechanical‐chemically induced murine ocular neovascularization.	NPs composed of albumin have the capacity to sustain the expression of intraceptors over extended durations, which proves beneficial for the suppression of injury‐triggered ocular neovascularization while exhibiting non‐toxicity towards the cornea.	·Anti‐CNV	Corneal alkali burn (NaOH)+remove corneal and limbal epithelia with knife/ BALB/c mice	Inject into the corneas	[48], 2007
Peptide NPs	Binding‐induced fibrillogenesis peptide (BIF peptide)	Presented is an innovative therapeutic strategy employing a peptidic network antibody (pepnetibody), which targets a single receptor, aimed at the efficient management of CNV. This approach entails the formation of a fibrous network that obstructs various receptors on the membranes of endothelial cells concurrently.	The effectiveness of a novel therapeutic strategy using pepnetibody targeting a single molecular pathway is showcased in this research. Employing precise nanoscale architectural arrangements, this approach achieves significant efficacy in addressing CNV, thereby introducing a promising direction for managing diseases associated with angiogenesis.	·Anti‐CNV	Corneal alkali burn (NaOH)/ Male New Zealand strain albino rabbits	Subconjunctival (SCJ) injection	[49], 2021
Peptide NPs	gp91ds‐tat peptide (gp91)	To investigate the efficacy of the innovative nanomedicine, GNP‐gp91, which encapsulates the gp91 peptide, in preventing CNV.	GNP‐gp91, a nanomedicine containing gp91 peptide encapsulated in gelatin NPs, demonstrates significant efficacy in inhibiting CNV, suggesting its potential as a clinically viable treatment option for ocular neovascularization.	·Anti‐CNV ·Anti‐ROS	Corneal alkali burn (NaOH)/ Male C57BL/6J mice	Instillation	[50], 2023
Protein NPs	Bevacizumab (Beva)	To develop a novel strategy for generating protein‐metal NPs by mixing metal ions with protein drugs, exemplified by Beva coordinated with zinc ions to form Beva‐Zn^2+^ NPs.	Beva‐NPs effectively inhibited CNV and overcame the limitations of protein drugs and protein‐based NPs, offering broad potential applications for various disease treatments.	·Anti‐CNV	Corneal alkali burn (NaOH)/ Male SD rats	Instillation	[51], 2024
Lipid NPs	Loaded Agents						
Lipid NPs	Angiopoietin‐like protein 2 (ANGPTL2)	To explore the application of a lipid NP‐delivered single‐stranded short hairpin RNA interference molecule modified with proline targeting ANGPTL2 (Li‐pshRNA ANGPTL2) for addressing CNV in a murine model of chemical‐induced injury.	The localized administration of lipid NPs containing Li‐pshRNA targeting ANGPTL2 demonstrates significant efficacy in suppressing CNV and decreasing ANGPTL2 levels within the cornea. This indicates its promise as a feasible therapeutic approach for managing CNV and addressing inflammatory conditions in the eye.	·Anti‐CNV ·Anti‐inflammation	Corneal alkali burn (NaOH)/ C57BL/6 mice	Instillation	[52], 2016
Lipid NPs	small interfering (siRNA)	Innovating a lipid NP responsive to reactive oxygen species (ROS) to deliver siRNA for corneal injury treatment aims to enhance RNA interference for potential CNV therapy in mice with alkali burn‐induced injuries.	Utilizing ROS‐responsive lipid NPs for enhancing RNAi in CNV refinement shows potential as a clinical treatment to mitigate the formation of CNV.	·Anti‐CNV ·Anti‐ROS	Corneal alkali burn (NaOH)/ Female BALB/c mice	SCJ injection	[53], 2022
Lipoprotein NPs (LPs)	Loaded Agents						
LPs	A plasmid expressing the soluble very low‐density lipoprotein receptor (VLDLR)	To ascertain if the suppression of Wnt signal within ocular tissues is due to the existence of the VLDLN extracellular domain(VLN).	A fresh therapeutic tactic for managing pathological ocular angiogenesis, as well as other vascular conditions influenced by Wnt signaling, could involve the NP‐based production of VLN.	·Anti‐CNV	Corneal alkali burn (NaOH)/ SD rats	Topical ocular administration	[54], 2015
LPs	microRNAs	To establish whether HDL NPs are efficient carriers for the transport of miRNAs to the cornea and to demonstrate the therapeutic efficacy of HDL NPs in wound healing and/or ocular inflammatory disorders.	Cells and tissues can internalize high‐density lipoprotein NPs complexed with microRNAs (miR‐HDL NPs), preserving their biological function. This encompasses the reduction of inflammation observed in a murine model subjected to corneal alkali burns and the augmentation of corneal wound re‐epithelialization in diabetic mice.	·Anti‐inflammation ·Promote corneal healing	Corneal alkali burn (NaOH)/ Male C57BL/6 mice	Topical ocular administration	[55], 2020
Glycopolymeric NPs (Chitosan (CS))	Loaded Agents						
CS	Bovine lactoferrin and SurR9‐C84A (SR9) proteins	An ex vivo model of bovine corneal alkali burn was used to explore the potential advantages of upcoming ophthalmic formulations and nanoengineered natural proteins, focusing on their absorption and protective properties.	CS and poly(lactic‐co‐glycolic acid) (PLGA) nano‐formulations encapsulating bLf or SR9 demonstrated efficacy in treating alkali‐induced ocular damage, exhibiting non‐irritating properties and excellent permeability.	·Anti‐inflammation ·Promote corneal healing	Corneal alkali burn (NaOH)/ An ex vivo model of bovine cornea	Topical ocular administration	[56], 2015
CS	Survivin protein (SR9) and trichostatin‐A (TSA)	Using a rat model of alkali‐induced eye injury, this research sought to explore the regenerative abilities of TSA, which inhibits histone deacetylase, along with the dominant‐negative survivin protein (SR9) in a living organism.	To enhance the healing process of alkali‐burned corneas and effectively deliver TSA and SR9 to the site of ocular burns, a promising approach involves using ultra‐small CS NPs conjugated with alpha‐smooth muscle actin (α‐SMA) antibodies.	·Anti‐inflammation ·Promote corneal healing	Corneal alkali burn (NaOH)/ Wistar rats	Topical ocular administration	[57], 2017
CS	CS and thiolated‐CS (TCS)	To investigate the potential of CS and TCS NPs and solutions in preventing fibroblast proliferation, their differentiation into myofibroblasts, neovascularization, extracellular matrix deposition, and the expression of pro‐fibrotic cytokines, this study aims to propose new anti‐fibrotic and anti‐angiogenic therapies for treating corneal chemical injuries.	CS and its mucoadhesive derivative, TCS, represent promising therapeutic options for combating corneal haze and fibrosis by inhibiting fibroblast proliferation, myofibroblast differentiation, neovascularization, and pro‐fibrotic cytokine expression, thus potentially preventing vision impairment post corneal injuries.	·Anti‐fibrosis ·Anti‐CNV	Corneal alkali burn (NaOH)/ Male BALB/c mice	Instillation	[58], 2018
CS	Bromfenac Sodium (BF)	Explore the capability of NPs comprising BF and CS (BF/CS‐NPs) in inhibiting ocular neovascularization.	BF/CS‐NPs possess the capability to hinder the progression of CNV during various stages after a corneal alkali burn, while also decreasing the expression levels of VEGF and cyclooxygenase‐2 in the impacted ocular tissues.	·Anti‐CNV	Corneal alkali burn (NaOH)/ New Zealand white rabbits	Instillation	[59], 2022
CS	Insulin and siVEGF	The study introduces a novel method for the treatment of corneal alkali burns using NPs composed of trimethyl CS (TMC) encapsulated within liposomes, incorporating insulin and siVEGF. Furthermore, it explores the potential mechanisms by which these NPs, designated as siVEGF‐TIL (siVEGF‐TMC‐INS‐liposome), operate to manage corneal alkali burns.	Therapeutic efficacy of siVEGF‐TIL in managing corneal injury post alkali burns and exploring its mode of action. siVEGF‐TIL treatment is not just secure and harmless but also demonstrates remarkable compatibility with biology and sustained release properties, opening fresh possibilities in mending corneal injury.	·Anti‐ ferroptosis ·Anti‐ROS ·Anti‐inflammation ·Anti‐CNV ·Promote corneal healing	Corneal alkali burn (NaOH)/ Male SD rats	Instillation	[60], 2023
Synthetic Polymers	Loaded Agents						
*PLGA*:							
PLGA	pSEC.shRNA.‐VEGF‐A plasmids	To assess the effectiveness of pSEC.shRNA, a plasmid containing a unit for expressing small hairpin RNA, delivered using PLGA NPs that encapsulate VEGF‐A, in achieving sustained suppression of ocular neovascularization in mouse models.	An effective, safe, and durable gene therapy approach for reducing murine neovascularization, induced by mechanical alkali damage in BALB/c mice, involves utilizing pSEC.shRNA‐loaded PLGA NPs targeting VEGF‐A.	·Anti‐CNV	Corneal alkali burn (NaOH)+ scrape the corneal and limbal epithelium by knife/ Female BALB/c mice	Corneal intrastromal injection	[61], 2012
PLGA	PFD	To analyze how PFD NPs affect corneal scarring and re‐epithelialization, two significant clinical issues following alkali burn.	PFD NPs present a promising therapeutic avenue not only for corneal chemical burns but also for various other fibrotic conditions affecting the cornea.	·Anti‐fibrosis ·Promote corneal healing	Corneal alkali burn (NaOH)/ SD rats	Instillation	[62], 2013
PLGA	Lingzhi	NPs composed of PLGA were developed to improve the efficiency and uptake rates of lingzhi in treating the rabbit corneal alkali burn model, ensuring sustained drug release.	LZH‐NPs administered topically could potentially improve healing in cases of corneal injuries and decrease inflammation in a rabbit model of corneal alkali burns. These NPs have the capability to alleviate ocular surface conditions triggered by oxidative stress.	·Anti‐ROS ·Anti‐inflammation	Corneal alkali burn (NaOH)/ New Zealand albino rabbits	Instillation	[63], 2021
PLGA	Minocycline (MINO)	To create an innovative drug‐delivery approach utilizing NPs of nano‐hydroxyapatite/PLGA (nHAP/PLGA) loaded with MINO. The goal is to efficiently suppress CNV and improve treatment effectiveness.	The MINO@PLGA nanocomplex demonstrated effective inhibition of CNV by modulating inflammatory pathways, showed minimal cytotoxicity, and displayed high in vivo biosafety. It offers a potential alternative for treating ocular diseases linked to CNV.	·Anti‐CNV ·Anti‐inflammation	Corneal alkali burn (NaOH)/ Male SD rats	Instillation	[64], 2024
PLGA	DEX sodium phosphate (DSP)	To investigate an innovative therapeutic strategy for halting the advancement of corneal damage induced by nitrogen mustard (NM) exposure, using a solitary SCJ administration of DSP‐loaded PLGA‐DSP‐NPs.	DSP loaded in PLGA NP carriers provides sustained release of the drug, demonstrating efficacy in treating corneal injuries caused by NM exposure.	·Anti‐CNV ·Anti‐inflammation	Corneal chemical injury (NM)/ Male SD rats	SCJ injection	[65], 2024
*Poly (ethylene glycol) (PEG)*:							
PEG	Apatinib	Investigating the application of NPs loaded with apatinib to inhibit angiogenesis mediated by VEGF and to suppress CNV in experimental models of alkali burn to the cornea.	Utilizing HSA‐PEG NPs as a carrier for delivering apatinib presents a potential strategy for preventing and treating CNV.	·Anti‐CNV	Corneal alkali burn (75% silver nitrate and 25% potassium nitrate)/ SD rats	SCJ injection	[66], 2017
PEG	DEX	Assessing the effectiveness of a new nano‐prodrug (DEX‐PEG‐APRPG, DPA) in targeting treatment for CNV.	The DPA nano‐prodrug provides targeted, efficient, and safe therapy for CNV, offering significant advantages over traditional treatments.	·Anti‐CNV ·Anti‐inflammation	Corneal alkali burn (NaOH)/ Male C57BL/6 mice	Instillation	[67], 2024
*Polyethylenimine (PEI)*:							
PEI	TGF‐β_1_ siRNA	The goal of encapsulating siRNA that targets TGF‐β1 using PEI is to suppress the activity of the TGF‐β1 gene. This gene plays a crucial role in fibrosis development linked to inflammation following corneal burns.	NPs containing siRNA targeting TGF‐β1 exhibit notable effectiveness in averting angiogenesis and reducing corneal haze after chemical injuries.	·Anti‐fibrosis ·Anti‐inflammation ·Anti‐CNV	Corneal alkali burn (NaOH)/ Male BALB/c mice	Topical ocular administration	[68], 2018
Liquid Crystalline NPs (LCNPs)	Loaded Agents						
LCNPs	PFD	To develop a liquid crystal NP system incorporating PFD (PFD‐LCNPs) and evaluate its efficacy for efficient delivery of ocular drugs, followed by assessment in a rabbit model of ocular chemical burns.	The PFD‐LCNPs could demonstrate promise in facilitating efficient delivery of drugs to the eye.	·Anti‐inflammation ·Promote corneal healing	Corneal chemical burn (absolute ethanol)/ Female New Zealand rabbits	Instillation	[69], 2019

Inorganic NPs	Loaded Agents							
Inorganic NPs	Silica NPs (SiNPs)	/	To determine whether topical SiNPs can prevent chemical burns from causing ocular neovascularization.	SiNPs are a useful method for preventing CNV after chemical burn in an experimental paradigm.	·Anti‐CNV	Corneal acid burn (Silver nitrate cauterization)/ Male Wistar albino rats	Instillation	[70], 2015
Inorganic NPs	Mesoporous SiNPs (MSNs)	Beva	Exploring the feasibility of employing MSNs as a framework for delivering nano‐drugs to improve antiangiogenic treatment.	Beva that is MSN‐encapsulated is a possible antiangiogenic treatment substitute.	·Anti‐CNV	Corneal alkali burn (NaOH)/ Male C57BL/6J mice	SCJ injection	[71], 2019
Inorganic NPs	Cerium oxide NPs (CeNPs)	/	Investigating the application of CeNPs to manage CNV, a common consequence of alkali burns to the cornea.	CeNPs significantly inhibit oxidative stress and inflammation associated CNV, suggesting a promising approach for long‐term treatment of ophthalmic diseases.	·Anti‐ROS ·Anti‐inflammation ·Anti‐CNV	Corneal alkali burn (NaOH)/ SD rats	Instillation	[72], 2019
Inorganic NPs	Tetramethyl orthosilicate	siRNA targeting the Fidgetin‐like 2 (FL2) gene	To assess the effectiveness of FL2‐NPsi, an siRNA targeting the FL2 gene encapsulated in NPs, in the treatment of alkaline chemical injury to the cornea in rat models.	FL2‐NPsi treatment markedly improves the healing of corneal wounds in a rat model of chemical injury, indicating its promise as an innovative therapeutic strategy for corneal injuries.	·Promote corneal healing	Corneal alkali burn (NaOH)/ Male SD rats	Instillation	[73], 2021
Inorganic NPs	Sperminated hyaluronan (sH)‐functionalized nanoceria (Ce‐sH NPs)	Insulin‐like factor 1 (IGF‐1)	Developing Ce‐sH NPs for holistic treatment of corneal alkali injuries, targeting oxidative stress, inflammation, apoptosis, angiogenesis, and fibrosis.	The Ce‐sH NPs showed promising efficacy in alleviating corneal alkali burns by addressing multiple pathological mechanisms.	·Anti‐fibrosis ·Anti‐CNV ·Anti‐inflammation ·Anti‐ROS ·Anti‐apoptosis	Corneal alkali burn (NaOH)/ SD rats	Instillation	[74], 2023
*Nanocages*:								
Nanocages	Ceria nanocages	Acetylcholine chloride (ACh) and SB431542	To design ceria nanocages coated with poly(l‐histidine) to facilitate targeted therapeutic administration for the management of chemical eye injuries.	Poly(l‐histidine)‐coated ceria nanocages significantly improve drug delivery and therapeutic outcomes for chemical eye injuries by enhancing drug bioavailability and controlled release.	·Promote corneal healing ·Antioxidant ·Anti‐inflammation ·Anti‐angiogenic ·Anti‐apoptotic ·Anti‐fibrosis	Corneal alkali burn (NaOH)/ Rats	Instillation	[75], 2023
Hydrogels	Materials	Loaded Agents						
Thermosensitive Hydrogel	Materials	Loaded Agents						
Nanocomposite Thermosensitive Hydrogel	Doxycycline temperature‐sensitive hydrogel (DTSH)	Beva	Investigating the synergistic therapeutic potential of a temperature‐sensitive hydrogel incorporating topical doxycycline (DTSH) and Beva on both CNV and corneal wound healing, including elucidating the associated mechanisms.	A potential advancement in treating CNV is demonstrated through the concurrent application of a DTSH and Beva topically. This combination shows promise in not only inhibiting CNV but also enhancing corneal wound healing, offering a promising alternative therapy for CNV.	·Anti‐CNV ·Anti‐inflammation ·Promote corneal healing	Corneal alkali burn (NaOH)/ Female SD rats	Topical application	[76], 2011
Cellular‐Enhanced Hydrogels	A thermo‐gelling injectable carboxymethyl‐hexanoyl CS (CHC) nanogel	Induced pluripotent stem cells (iPSCs)	Investigating the capability of human corneal keratocyte‐derived iPSCs, in conjunction with a thermo‐gelling injectable nanogel made from CHC, for innovative treatment of corneal regeneration and repair.	The utilization of human corneal keratocytes' iPSCs, in conjunction with a CHC nanogel delivery system, presents a promising strategy to augment the healing and regeneration of corneal wounds.	·Promote corneal healing	Corneal alkali burn (NaOH)+ scrape the corneal and limbal epithelium by knife/ Rats	Cover the injured eyes and close the eyelids with suture	[77], 2012
Polysaccharide‐Based / Cellular‐Enhanced Thermosensitive Hydrogel	A polysaccharide hydrogel	Mesenchymal stem cells (MSCs)	Investigating the possible therapeutic benefits of combining a polysaccharide hydrogel derived from Hardy Orchid with MSCs to enhance the healing process of corneal alkali injuries in rodent models.	The use of polysaccharide hydrogel derived from Hardy Orchid, in combination with MSCs, demonstrates a notable improvement in the recovery from alkali burns on the cornea. This approach presents a new and promising method for the treatment of injuries to the ocular surface.	·Promote corneal healing ·Anti‐CNV ·Anti‐inflammation	Corneal alkali burn (NaOH)/ Female SD rats	Topical ocular administration	[78], 2015
CS‐Based Thermosensitive Hydrogel	Thermosensitive CS‐based hydrogels	Ferulic acid (FA)	Exploring the effects of FA on corneal epithelial cell responses under oxidative stress and evaluating a temperature‐sensitive CS hydrogel containing FA for corneal wound healing potential.	Alkali burn treatment for the cornea could potentially utilize hydrogel loaded with FA.	·Promote corneal healing ·Anti‐ROS	Corneal alkali burn (NaOH)/ New Zealand albino rabbits	Instillation	[79], 2016
Nanocomposite Thermosensitive Hydrogel	A thermosensitive poloxamer hydrogel	Suramin (SUR), Beva	To assess the efficacy of a thermosensitive hydrogel that merges SUR with Beva to extend antiangiogenic effects in corneal therapies.	The poloxamer hydrogel with thermosensitivity, incorporating SUR and Beva, provides an extended and enhanced anti‐angiogenic impact against CNV, thereby diminishing the necessity for frequent drug dosing.	·Anti‐CNV	Corneal alkali burn (NaOH)/ SD rats	SCJ injection	[80], 2016
CS‐Based Thermosensitive Hydrogel	A thermosensitive CS–gelatin hydrogel	Recombinant human stromal cell‐derived factor‐1 alpha (rhSDF‐1 alpha)	Investigating the application of a gelatin–CS hydrogel sensitive to temperature, loaded with stromal cell‐derived factor‐1 alpha (SDF‐1 alpha), to enhance regeneration of the corneal epithelium through the attraction of MSCs.	The application of a hydrogel containing rhSDF‐1 alpha enhances regeneration of the corneal epithelium, indicating potential clinical utility in treating severe ocular surface diseases and repairing the cornea.	·Promote corneal healing	Corneal alkali burn (NaOH)+ scrape the corneal epithelium by knife/ Male SD rats	Cover the injured eyes and close the eyelids for 10 mins to from a gel	[81], 2017
PLGA‐PEG‐PLGA‐Based Thermosensitive Hydrogel	PLGA‐PEG‐PLGA thermosensitive hydrogel	Metformin (MET) and levofloxacin hydrochloride (LFH)	Designing a coherent co‐delivery approach for MET and LFH using a thermosensitive hydrogel made of PLGA‐PEG‐PLGA for SCJ injection is intended to mitigate CNV induced by corneal alkali burns.	The new MET+LFH@Thermogel technology has a lot of potential for treating ocular neovascularization as it offers a confined, effective, and economical method.	·Anti‐CNV	Corneal alkali burn (NaOH)/ Male and female C57BL/6J mice	SCJ injection	[82], 2019
PLGA‐PEG‐PLGA‐Based Thermosensitive Hydrogel	PLGA‐PEG‐PLGA thermogel systems	DEX	A thermogel formulation was designed, comprising DEX encapsulated within PLGA‐PEG‐PLGA, tailored for ocular delivery via the subconjunctival pathway. The goal was to hinder the progression of CNV after ocular injuries induced by alkali.	The thermogel loaded with DEX exhibits prolonged drug release capacity, enabling its utilization in ophthalmic ailments typified by inflammation, thereby reducing the need for frequent SCJ injections.	·Anti‐CNV	Corneal alkali burn (NaOH)/ Female SD rats	SCJ injection	[83], 2020
Nanocomposite Thermosensitive Hydrogel	Thermo‐sensitive hydrogel	Nintedanib	Investigating the impact of thermo‐responsive gel containing nintedanib thermo‐sensitive hydrogel (NTH) on neovascularization and associated indicators in Wistar rats afflicted with corneal alkali injuries.	At body temperature, NTH transforms into a colloidal form, offering the advantage of gradual drug delivery. This property can significantly attenuate CNV induced by alkali burns in rats.	·Anti‐CNV	Corneal alkali burn (NaOH)/ Male Wistar rats	Topical application	[84], 2020
Nanocomposite Thermosensitive Hydrogel	Nanomorphic thermoreversible hydrogel	Nintedanib	To assess and compare the therapeutic efficacy of various formulations of nintedanib for treating CNV resulting from alkali burns in rats.	In comparison to other nintedanib ophthalmic preparations, the nanomorphic thermoreversible hydrogel exhibited superior inhibition of CNV in alkali‐burned eyes and effectively suppressed HUVEC migration and their proangiogenic capacity.	·Anti‐CNV	Corneal alkali burn (NaOH)/ SD rats	Instillation	[85], 2022
Cellular‐Enhanced Thermosensitive Hydrogels	A thermosensitive hyaluronic acid (HA) hydrogel modified with di (ethylene glycol) monomethyl ether methacrylate	miRNA 24‐3p, Exos	To explore the therapeutic capacity of exosomes loaded with miRNA 24‐3p in enhancing the migration and restoration of corneal epithelial cells, particularly in the context of addressing alkali burns of the cornea.	This research demonstrates the effectiveness of a miRNA‐based multilevel delivery strategy using Exos for promoting corneal epithelial regeneration and treating corneal alkali burns, offering a promising approach for cell‐free therapy in ocular surface diseases.	·Promote corneal epithelial healing	Corneal alkali burn (NaOH)/ Male New Zealand white rabbits	Instillation	[86], 2022
PLGA‐PEG‐PLGA‐Based Thermosensitive Hydrogel	A PLGA‐PEG‐PLGA hydrogel	Adalimumab (a TNF‐α inhibitor) and aflibercept (a VEGF inhibitor)	A thermosensitive drug‐delivery system is being developed to sustain the delivery of inhibitors targeting TNF‐α and VEGF. The aim was to provide protection for multiple ocular regions and hinder blood vessel formation after significant ocular trauma.	After severe ocular trauma, the retina and optic nerve are shielded from damage through effective prevention of CNV via a thermosensitive hydrogel delivery system, achieving sustained inhibition of both TNF‐α and VEGF.	·Anti‐CNV	Corneal alkali burn (NaOH)/ Female Dutch‐Belted rabbits	SCJ injection	[87], 2023
CS‐Based Thermosensitive Hydrogel	Poloxamer‐based thermosresponsive *in‐situ* gelling system	HA and indomethacin (IND)	Striving to develop an innovative in‐situ gelling formulation employing poloxamer and HA to improve the management of chemical injuries to the cornea. The design of this formulation will be tailored to react to variations in temperature and facilitate the efficient administration of IND.	The thermoresponsive hydrogel system, which comprises HA and IND, exhibits potential for enhancing the management of ocular chemical burns through the augmentation of drug distribution and stimulation of wound healing processes.	·Promote corneal healing	Corneal alkali burn (NaOH)/ Male New Zealand rabbits	Instillation	[88], 2023
Protein‐Enhanced Hydrogels	A thermo‐sensitive Pluronic F‐127 hydrogel	Progranulin (PGRN)	To investigate the utilization and advancement of a Pluronic F‐127 hydrogel responsive to temperature, containing the protein PGRN, aimed at improving the recovery of corneal tissue post‐alkali burns.	A hydrogel containing Pluronic F‐127, responsive to temperature changes and infused with PGRN, effectively accelerates corneal healing through continuous administration of medication, diminishing inflammation, and fostering tissue regrowth. This presents a hopeful treatment approach for addressing severe corneal damage.	·Promote corneal healing	Corneal alkali burn (NaOH)/ C57BL/6 mice	Instillation	[89], 2023
CS‐Based / Polysaccharide‐Based Thermosensitive Hydrogel	A CS‐based thermosensitive gel carrier	Limbal epithelium stem cells (LESCs)	To utilize 4D bioprinting technology to fabricate a CS‐based thermosensitive hydrogel carrier (4D‐CTH), significantly improving the efficiency of LESCs transplantation and therapeutic outcomes for corneal alkali burns.	4D‐CTH significantly improves LESCs transplantation efficiency, accelerates corneal injury repair, and presents a novel and effective approach for clinical treatment.	·Promote corneal healing	Corneal alkali burn (NaOH)/ Male SD rats	Place on the surface of the injured cornea and close the eyelids with suture	[90], 2024
In Situ Hydrogel	Materials	Loaded Agents						
In Situ Hydrogel	In situ PEG‐based hydrogels	Doxycycline	To design and investigate the efficacy of doxycycline‐loaded PEG hydrogels in healing vesicant‐induced ocular wounds in rabbit corneas.	PEG‐based doxycycline hydrogels significantly enhance corneal wound healing after vesicant injury, providing an effective therapeutic option for ocular mustard injuries	·Promote corneal healing,	Vesicant exposed corneal chemical burn (Half mustard (CEES) and NM)/ rabbit cornea	Instillation to form a film like a contact lens	[91], 2010
In Situ Hydrogel	Two in situ hydrogel‐forming materials (poloxamers 188 and 407)	Gly‐thymosin β4 (Gly‐Tβ_4_)	Assessing the effectiveness of Gly‐thymosin β4 (Gly‐Tβ_4_) in the topical management of corneal alkali injuries, to compare its impact between solution and hydrogel compositions.	Gly‐Tβ_4_ solutions exhibit superior efficacy in promoting corneal recovery compared to hydrogel formulations, attributed to their ability to prevent corneal stimulation, and facilitate rapid release of the therapeutic agent.	·Anti‐CNV ·Promote corneal healing	Corneal alkali burn (NaOH)/ New Zealand albino rabbits	Instillation	[92], 2017
In Situ Hydrogel	In situ Alginate‐CS hydrogel (ACH)	Limbal stem cells (LSCs)	Development of an inventive Hydrogel (ACH) comprising Alginate and CS for on‐site application, aimed at simplifying the transplantation of LSCs with the objective of improving the regeneration of corneal injuries caused by alkali burns.	Employing this inventive approach of in situ hydrogel‐mediated LSC transplantation may present a prompt and effective remedy for enhancing the recovery of corneal wounds following an alkali burn.	·Promote corneal healing	Corneal alkali burn (NaOH)/ Female New Zealand white rabbits	Topical application to form gel and close the eyelids with suture	[93], 2019
In Situ Hydrogel	Thiolated γ‐polyglutamic acid (PGA‐Cys) hydrogel	Keratinocyte Growth Factor (KGF)	Presenting an innovative bioadhesive hydrogel, PGA‐Cys, designed to facilitate the prolonged delivery of KGF, thereby augmenting the process of corneal restoration.	The PGA‐Cys hydrogel remarkably enhances the efficacy of KGF in healing damaged corneas by augmenting bioadhesion and ensuring sustained release. This presents a hopeful strategy for treating corneal injuries.	·Promote corneal healing	Corneal alkali burn (NaOH)+ scrape the corneal debridement with a cotton swab/ Male SD rats	Instillation	[94], 2019
In Situ Hydrogel	Polyethylene glycol‐collagen hydrogels	MSCs	To enhance therapeutic potential for alkali‐induced corneal damage, MSCs are embedded within collagen frameworks cross‐linked with multifunctional PEG‐succinimidyl esters to enable controlled release of the MSCs secretome and mitigate corneal injury caused by alkali burns.	Using PEG‐collagen hydrogels to encapsulate MSCs offers a promising strategy for harnessing their therapeutic advantages in treating inflammatory eye conditions, particularly in cases involving alkali burns.	·Promote corneal healing	Corneal alkali burn (NaOH)/ Rabbit corneas (an ex vivo organ culture model)	Apply to a corneal burn site	[95], 2021
In Situ Hydrogel	In situ‐forming HA hydrogels	HA	Exploring the therapeutic capabilities of in situ‐formed hydrogel structures made from HA, linked via a thiol‐ene response triggered by blue light, in a rat model experiencing corneal damage due to alkali burns.	Leveraging in situ‐synthesized HA hydrogel matrices offers a promising approach for enhancing pharmaceutical transport efficacy in treating corneal injuries.	·Promote corneal healing ·Anti‐CNV ·Anti‐inflammation	Corneal alkali burn (NaOH)/ Male SD rats	Apply to the injured cornea, and induce crosslink in situ using blue light	[96], 2022
Other Hydrogels	Materials	Loaded Agents						
Hydrogel	A chemically modified and cross‐linked derivative of hyaluronan (CMHA‐SX)	/	Investigating the efficacy of a chemically altered and cross‐linked variation of hyaluronan (CMHA‐SX) in expediting the recovery process of corneal epithelial abrasions and alkali burn wounds in rabbit subjects.	The hydrogel CMHA‐SX, when applied topically, expedites the recovery process of corneal epithelial abrasions and alkali burn injuries in rabbits.	·Promote corneal healing	Corneal alkali burn (NaOH)/ Female New Zealand white rabbits	Topical application	[97], 2010
Hydrogel	Recombinant human collagen‐phosphoryl‐choline (RHCIII‐MPC) hydrogels	Recombinant human collagen‐phosphoryl‐choline (RHCIII‐MPC)	Assessing the efficacy of fortified and stabilized hydrogels produced from phosphoryl‐choline‐modified recombinant human collagen (RHCIII‐MPC) as potential substitutes for damaged rabbit corneas subjected to intense alkali injuries.	The repopulation of cells and nerves in alkali‐burned rabbit eyes was aided by bioartificial implants based on RHC. Also, it was observed that RHCIII‐MPC implants have increased neovascularization resistance.	·Promote corneal cell and nerve repopulation ·Anti‐CNV	Corneal alkali burn (NaOH)/ New Zealand White rabbits	Implant in corneas	[98], 2011
Hydrogel	Poly (2‐hydroxyethyl methacrylate) (PHEMA) hydrogel	HA, cationized gelatin, and PEG	Assessing the efficacy of a newly developed T‐style keratoprosthesis derived from PHEMA hydrogel in the restoration of corneal integrity within an animal model exhibiting corneal damage due to alkali burns.	A keratoprosthesis of T‐style featuring selective surface modifications utilizing PHEMA hydrogel presents a hopeful substitute for corneal restoration, amalgamating mechanical durability and enduring integration with the recipient's tissues.	·Promote corneal regeneration	Corneal alkali burn (NaOH)/ Mature male New Zealand rabbits	Implant into the recipient stromal bed with sutures during Lamellar keratoplasty(LKP) 2 months after burn	[99], 2015
Hydrogel	DEX sodium phosphate (Dexp)‐Ava hydrogel	Dexp and Avastin (Ava)	To evaluate a supramolecular hydrogel comprising MPEG‐PCL micelles and α‐yclodextrin, with the objective of concurrently delivering Dexp and Ava to impede neovascularization in a rat model exposed to alkali burn trauma.	To assess the effectiveness of a new supramolecular hydrogel comprising MPEG‐PCL micelles and α‐cyclodextrin, formulated for the simultaneous administration of Dexp and Ava to alleviate neovascularization in a rodent model subjected to alkali burn injury.	·Anti‐CNV ·Anti‐inflammation	Corneal alkali burn (NaOH)/ Male SD rats	Instillation	[100], 2017
Hydrogel	A porous polydimethylsiloxane scaffold loaded with a polyvinyl alcohol (PVA) hydrogel	Infliximab	To explore the effectiveness of continuous subconjunctival administration of infliximab via a polymer‐dependent drug‐delivery mechanism (DDS) in safeguarding the cornea and retina post‐alkali injury to the eye.	The polymer‐based DDS efficiently safeguards the cornea and retina from alkali burn‐induced damage via subconjunctival administration of infliximab.	·Promote corneal healing ·Anti‐inflammation	Corneal alkali burn (NaOH)/ Female Dutch‐Belted rabbits	SCJ delivery/Implantation	[101], 2017
Hydrogel	Gelatin/ascorbic acid cryogel	Ascorbic acid (AA)	Exploring the prospective utilization of cryogels composed of gelatin and AA as vehicles for the advancement of corneal stromal tissue engineering.	The advancement of cryogels composed of gelatin and AA presents a hopeful strategy for tissue engineering in the corneal stroma. The optimal AA concentration improves the regeneration of tissue matrices and sustains transparency, while also reducing corneal harm in an animal model induced with alkali burns.	·Anti‐ROS ·Promote corneal regeneration	Corneal alkali burn (NaOH)/ Adult male New Zealand white rabbits	Intrastromal implantation	[102], 2018
Hydrogel	Gelatin methacryloyl (GelMA) hydrogel	Amniotic extract	To assess the efficacy of amniotic extract‐loaded GelMA hydrogel eye pads in mitigating symblepharon formation in rabbits post ocular alkali injuries.	GelMA eye pads made of hydrogel, which include amniotic extract, effectively prevent symblepharon formation in rabbits by promoting epithelial restoration, reducing inflammation, and providing a stable structure for tissue regrowth.	·Prevent symblepharon ·Anti‐inflammation ·Promote corneal healing	Ocular alkali burn (superior fornix include 1/3 cornea) (NaOH)/ Male New Zealand white rabbits	Placed in the conjunctiva sac	[103], 2020
Hydrogel	A collagen analog (collagen‐like peptide‐polyethylene glycol hybrid, CLP‐PEG)	MPC	To showcase the potential of bioengineered corneal implants that are entirely synthetic, integrating a collagen analogue (a peptide‐polyethylene glycol hybrid resembling collagen, termed CLP‐PEG) along with inflammation‐reducing phosphorylcholine (MPC), in enhancing corneal regeneration and mitigating inflammation in a mini‐pig model with corneal damage induced by alkali burns.	CLP‐PEG‐MPC implants present a hopeful synthetic substitute for corneal regeneration, especially suitable for individuals at an elevated risk of rejecting grafts and justify assessment through clinical trials.	·Promote corneal regeneration ·Anti‐inflammation	Corneal alkali burn (NaOH)/ Female Göttingen mini pigs (Sus scrofa domesticus)	Implant after an anterior LKP (ALK) procedure to replace the anterior 2/3 s of each scarred cornea	[104], 2021
Hydrogel	Monolith/hydrogel	Triamcinolone acetonide (TA)	Developing composites of monoliths/hydrogels to serve as carriers for TA aimed at treating CNV in mice through inhibition of the fibrinolytic system.	The potential therapeutic applications of monolith/hydrogel composites in targeting the fibrinolytic system for inhibiting CNV in mice indicate their development as delivery systems for therapeutic agents. This suggests their potential utility in addressing various biomedical conditions.	·Anti‐CNV	Corneal alkali burn (NaOH)/ Male BALB/c mice	SCJ implantation	[105], 2022
Hydrogel	ROS‐scavenging hydrogel (RH)	PUTK/RH patch	Fabricating a hydrogel‐integrated polyurethane electrospun nanofiber membrane capable of scavenging ROS and assessing its efficacy in the management of ocular alkali burns.	The combined patch can be readily applied to corneas affected by alkali burns, leading to a notable reduction in stromal inflammation and hastening the restoration of corneal clarity.	·Anti‐CNV ·Anti‐inflammation ·Anti‐fibrosis ·Promote corneal healing	Corneal alkali burn (NaOH)/ Female SD rats	Patch treated	[106], 2023
Hydrogel	An injectable HA‐based hydrogel	STB and tannic acid	The development of an injectable hydrogel, based on HA, containing STB and tannic acid, is underway for managing CNV.	The hydrogel, laden with STB, was formulated by integrating tannic acid and polymer NPs. It exhibited potent antioxidant and anti‐angiogenic properties in both controlled laboratory environments and live specimens. This offers a promising approach to tackle corneal disorders, particularly neovascularization and the correlated oxidative burden.	·Anti‐CNV ·Anti‐ROS ·Suppress pro‐inflammation	Corneal alkali burn (NaOH)/ Female SD rats	SCJ injections	[107], 2023
Hydrogel	A novel composite hydrogel	Deoxyribonuclease I (DNase I)	To investigate the application of an innovative composite hydrogel containing DNase I for the regulation of neutrophil extracellular traps (NETs) and the suppression of neovascularization after alkali burns on the cornea.	After corneal alkali burns, the innovative composite hydrogel efficiently regulates NETs and suppresses neovascularization.	·Anti‐CNV	Corneal alkali burn (NaOH)/ Male wildtype C57BL/6J (WT) mice	Hydrogel overlaid on the cornea with eyelid sutured	[108], 2023
Hydrogel	IMB@glycymicelle‐hydrogel	Imatinib (IMB)	To showcase notable therapeutic effectiveness, we aim to create a pioneering ocular delivery system, referred to as IMB@glycymicelle‐hydrogel, designed specifically for the treatment of corneal alkali burn injuries.	IMB@glycymicelle‐hydrogel exhibited encouraging effectiveness in enhancing the healing of corneal wounds, reinstating corneal sensitivity, alleviating opacities, and restraining CNV. These findings indicate its capacity to potentially treat alkali‐induced ocular damage effectively.	·Anti‐CNV ·Promote corneal healing,	Corneal alkali burn (NaOH)/ Male C57BL/6 mice	Instillation	[109], 2024
Hydrogel	HA hydrogel	Epidermal growth factor (EGF) and KGF	Analyzing the combined influence of EGF and KGF delivered via HA hydrogel on the enhancement of corneal wound healing.	Combining EGF and KGF within HA hydrogel offers promising insights into advancing corneal wound healing, suggesting innovative approaches to managing such wounds with potential clinical benefits.	·Promote corneal healing	Corneal alkali burn (NaOH)/ Male SD rats	Add hydrogel solution to eyes and crosslink by blue light irradiation for 40s	[110], 2024
Dendrimers	Materials	Loaded Agents						
Dendrimers	G4‐PAMAM dendrimers	DEX	Examining the effectiveness of a gel comprising dendrimer‐DEX (D‐DEX) for extended application and enhanced treatment of inflammation in the cornea.	An injectable gel containing dendrimer‐DEX shows promise as a targeted and sustained treatment for corneal inflammation, offering enhanced efficacy and reduced side effects compared to free DEX.	·Anti‐inflammation	Corneal alkali burn (NaOH)/ Lewis rats	SCJ injection	[111], 2017
Nanocomplexes	Materials	Loaded Agents						
Nanocomplexes	Nanocomplexes of polyvinylpyrrolidone (PVP)	Kaempferol (KAE)	The objective is to develop and assess an ocular formulation comprising KAE encapsulated in PVP nanocomplexes for treating alkali burns on the cornea.	The innovative formulation 17PF‐KAE shows promise as a novel remedy for preventing and treating eye disorders linked to inflammation and oxidative stress.	·Anti‐inflammation	Corneal alkali burn (NaOH)/ C57BL/6 J mice	Instillation (nano eye drops)	[112], 2020
Nanocomplexes	Tetrahedral DNA nanostructures (TDNs)	Wogonin	Introducing a drug‐delivery system based on multifunctional complexes utilizing a self‐assembling TDN. The focus is on targeted drug delivery and biomedical treatments, emphasizing the design, manufacturing process, and prospective applications.	TDN‐based delivery systems can be a potent therapeutic strategy for treating corneal alkali burns by enhancing cellular uptake, biostability, and targeted delivery of therapeutic agents.	·Promote corneal healing	Corneal alkali burn (NaOH)/ Male New Zealand rabbits	Instillation (eye drops)	[113], 2020
Nanocomplexes	BSA films	KAE	Exploring the utilization of fibrous films made from bovine serum albumin incorporated with KAE as therapeutic contact lenses for delivering ocular medication to address corneal injuries.	BSA/KAE films are a promising platform for therapeutic contact lenses, offering sustained drug release and improved healing for corneal injuries.	·Promote corneal healing ·Anti‐CNV ·Anti‐inflammation	Corneal alkali burn (NaOH)/ Male BALB/c mice	As therapeutic contact lenses	[114], 2021
Nanocomplexes	A pH‐sensitive polymer, mPEG_2k_‐PAMA_30_‐P(DEA_29_‐D5A_29_) (TPPA)	siVEGFA	Introducing an innovative strategy utilizing polymer carriers responsive to pH for the effective transportation of siRNA, with a specific focus on suppressing VEGF‐A to deter CNV.	TPPA stands as a prospective clinical candidate aimed at treating CNV. It offers valuable insights into the rational development of block copolymers tailored for siRNA delivery and therapies targeting angiogenesis inhibition.	·Anti‐CNV	Corneal alkali burn (NaOH)/ Male C57BL/6 mice and BALB/C mice	SCJ injection	[115], 2023
Nanofibers	Materials	Loaded Agents						
Nanofibers	Nanofiber scaffolds prepared from polymer poly (l‐lactid acid) (PLA)	MSCs	To assess the effectiveness of nanofiber scaffold‐supported MSCs in mitigating alkali‐triggered oxidative stress within the cornea and expediting its recovery process.	MSCs grown on nanofiber scaffolds effectively reduce oxidative stress, suppress inflammation and neovascularization, and accelerate corneal healing in alkali‐injured corneas, offering a promising therapeutic approach for corneal injuries.	·Anti‐inflammation ·Anti‐CNV ·Promote corneal healing	Corneal alkali burn (NaOH)/ Female New Zealand white rabbits	Apply on the corneal surface and suture scaffolds to the conjunctiva, and close the eyelids by tarsorrhaphy	[116], 2013
Nanofibers	CS‐modified, collagen‐based biomimetic nanofibrous membranes	/	To design wound dressings for treating chemically burned corneas, the objective is to create biomimetic nanofibrous membranes based on collagen that are modified with CS, facilitating selective cell adhesion.	Biomimetic nanofibrous membranes based on collagen and modified with CS demonstrate potential as advanced wound dressings for chemically burned corneas. These membranes exhibit enhanced transparency, mechanical robustness, and biological efficacy in comparison to conventional dressings made from human amniotic membrane.	·Promote corneal healing	Corneal alkali burn (NaOH)/ Female SD rats	Spread the membranes over the cornea and sutured to the superior and inferior fornix of the conjunctiva	[117], 2014
Nanofibers	PLA nanofibers	MSCs and LSCs	To employ nanofiber scaffolds as a delivery platform for MSCs and LSCs to promote corneal healing and regeneration after ocular surface alkali burn.	Nanofiber scaffolds, serving as a platform to deliver MSCs and LSCs, have the potential to improve the retention and continuous release of MSCs and LSCs at the injury site. This enhances their therapeutic impact by aiding cell engraftment, decreasing inflammation, and fostering corneal regeneration.	·Promote corneal healing	Ocular (whole corneal including the limbal region) alkali burn (NaOH)/ Female New Zealand white rabbits	Cover ocular surface and suture to the conjunctiva	[118], 2015
Nanofibers	PLA nanofibers	Cyclosporine A (CsA)	Investigating the effectiveness of nanofibers loaded with CsA to reduce inflammation and inhibit new blood vessel formation in alkali‐injured rabbit corneas.	Nanofibers loaded with CsA effectively inhibit inflammation, decrease neovascularization, and enhance corneal healing in rabbit corneas injured by alkali exposure.	·Anti‐inflammation ·Promote corneal healing ·Anti‐CNV	Ocular alkali burn (NaOH)/ Female New Zealand white rabbits	Cover ocular surface and suture to the conjunctiva	[119], 2016
Nanofibers	Electrospun bioabsorbable poly(ε‐caprolactone) (PCL) nanofiber mesh	decellularized amniotic membrane (dAM)	Investigating the application of a nanofiber‐enhanced dAM to enhance the effectiveness of limbal stem cell transplantation in treating alkali burn‐induced corneal epithelial defects in rabbit models.	In a rabbit model of limbal stem cell deficiency, the dAM composite membrane reinforced with PCL exhibits improved regenerative and immunomodulatory characteristics. This membrane aids in the transplantation of LSCs, facilitating corneal epithelial healing while decreasing inflammation and preventing neovascularization.	·Promote corneal healing ·Anti‐inflammation ·Anti‐CNV	Corneal alkali burn (NaOH)/ New Zealand white rabbits	Place on the ocular surface and fix to the sclera	[120], 2019
Nanofibers	NapFFHRH (conjugate HRH peptide to NapFF peptide to produce amphiphilic peptides to self‐assemble into nanofibers)	HRH peptide	An innovative and biocompatible nanofiber, incorporating a peptide sourced from VEGF, is presented to enhance the suppression of CNV.	A promising potential is demonstrated by a novel approach based on nanofibers, utilizing an amphiphilic peptide to augment the anti‐angiogenic activity of VEGF peptide, thereby enhancing therapeutic efficacy in ocular vascular diseases.	·Anti‐CNV	Corneal alkali burn (NaOH)/ Male SD rats	SCJ injection	[121], 2021
Nanofibers	Core‐shell nanofibers, with curcumin‐loaded silk fibroin in the core and vancomycin‐loaded CS/PVA in the shell	Curcumin, vancomycin	To create an innovative dual‐drug transporter employing core‐shell nanofibers for managing alkali burns in the cornea, featuring curcumin at the core and vancomycin at the shell.	Core‐shell nanofibers, containing silk fibroin loaded with curcumin in the core and CS/PVA loaded with vancomycin in the shell, demonstrate effectiveness in enhancing the healing of corneal wounds. This presents a hopeful therapeutic strategy for addressing alkali burns on the cornea.	·Promote corneal healing ·Antibacterial activity ·Anti‐inflammation	Corneal alkali burn (NaOH)/ Male New Zealand White rabbits	Ocular insert (fold and place in the inferior fornix and fix in the conjunctiva by suture)	[122], 2023
Nanozymes	Materials	Loaded Agents						
Nanozymes	Cu–Cl MOF (Metal–organic framework)	/	To explore the use of halogen‐coordinated copper‐based metal‐organic frameworks (Cu‐X MOFs) as biomimetic catalysts to treat chemical corneal burns by modulating their enzymatic activity through precise coordination of halogen atoms.	Cu–Cl MOF nanomaterials significantly improve the treatment of alkali‐burn‐induced corneal damage by enhancing antioxidant, anti‐apoptotic, and anti‐inflammatory responses.	·Anti‐inflammation ·Anti‐fibrosis ·Anti‐ROS	Corneal alkali burn (NaOH)/ Female C57BL/6 mice	Instillation (eye drops)	[123], 2023
Nano‐composite materials	Materials	Loaded Agents						
Nano‐composite materials	Carboxymethyl CS (CMCTS)/gelatin (Gel)/HA‐blended membranes	CMCTS	To assess the efficacy of membranes blending CMCTS, Gel, and HA as scaffolds for transplanting corneal epithelial cells, aiming to enhance the healing of corneal wounds.	Blended membranes of CMCTS/Gel/HA demonstrate effective support for the growth of corneal epithelial cells and notably enhance the healing and reconstruction of corneal wounds in a rabbit model of alkali burn injuries.	·Promote corneal wound healing	Corneal alkali burn (NaOH)/ New Zealand white rabbits	Attach to surface of the injured area and close eyelids by suture	[124], 2018
Nano‐composite materials	Dox‐HCM/Col membrane (Doxycycline (Dox), hydroxypropyl CS microspheres (HCMs), and collagen membrane (Col))	Dox	A drug‐loaded collagen membrane (Dox‐HCM/Col) was developed aiming to treat alkali burns of the cornea. The membrane is designed to create a microenvironment conducive to epithelial growth and to release drugs in a controlled manner, thereby expediting the reconstruction of the cornea.	The study developed a novel drug‐loaded collagen membrane (Dox‐HCM/Col) capable of inhibiting MMP activity, promoting corneal re‐epithelialization, and accelerating corneal reconstruction, offering a promising alternative for treating corneal alkali burns.	·Promote corneal healing	Corneal alkali burn (NaOH)/ New Zealand white rabbits (either gender)	Interruptedly suture to the palpebral conjunctiva and sclera	[125], 2023

Nanotechnology has shown promise in treating chemical eye injuries through various experimental models. Common models used to evaluate the efficacy of these treatments typically involve the use of animals, primarily rabbits, and rats/mice, due to the similarity of their eye anatomy and response to injury to that of humans. Key models and approaches in chemical injury research include alkali burn models, acid burn models, and chemical injury models using household chemicals. Alkali burns, particularly those induced by NaOH, are widely utilized due to their ability to mimic severe chemical injuries, penetrating ocular tissues rapidly and causing extensive damage. Acid burns, though less common, provide valuable insights into superficial tissue damage and are instrumental in assessing nanotechnology‐based treatments. Additionally, models employing household chemicals like bleach or ammonia simulate accidental exposures, aiding in the evaluation of nanotechnology's efficacy against everyday chemical injuries. In these models, nanotechnology aims to enhance drug delivery, promote healing, lower inflammation, reduce corneal neovascularization (CNV), and suppress scar formation (Table 4). These models and approaches are crucial for advancing the application of nanotechnology in treating chemical eye injuries, offering the potential for more effective and targeted treatments in the future.

### 3.1 Liposomes

Liposomes stand out as the predominant drug carriers utilized in NP‐assisted drug delivery.^[^ ^126^ ^]^ Furthermore, they are currently the sole NP system sanctioned for clinical application by the U.S. Food and Drug Administration (FDA), owing to their favorable physicochemical characteristics. These characteristics comprise simple production methods and the capability to encase different drugs and molecules, irrespective of their hydrophobic nature, charge, dimensions, and other physicochemical properties, coupled with outstanding compatibility with living tissues.^[^ ^126^ ^]^ Phospholipids, characterized by hydrophobic lipid tails and a polar phosphate head group, spontaneously organize into liposomes through self‐assembly. This is attributed to their amphiphilic nature, allowing them to form bilayer structures in aqueous environments.^[^ ^127^ ^]^ In water‐based settings, the hydrophobic sections of phospholipids arrange themselves, resulting in the formation of a round configuration with a water‐filled center surrounded by a lipid‐based double‐layer membrane. This unique arrangement facilitates the effective carriage of both water‐soluble and lipid‐soluble medications by liposomes. Furthermore, liposomes can be tailored to exhibit compatibility with living tissues and breakdown naturally under particular circumstances, such as changes in pH levels and temperatures, through the modification of their lipid makeup.^[^ ^127^ ^]^

**Figure 4:**
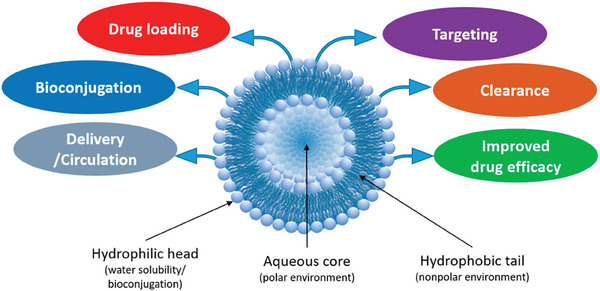
Liposome structural characteristics and their advantages for better medication distribution and therapeutic effectiveness. Reproduced with permmission.^[^ ^126^ ^]^ Copyright 2020, MDPI.

Furthermore, versatile drug‐delivery platforms based on liposomes can be obtained through various modifications (**Figure** **4**).^[^ ^127^ ^]^ Lipid vesicles are preferred for ocular drug transport owing to their capacity to stick to the cornea and effectively convey drugs with low solubility and partition coefficient values.^[^ ^128^ ^]^ Lipid vesicles with a positive charge effectively adhere to the mucin layer, which carries a negative charge, on the corneal epithelium, thus enhancing the absorption of drugs.^[^ ^19^ ^]^

Lipid structures, known for their biodegradability and biocompatibility, facilitate extended drug release onto the ocular surface and deeper penetration into posterior ocular tissues.^[^ ^20^, ^26^, ^129^ ^]^ Multiple studies have demonstrated that liposomes could endow the drug‐delivery platforms with reduced toxicity, improved efficacy, and prolonged release, showcasing significant clinical potential.^[^ ^130^ ^]^ For example, Zhao et al. formulated rapamycin (RAPA) liposomes to inhibit CNV in rats with alkali burns, demonstrating superior therapeutic outcomes compared to traditional formulations.^[^ ^23^ ^]^ RAPA is a lipid‐soluble drug with poor solubility and a narrow therapeutic window and is only available in systemic formulations.^[^ ^131^ ^]^ The low intraocular drug concentration and poor efficacy of systemic administration prevent its widespread use in ophthalmology. Liposomes containing RAPA were prepared using thin‐film hydration with an average diameter of 145.2 nm, and a remarkable encapsulation efficiency of 90.02% was realized.^[^ ^23^ ^]^ Slower growth of CNV was observed after treatment with RAPA‐loaded liposomes, compared with that of a non‐treated group and free RAPA dissolved in bean oil.^[^ ^23^ ^]^ Moreover, the introduction of liposomes could further improve RAPA's biological effects, with remarkable reductions in hypoxia‐inducible factor 1‐alpha levels and vascular endothelial growth factor (VEGF) expression accompanied by non‐immunogenicity, high biocompatibility, increased corneal permeability, and sustained drug release. Similarly, Ambrosone et al. explored liposomal verbascoside eye drops, which showed enhanced corneal retention and expedited healing, highlighting the potential of liposomes for improving the bioavailability and therapeutic effects.^[^ ^24^ ^]^ Verbascoside, known for its strong antioxidant properties, supports the healing of corneal epithelium and diminishes damage caused by exposure to alkali.^[^ ^132^ ^]^ Challenges associated with verbascoside include its rapid elimination when used in conventional eye drops and the necessity for accelerated healing under specific conditions.^[^ ^132d^ ^]^ Ambrosone et al. utilized liposomes to enhance the local retention of verbascoside, resulting in more efficient delivery to the cornea and promoting faster healing within a reduced timeframe.^[^ ^24^ ^]^ Their research illustrates that liposomal verbascoside eye drops effectively enhance corneal epithelial healing, shorten recovery times, and demonstrate beneficial retention effects on the cornea.^[^ ^24^ ^]^ da Silva et al. demonstrated that gelatin membranes containing usnic acid/liposomes (UALs) significantly enhanced tissue regeneration and corneal healing, addressing the challenges associated with usnic acid's poor bioavailability and toxicity.^[^ ^25^ ^]^ Whilst UALs can enhance tissue regeneration and mitigate damage caused by corneal burns,^[^ ^133^ ^]^ usnic acid's drawbacks are its poor bioavailability, solubility, limited interaction with cellular barriers, and significant toxicity.^[^ ^134^ ^]^ The incorporation of liposomes in da Silva et al.’s investigation augmented the bioavailability of UAL, facilitated tissue repair, mitigated corneal burn injuries, and stimulated angiogenesis. Overall, their research shows that the introduction of liposomes expedited corneal healing, presenting a potentially faster and less painful treatment option for ocular surface recovery.

**Figure 5:**
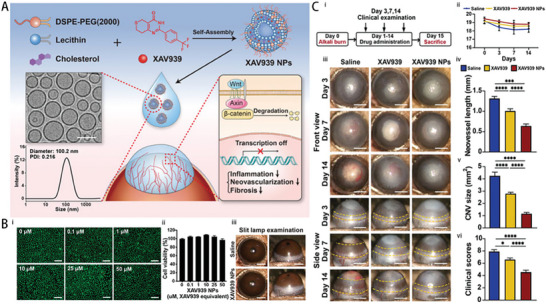
Example of a liposome–drug system, XAV939‐loaded liposomes (XAV939 NPs). A) Diagrammatic representation of the XAV939 NPs' anti‐CNV and corneal wound‐healing properties. B) Biocompatibility assessments. i) Calcein‐AM/PI assay of HCEs with XAV939 NPs at various concentrations (scale bar: 200 µm). ii) CCK‐8 assay of HCEs with XAV939 NPs at different concentrations (mean ± SEM; n = 5). iii) Slit lamp examination of corneas after 14 days of treatment with saline or XAV939 NPs (10 µm XAV939) (scale bar: 1 mm). C) In vivo outcomes on corneal wound recovery and anti‐CNV effects. i) Schematic timeline of the experimental process. ii) Slit lamp images of corneas (anterior and lateral views) at 3, 7, and 14 days post‐treatment, with CNV areas outlined in yellow (scale bar = 1 mm). iii) Mouse weight changes over 14 days of treatment (mean ± SEM; n = 6). iv–vi) Evaluation at 14 days post‐treatment, including neovessel length (iv), CNV size (v), and clinical scoring (vi) (mean ± SEM; *n* = 6; one‐way ANOVA; *p < 0.05, ***p < 0.001, ****p < 0.0001).^[^ ^26^ ^]^ Reproduced with permmission.^[^ ^26^ ^]^ Copyright 2021, Frontiers Media S.A.

In another study, Zhong et al. developed XAV939‐loaded liposomes, which effectively reduced inflammation and angiogenesis by modulating the Wnt/β‐catenin pathway, further supporting the clinical application of liposomes in managing ocular vascular diseases (**Figure** **5**).^[^ ^26^ ^]^ In vivo studies with mice treated using the XAV939‐loaded liposomes showed significant reductions in corneal opacification and edema compared to either XAV939 alone or saline. Moreover, improved corneal wound healing, slower progression, decreased neovessel size, and reductions in choroidal neovascularization size and neovessel length were obtained after treatment. Collectively, these findings highlight the therapeutic potential of liposomes encapsulating XAV939 for treating corneal wounds and suppressing choroidal neovascularization, that provide supportive evidence for the clinical application of liposomes in managing ocular vascular diseases.

In the same year, Lin and co‐workers demonstrated that the use of cationic FK506 liposomes can notably enhance the therapeutic efficacy of FK506 in managing CNV and inflammation, introducing a novel approach to enhance drug bioavailability in ocular delivery systems.^[^ ^27^ ^]^ Despite FK506's inherent anti‐angiogenic and anti‐inflammatory properties, its efficacy is hindered by its short corneal retention period and limited corneal permeability when administered alone.^[^ ^135^ ^]^ By integrating liposomes, enhanced FK506's bioavailability, extended corneal retention time, improved corneal permeability, and amplified therapeutic outcomes were achieved.^[^ ^27^ ^]^ Benefiting from the above‐mentioned characteristics, such liposomal systems could effectively inhibit angiogenesis, reduce inflammation, and promote corneal epithelial healing. In a further example, Diaz‐Palomera and colleagues investigated the physicochemical characteristics and biological outcomes of topical pirfenidone (PFD) liposome‐encapsulated formulation for a corneal alkali injury mouse model.^[^ ^28^ ^]^ PFD possesses antifibrotic, antioxidant, and anti‐inflammatory efficacy in various organs and tissues, as it can inhibit the expression of transforming growth factor beta and attenuates both cell proliferation and migration.^[^ ^136^ ^]^ However, the short half‐life of PFD remains an unsolved problem when considering its topical use to treat chemical corneal burns.^[^ ^28^ ^]^ The limited efficacy of aqueous PFD ocular solutions reflects a short action time and inadequate drug concentration, mainly due to dynamic obstacles within the eye's front portion, such as tear flow and removal through the nasolacrimal duct system.^[^ ^137^ ^]^ The incorporation of liposomes in this system enhanced drug penetration, prolonged ocular surface contact time, improved therapeutic outcomes of topical ophthalmic medications, and decreased administration frequency.^[^ ^136d^ ^]^ Furthermore, extended residence time on the ocular surface and enhanced bioavailability can be achieved. In comparison to PFD in a water‐based solution, the liposomal formulation significantly reduced both the magnitude and frequency of haze, while also mitigating corneal edema, decreasing corneal thickness, and attenuating corneal inflammatory infiltration in mice afflicted with corneal alkali burns.^[^ ^28^ ^]^ In addition, Wang and colleagues investigated the role of Ferrostatin‐1 (Fer‐1)‐loaded liposomes as a therapeutic intervention in corneal injury caused by alkali burns.^[^ ^29^ ^]^ As a specific inhibitor of ferroptosis, Fer‐1 protects corneal cells from damage and reduces angiogenesis, thereby promoting corneal healing. This previous research shows the potential of Fer‐1‐loaded liposomes as a safe and effective alternative treatment for corneal alkali burns, overcoming the challenges posed by Fer‐1′s hydrophobic nature and highlighting new therapeutic avenues targeting ferroptosis.

Overall, these examples of existing research underscore the immense potential of liposomes in ocular drug delivery, offering promising avenues for future research and clinical applications. However, the transition from experimental models to clinical practice requires further investigation, particularly concerning the long‐term safety and efficacy of these liposomal formulations.

### 3.2 Nanoemulsions

Nanoemulsions are biphasic dispersions where droplets of oil are stabilized in water (O/W) or vice versa (W/O) by an amphiphilic substance.^[^ ^136a^ ^]^ As a highly promising class of nanocarriers, particularly for drugs with low water solubility,^[^ ^32^ ^]^ nanoemulsions offer several numerous advantages over traditional eye drops, such as enhanced drug penetration, reduced toxicity and irritation, improved stability, increased drug loading capability, controlled drug release, rapid drug absorption, extended therapeutic efficacy, and significantly enhanced bioavailability.^[^ ^138^ ^]^

Nanoemulsions, with particle sizes ranging from 5 to 200 nm, exhibit strong thermodynamic stability and primarily penetrate the cornea,^[^ ^139^ ^]^ making them an ideal candidate for ocular drug delivery. Furthermore, the nanoemulsion sterilization process is just as simple as it is for traditional solutions.^[^ ^140^ ^]^ These advantages have increased research into nanoemulsions for ocular treatments.^[^ ^141^ ^]^ Ocular delivery of O/W nanoemulsions is formulated for transporting drugs that are sensitive to the environment, with poor solubility in water, limited absorption via the transcorneal route, or shorter retention times.^[^ ^140^ ^]^ Transparency, viscosity, and the refractive index require special consideration for ophthalmic nanoemulsions. Moreover, different doses of nanoemulsions should be titrated to evaluate their tolerability.

One persistent challenge is the treatment of anterior segment neovascularization, particularly in the cornea.^[^ ^31^ ^]^ While anti‐VEGF therapies have shown promising results for neovascularization in the posterior eye, these treatments rely on biological agents that require frequent intravitreal injections. This poses significant difficulties for patients, increases the risk of endophthalmitis, requires specialized personnel for administration, and places a considerable financial burden on healthcare systems.^[^ ^31^ ^]^

Several studies have explored the potential of nanoemulsions in treating ocular neovascularization. For instance, Ma and colleagues developed a microemulsion (ME) containing naringenin (NAR) (NAR‐ME),^[^ ^142^ ^]^ a compound that inhibits CNV, but this faces solubility and bioavailability challenges.^[^ ^30^ ^]^ Most notably, the integration of the ME substantially increased the solubility of NAR and its permeability through the cornea, resulting in enhanced bioavailability and effectiveness in therapy. Overall, the optimized NAR‐ME exhibited favorable physicochemical characteristics and demonstrated tolerability, indicating its potential as a secure and effective treatment approach for CNV.^[^ ^30^ ^]^

**Figure 6:**
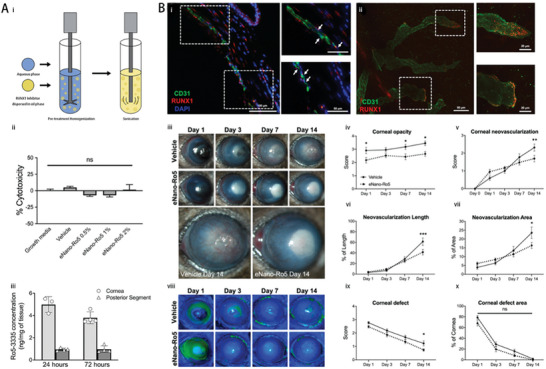
Nanoemulsion‐drug platforms for RUNX1 suppression in ocular neovascularization studies. A) Safety and ocular pharmacokinetics of eNano‐Ro5. i) eNano‐Ro5 production via homogenization and sonication. ii) Bar graph showing minimal cell mortality with different eNano‐Ro5 concentrations. iii) Ro5‐3335 levels in cornea and posterior segment after topical eNano‐Ro5 application (mean ± SD, *n* = 3,4). B) Improved outcomes in an alkali‐burn CNV model with eNano‐Ro5. i) Immunofluorescence showing RUNX1+ and CD31+ cells in corneal sections (scale bars = 100 µm, 50 µm^2^). ii) Confocal imaging of CD31 and RUNX1 in CNVs (scale bars = 50 µm, 20 µm). iii) Images of treated mice over 1, 3, 7, and 14 days. iv–vii) Graphs showing reduction in corneal opacity and neovascularization with eNano‐Ro5 (mean ± SEM, vehicle *n* = 22; eNano‐Ro5 *n* = 23, two‐way ANOVA *p < 0.05, **p < 0.01, ***p < 0.001). viii) Sodium fluorescein staining shows corneal ulcer healing. ix,x) Graphs showing improvement in corneal re‐epithelization after eNano‐Ro5 treatment. DAPI, 4′,6‐diamidino‐2‐phenylindole. Reproduced with permission.^[^ ^31^ ^]^ Copyright 2022, Elsevier.

In another study, Delgado‐Tirado et al. formulated a nanoemulsion formulation incorporating Ro5‐3335 (eNano‐Ro5) to inhibit runt‐related transcription factor 1 (RUNX1) in a model of ocular neovascularization (**Figure** **6**).^[^ ^31^ ^]^ RUNX1 suppression, achieved through small interfering RNA (siRNA) and small molecule inhibitors, significantly reduced angiogenic processes such as migration, proliferation, and capillary formation in vascular endothelial cells.^[^ ^143^ ^]^ Previous therapies struggled due to the transient effects of kinase inhibitors and poor antibody penetration across ocular barriers.^[^ ^144^ ^]^ Ro5‐3335, a small and lipophilic molecule, was designed to target transcription factors, offering sustained downstream impacts. Liquid chromatography‐tandem mass spectrometry confirmed the delivery of Ro5‐3335 to the cornea and posterior ocular tissues via eNano‐Ro5, with a retention time of 5.17 minutes.^[^ ^31^ ^]^ The nanoemulsion effectively reduced cell proliferation, cytotoxicity, and migration in human retinal endothelial cells and showed significant efficacy in mouse models of corneal and choroidal neovascularization. Topical application of eNano‐Ro5 significantly reduced corneal opacity and neovascularization post‐alkali burn and decreased lesion size and leakage in choroidal neovascularization. These findings suggest that non‐invasive, topical RUNX1 inhibitors like eNano‐Ro5 hold potential for treating pathologic ocular neovascularization.^[^ ^31^ ^]^

Zhang and colleagues developed an innovative isoliquiritigenin (ISL)‐loaded nanoemulsion (ISL‐NE) to treat CNV in a mouse model of corneal alkali burns.^[^ ^32^ ^]^ Despite ISL's poor water solubility and low bioavailability, the nanoemulsion significantly enhanced ocular penetration and therapeutic efficacy. The formulation, consisting of propylene glycol dicaprylate (PGD), Cremophor® EL (EL35), polyethylene glycol 400 (PEG 400), aqueous solution, and sodium hyaluronate, forms a particle size of 34.56 ± 0.80 nm with a narrow size distribution.^[^ ^32^ ^]^ No toxicity was observed in human corneal epithelial cells and rabbit eyes. ISL‐NE showed sustained drug release and improved corneal penetration compared to ISL suspension (ISL‐Susp), while also demonstrating greater bioavailability in the aqueous humor, cornea, and tears.^[^ ^32^ ^]^ In mouse models, ISL‐NE effectively reduced VEGF‐A and matrix metalloproteinase‐2 (MMP‐2) expression, similar to dexamethasone (DEX) treatment,^[^ ^32^ ^]^ suggesting its potential as a safe and effective therapy for CNV. Lastly, Shi et al. developed a topical sunitinib (STB) microemulsion (STB‐ME) eye drop for inhibiting CNV.^[^ ^33^ ^]^ STB, a receptor tyrosine kinase inhibitor,^[^ ^145^ ^]^ is limited by poor solubility in water, low dissolution rate, and limited permeability.^[^ ^33^ ^]^ These authors demonstrated that STB‐ME showed robust stability and bioavailability, effectively suppressing CNV when applied topically, indicating a promising drug‐delivery system for treating CNV.

### 3.3 Nanomicelles

Following the discussion on liposomes, another key nanoparticle system in ophthalmic drug delivery is nanomicelles. Ranging from 5 to 200 nm in size, nanomicelles are colloidal carrier systems formed by amphiphilic surfactants (which may be cationic, zwitterionic, or anionic in nature) or diblock polymers.^[^ ^146^ ^]^ Micelles have the capacity to adopt different geometries, including cylindrical, spherical, or star‐shaped structures, influenced by variables such as the molecular weight of the core and the constituents constituting the corona.^[^ ^147^ ^]^ The configuration of micelles, whether normal or reverse, is contingent upon the organization of amphiphilic molecules. In a conventional micelle, the core is composed of hydrophobic components, while hydrophilic components protrude outward into the aqueous medium, facilitating the transportation of hydrophobic medications. In contrast, in a reverse micelle, the core comprises hydrophilic constituents, whereas the corona is constituted by hydrophobic components, facilitating the transportation of hydrophilic drugs.^[^ ^148^ ^]^

These properties of nanomicelles make them highly advantageous in ophthalmic drug‐delivery systems. Firstly, their small size and versatile shapes allow for more effective penetration of ocular tissues while also protecting drugs from degradation, thereby increasing bioavailability and therapeutic efficacy. Secondly, composed of amphiphilic surfactants or polymers, nanomicelles can form either normal or reverse micelles, enabling the efficient delivery of both hydrophobic and hydrophilic drugs. Additionally, nanomicelles enhance the solubility of hydrophobic drugs in aqueous solutions, improving their dispersion and effectiveness within the eye.^[^ ^148^, ^149^ ^]^

**Figure 7:**
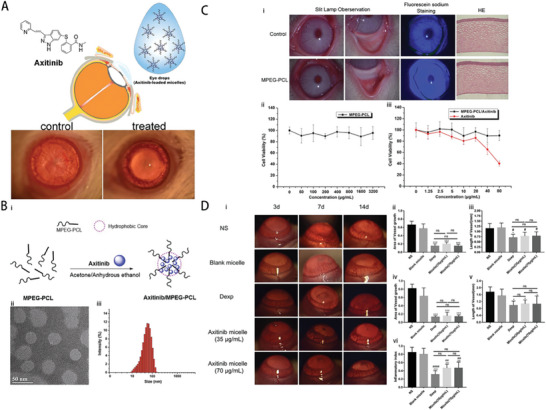
An example micelle–drug system based on axitinib‐loaded MPEG‐PCL micelles. A) Schematic of self‐assembled axitinib‐loaded micelles in eye drops. B, i) Overview of axitinib‐loaded MPEG‐PCL micelle preparation. ii, iii) Size distribution analysis using TEM and a particle‐size analyzer. C) Biocompatibility evaluation: i) Slit lamp and fluorescein staining observations, and HE staining of treated corneas; ii) Cytotoxicity assessment of MPEG‐PCL on cells; iii) Cytotoxicity comparison of axitinib alone and combined with MPEG‐PCL. D, i) Slit lamp images showing inhibitory effects on CNV in vivo; ii, iii) Neovascular area and blood vessel measurement after 3 days of treatment; iv,v) Measurement after 7 days; vi) Early‐stage inflammatory scoring. NS, normal saline; Dexp, DEX sodium phosphate. Reproduced with permission.^[^ ^34^ ^]^ Copyright 2019, Elsevier.

In one notable study, Shi et al. developed axitinib‐loaded amphiphilic block polymer polyethylene glycol‐polycaprolactone (MPEG‐PCL) micelles, that enhanced their water dispersibility (**Figure** **7**).^[^ ^34^ ^]^ Axitinib, a hydrophobic tyrosine kinase inhibitor used in clinical oncology, inhibits VEGF receptor (VEGFR) signaling. Their investigation revealed that optimal drug‐loading efficiency is achieved when the lengths of the hydrophilic and hydrophobic chains in the amphiphile block polymer are approximately equal. Moreover, the micelles loaded with axitinib showed low toxicity, had minimal impact on normal cell migration, and exhibited excellent cell compatibility. Thus, the axitinib‐loaded micelles serve as effective anti‐angiogenic agents and hold promise for treating CNV induced by alkali burns.

Another investigation by Song et al. explored the application of ultra‐small nanomicelles made from Rebaudioside A (RA) to enhance the bioavailability and efficacy of pterostillbene (Pt) for ocular drug delivery.^[^ ^35^ ^]^ RA tends to form very small micelles, which could improve drug delivery. While the medicinal efficacy of RA micelles is still largely unexplored, antioxidant properties of Pt is important for anti‐inflammatory and antiproliferative effects make it a promising candidate. The study found that encapsulating Pt in RA micelles significantly enhanced its antioxidant properties, likely due to improved solubility in aqueous solutions.^[^ ^35^ ^]^ The RA‐Pt micelles, with an average size of 3.99 ± 0.03 nm and a low polydispersity index (PDI = 0.184 ± 0.008), showed increased antioxidant efficacy in vitro and better ocular permeability.^[^ ^35^ ^]^ RA‐Pt demonstrated superior intraocular penetration and anti‐inflammatory effects compared to free Pt, particularly in treating alkali burn‐induced corneal injuries in vivo. These results underline the potential of RA‐based nanomicelles to enhance the therapeutic efficacy and ocular bioavailability of poorly soluble drugs like Pt.

**Figure 8:**
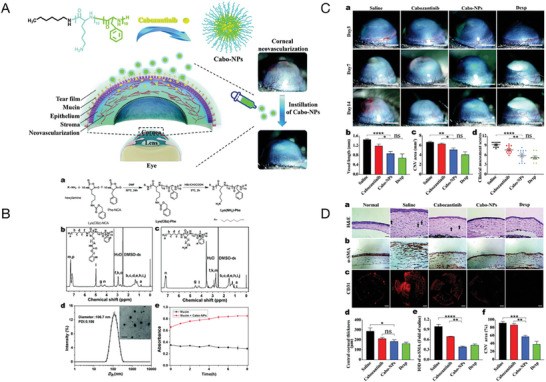
An example micelle–drug system based on cationic polypeptide micelles encapsulating lipophilic compound cabozantinib (cationic Cabo‐NPs). A) Schematic representation of the preparation of mucoadhesive Cabo‐NPs and the processes involved in inhibiting CNV. B) Synthesis and characteristics of Cabo‐NPs. C,D) Photographs and clinical assessment scores demonstrating Cabo‐NP treatment effectiveness on alkali‐burned corneas, highlighting its anti‐CNV efficiency. Reproduced with permission.^[^ ^36^ ^]^ Copyright 2020, Royal Society of Chemistry.

Further advancements in nanomicelle drug delivery have been demonstrated by Han et al., who developed cationic polypeptide micelles (Cabozantinib (Cabo)‐NPs) for treating CNV (**Figure** **8**).^[^ ^36^ ^]^ These micelles enable the sustained release of Cabo and improve adhesion to the corneal surface with significantly enhanced bioavailability.^[^ ^150^ ^]^ Cabo‐NPs effectively inhibit angiogenesis and inflammation, addressing the limitations of Cabo's poor corneal penetration in topical applications.^[^ ^151^ ^]^ By providing a safe and efficient delivery system, Cabo‐NPs overcome the challenge of poor drug penetration through the corneal barrier. Preclinical models have shown that this approach enhances therapeutic outcomes, making cationic polypeptide micelles a promising alternative for CNV treatment. Further clinical translation of this method could further improve CNV management.^[^ ^36^ ^]^

Traditional eye‐drop formulations often lack efficiency due to rapid tear drainage and the barrier posed the corneal epithelium. While topical glucocorticoid therapy is a common treatment for CNV, it faces limitations such as rapid tear replacement, poor absorption, and steroid‐related side effects like glaucoma^[^ ^152^ ^]^ and cataracts^[^ ^153^ ^]^. To address these issues, Zhang et al. developed a bioadhesive nanoplatform using glycosylated boric acid chemistry to enhance the trans‐corneal delivery of corticosteroids, offering a more effective strategy for managing CNV. This innovative platform utilizes a synthetic amphiphilic copolymer based on boric acid to form stable micelles with a high DEX‐loading capacity.^[^ ^37^ ^]^ The boric acid modules provide strong bioadhension, allowing prolonged contact with the corneal epithelium and controlled drug release over 96 hours. In vitro experiments demonstrated the effective uptake of the micelles by corneal epithelial cells. Furthermore, in vivo studies showed enhanced drug delivery to the corneal stroma through transcytosis. When applied topically to alkali‐induced corneal burns in rats, the nano‐formulation significantly reduced inflammation and inhibited neovascularization compared to unencapsulated DEX, with minimal disruption to healthy tissue. This bioadhesive approach, designed to extend drug retention and improve delivery across the cornea, offers a promising noninvasive treatment for CNV and could serve as a flexible platform for other biomedical applications by overcoming protective barriers.

Further innovations have focused on improving the solubility of poorly soluble ocular drugs, as demonstrated by Li et al. These authors developed a nanocarrier system using dipotassium glycyrrhizinate (DG), a salt derived from glycyrrhizin, to encapsulate rebamipide (RBM) for treating corneal alkali burns.^[^ ^38^ ^]^ Glycyrrhizin, an FDA‐approved drug, has anti‐inflammatory and ROS‐scavenging properties, making it a promising candidate for the treatment of ocular diseases.^[^ ^154^ ^]^ However, RBM, despite also being FDA‐approved, suffers from poor aqueous solubility and limited ocular permeability, reducing its efficacy in treating corneal alkali burns.^[^ ^155^ ^]^ The DG‐RBM particles developed by Li et al. showed high encapsulation efficiency and significantly reduced micellar size, greatly enhancing the aqueous solubility of RBM by a factor of 3.1 × 10^5^ compared to its free form. Both in vitro and in vivo studies confirmed the safety and improved therapeutic efficacy of DG‐RBM in treating alkali‐induced corneal burns. This improvement is attributed to the modulation of high‐mobility group box 1 (HMGB1) signaling and the regulation of angiogenic and proinflammatory cytokines. In conclusion, DG, as a nanocarrier for RBM, offers a promising topical treatment for corneal alkali burns by targeting HMGB1 signaling pathways.^[^ ^38^ ^]^

Expanding on the potential of micelles for inflammation control, Thathapudi et al. developed a SmartCelle™ micelle system to encapsulated TA‐A001, a cannabinoid receptor 2 (CB2r) agonist, for treating moderate corneal alkali burns.^[^ ^39^ ^]^ Compared with prednisolone, a corticosteroid, TA‐A001 demonstrated enhanced solubility and delivery when incorporated into micelles. Their study found that micelle‐encapsulated TA‐A001 promoted faster corneal wound healing than prednisolone, highlighting its potential as an alternative to corticosteroids for managing corneal inflammation. However, higher doses of TA‐A001 induced neovascularization, indicating the need for precise dose optimization.^[^ ^39^ ^]^ Further research is required to fine‐tune dosing and assess long‐term outcomes.

Finally, Sun et al. introduced an innovative empagliflozin (EMP)@glycymicelle ophthalmic solution, utilizing glycyrrhizin as a nanocarrier for EMP to treat corneal alkali burns.^[^ ^40^ ^]^ Both EMP and glycyrrhizin inhibit HMGB1 signaling, reducing oxidative stress, inflammation, and neovascularization while promoting corneal wound‐healing. EMP, an FDA‐approved drug for type 2 diabetes, also demonstrates strong anti‐apoptosis, anti‐angiogenic, antioxidant, and anti‐inflammatory properties. However, EMP's poor aqueous solubility limits ocular applications. To address this, glycyrrhizin was used as an amphiphilic nanocarrier to form glycymicelles, improving EMP's solubility, ocular bioavailability, and therapeutic efficacy. The nano‐formulation enhanced wound healing and reduced inflammation and neovascularization, making the EMP@glycymicelle ophthalmic solution a promising approach for treating corneal alkali burns.^[^ ^40^ ^]^

In conclusion, nanomicelles have emerged as versatile and effective drug‐delivery systems for ophthalmic applications, particularly in overcoming the challenges associated with ocular drug administration. Their small size and ability to carry both hydrophobic and hydrophilic drugs enable improved tissue penetration, drug stability, and bioavailability. Numerous studies have demonstrated their potential to enhance the therapeutic efficacy of drugs in treating conditions like CNV and alkali‐induced injuries. With further optimization and clinical translation, nanomicelles hold great promise for advancing the treatment of various ocular diseases, offering novel, noninvasive therapeutic options that could significantly improve patient outcomes.

### 3.4 Nanowafers (NWs)

As summarized above, researchers have explored various systems like liposomes, nanoemulsions, and nanomicelles to enhance the efficacy of ocular drug delivery. Some progress has been made through the introduction of these formulations, although there are still some challenges regarding rapid clearance from the ocular surface, which limits their therapeutic effects.^[^ ^156^ ^]^ Contact lenses loaded with drugs were developed as an alternative to prolong drug retention, but even these systems struggled to sustain drug release over long periods, leading to reduced efficacy and sometimes causing allergic reactions.^[^ ^157^ ^]^

Nanowafers offer several unique advantages over contact lenses in drug delivery. Unlike contact lenses, which primarily extend drug retention but still require frequent reapplication, NWs are designed to provide prolonged and controlled drug release. These miniature transparent discs contain nanoreservoirs that gradually dispense medication directly onto the ocular surface, allowing for continuous drug absorption into the surrounding tissues. NWs also adhere firmly to the eye, even during blinking, and gradually dissolve in tear fluids, ensuring sustained drug release over an extended period without the need for frequent administration.^[^ ^158^ ^]^ This makes NWs particularly effective for maintaining consistent therapeutic levels in ocular tissues.

**Figure 9:**
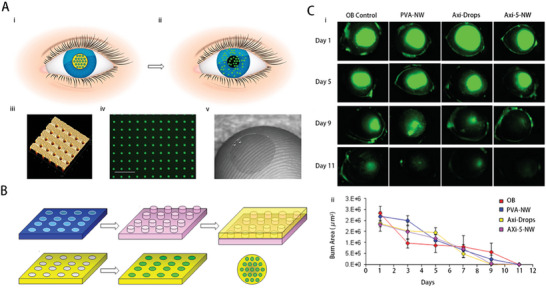
An example NW–drug system based on axitinib. A, i, ii) Schematic illustration depicting the attachment of the NW with drugs onto the cornea (i) and subsequent diffusion into the cornea (ii); iii) An array of nanoreservoirs with a diameter of 500 nm are seen in an AFM image of a NW; iv) A NW loaded with doxycycline is shown in a fluorescence micrograph (scale bar = 5 µm); v) Photo of a NW—a small circular disc—placed delicately on the tip of a fingertip. B) Schematic of the NW fabrication process. C) Administration of axitinib NWs enhance the recovery of corneal burns; i) Fluorescence images of corneas; ii) Chart illustrating corneal surface healing measured by fluorescence intensity. Error bars depict the standard deviation from the mean (*n* = 3). Reproduced with permission.^[^ ^41^ ^]^ Copyright 2015, American Chemical Society.

One study by Yuan and colleagues highlighted the potential of NWs for enhancing therapeutic efficacy.^[^ ^41^ ^]^ They developed a NW drug‐delivery system containing axitinib, an anti‐angiogenic agent, and tested its effectiveness in a murine model of CNV (**Figure** **9**). After screening various polymers, these researchers identified PVA as a suitable, non‐stimulatory material for fabricating the ocular NWs that could effectively deliver axitinib.^[^ ^41^ ^]^ Their study found that daily administration of axitinib NWs provided comparable results to twice‐daily eye‐drop therapy; in a corneal alkali burn model, the NW demonstrated superior efficacy to traditional eye drops, inhibiting CNV and suppressing pro‐inflammatory and pro‐angiogenic factors. This enhanced performance was attributed to the prolonged residence time of the NW on the ocular surface, allowing for better drug diffusion. Additionally, the nanowafer gradually dissolves, ensuring efficient drug delivery without invasive procedures. This system offers a novel and effective method for ocular drug administration, combining simplicity and therapeutic efficacy.

Further advancements were made by Bian et al., who developed DEX‐loaded NWs (DEX‐NW) to treat mice with alkali‐induced eye injury and desiccating stress.^[^ ^42^ ^]^ The NW formulation showed significant reductions in corneal opacity and levels of pro‐inflammatory markers (IL‐1b, IL‐6, and MMP‐9) compared to controls. Moreover, decreased neutrophil infiltration and lower myeloperoxidase (MPO) activity were also observed. Once‐daily DEX‐NW treatment gave comparable efficacy as four‐times daily administration for improved corneal clarity and reduced inflammation, and also minimized neutrophil infiltration mainly due to the sustained release of DEX. Overall, the introduction of NWs highly improved clinical outcomes and patient adherence, suggesting an efficient and convenient method for managing ocular inflammation.

A similar example was demonstrated by Ammassam Veettil and colleagues, who developed a dextran sulfate‐based polymer wafer (DS‐wafer) to control inflammation and fibrosis in corneal injury models.^[^ ^43^ ^]^ Their study, using both corneal abrasion and alkali burn mouse models, demonstrated that the DS‐wafer effectively inhibited scarring, neovascularization, and fibrosis without the need for external anti‐inflammatory agents.^[^ ^43^ ^]^ Fabricated through electrospinning, the DS‐wafer leverages the properties of dextran sulfate (DS), a biopolymer like glycosaminoglycans, known for its solubility, stability, and therapeutic potential.^[^ ^159^, ^160^ ^]^ Their study found that DS‐wafer treatment significantly reduced corneal inflammation and fibrosis, scarring, and opacity compared to untreated corneas or those treated with unsulfated dextran wafers. This polymer therapy, devoid of pharmaceutical additives, displayed potent anti‐inflammatory and antifibrotic effects, making the DS‐wafer a promising approach for inhibiting CNV and fibrosis while promoting wound healing.

NWs represent a breakthrough in ocular drug delivery, addressing many of the limitations seen in traditional methods like eye drops, contact lenses, and other NP‐based systems. The existing research demonstrates the versatility of this technology in delivering both hydrophobic and hydrophilic drugs, reducing the frequency of administration, and improving patient adherence. As the development of NW systems continues, this technology holds great promise for advancing ocular therapeutics and improving clinical outcomes for patients with ocular surface diseases.

### 3.5 Nanostructured Lipid Carriers (NLCs)

**Figure 10:**
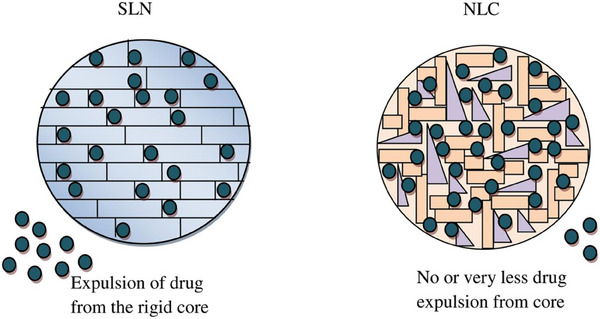
Stability of SLN and NLC over time. Reproduced with permission.^[^ ^161^ ^]^ Copyright 2019, Elsevier.

NLCs, a significant advancement in lipid‐based NP technology, consist of a combination of lipids, surfactants, and therapeutic agents. NLCs present distinct advantages over their predecessors, solid lipid NPs (SLNs), primarily due to their enhanced biocompatibility and stability (**Figure** **10**).^[^ ^161^ ^]^ NLC development aimed to overcome obstacles such as drug seepage from the lipid matrix during storage and poor drug‐loading efficiency.^[^ ^162^ ^]^ The production of NLCs utilizes different approaches including cold homogenization, ultrasonication following hot emulsification, and hot homogenization techniques.^[^ ^161^ ^]^ NLCs demonstrate the most promising outcomes owing to their enhanced biocompatibility for ocular administration.^[^ ^162^ ^]^ They exhibit modified drug release kinetics along with being non‐immunogenic, biologically non‐toxic, and biocompatible. Given the lipophilic nature of numerous medications, their compatibility with lipids and their solubility become crucial elements in NLC formulation.^[^ ^161^ ^]^

In a key application of this technology, Xing and colleagues developed a novel drug‐delivery strategy using NLCs to administer RAPA for treating corneal injuries caused by alkaline burns.^[^ ^44^ ^]^ RAPA, a potent mTOR inhibitor, has demonstrated immunosuppressive, anti‐cancer, anti‐aging, anti‐inflammatory, and antifibrotic properties; however, its hydrophobic nature and sensitivity to environmental factors such as temperature, light, and pH^[^ ^163^ ^]^ limit its stability in aqueous formulations.^[^ ^164^ ^]^ NLCs were chosen as the delivery platform to enhance RAPA's stability and corneal permeability. Compared to SLNs and vesicular constructs, NLCs offer better drug protection, higher loading capacity, and greater physical and chemical stability.^[^ ^165^ ^]^ In Xing et al.’s study, RAPA was efficiently delivered to the cornea using NLCs composed of precirol ATO5, capryol PGMC, and stearylamine. The NLCs had a spherical morphology with a moderately positive surface charge, leading to a 1.5‐fold increase in C6 absorption by fibroblasts.^[^ ^44^ ^]^ The RAPA‐loaded NLCs also suppressed TGFβ1 and PDGF expression in corneal fibroblasts, inhibiting myofibroblast formation, collagen deposition, and angiogenesis. These findings suggest that RAPA‐loaded NLCs are a promising therapeutic option for reducing corneal haze and fibrosis following chemical burns.^[^ ^44^ ^]^

Similarly, Selvaraj et al. developed NLCs loaded with itraconazole (ITR) and coated them with chitosan (CS) (CS‐ITR‐NLCs) to topically treat diabetic retinopathy (DR) and CNV.^[^ ^45^ ^]^ ITR, an anti‐angiogenic agent, inhibited VEGF165 and CNV,^[^ ^166^ ^]^ but the application was limited by low ocular permeability and bioavailability. The NLCs were designed to improve the stability and permeability of the loaded active pharmaceutical ingredient, thereby enhancing its bioavailability in ocular tissues. CS coating further increased adhesion and controlled drug release. This formulation demonstrated efficacy in inhibiting both DR and CNV, showing potential as a treatment for these conditions.^[^ ^45^ ^]^

In another example of NLC innovation, Li and his colleagues developed a NLC to encapsulate dasatinib, a tyrosine kinase inhibitor known for inhibiting the Src family of kinases and platelet‐derived growth factor receptors, showing potential for treating CNV.^[^ ^46^ ^]^ The NLC formulation containing dasatinib had a particle size of approximately 78 nm with a low polydispersity index, improving dasatinib's solubility by over 1220 times compared to its free form. Moreover, prolonged drug release, reduced ocular toxicity, and enhanced corneal permeation were obtained. This approach also suppressed key processes involved in CNV development, including cell growth, migration, and tube formation in HUVEC cells.^[^ ^46^ ^]^ In a mouse model of alkali burn‐induced CNV, significantly inhibited CNV progression and reduced pathological changes in the cornea were observed, suggesting its promise as a therapeutic strategy for CNV.^[^ ^46^ ^]^

Finally, advancing the application of NLCs for CNV treatment, Luo and colleagues formulated NLCs containing sorafenib (SRB) to enhance its delivery to the ocular surface, specifically targeting CNV.^[^ ^47^ ^]^ SRB, a multikinase inhibitor, targets VEGFR1‐3 and PDGFR, disrupting proangiogenic signaling pathways and effectively inhibiting CNV.^[^ ^167^ ^]^ The SRB‐NLCs improved the solubility, bioavailability, and corneal penetration of sorafenib while providing sustained drug release.^[^ ^47^ ^]^ The formulation demonstrated excellent physicochemical properties, biocompatibility, and prolonged drug release, making it a promising therapeutic option for CNV. These advancements address the limitations of traditional drug‐delivery methods, offering potential for improved CNV management.

In summary, NLCs offer a powerful platform for enhancing the delivery of hydrophobic and unstable drugs to ocular tissues. Through the use of biocompatible lipids and innovative encapsulation techniques, NLCs have demonstrated significant advantages over traditional lipid‐based delivery systems, including improved drug stability, enhanced permeability, and prolonged release profiles. Research has shown the advantages of NLCs for treating various ocular conditions, such as corneal burns, neovascularization, and diabetic retinopathy, and as a versatile and effective drug‐delivery method, NLCs show great potential for advancing ophthalmic treatments.

### 3.6 Nanoparticles (NPs)

NPs have emerged as a promising class of colloidal carriers for drug delivery due to their versatile properties. Ranging from 10 to 1000 nm in size, NPs have shown promise in ocular drug delivery.^[^ ^168^ ^]^ In recent research, NPs between 50 and 400 nm have proven especially effective because they can bypass anatomical and physiological barriers, offering precise targeting of tissues and cells. Their ability to exploit both passive and ligand‐mediated mechanisms facilitates delivery to specific ocular regions.^[^ ^169^ ^]^ NPs employed for transporting substances to the anterior and posterior segments of the eye consist of proteins and peptides (including albumin and gelatin), lipids (such as triglycerides and fatty acids), natural materials (like sodium alginate and CS, synthetic polymers (such as poly(lactide‐co‐glycolide), polylactic acid (PLA), and polycaprolactone).^[^ ^19^, ^170^ ^]^ Utilizing NPs offers numerous advantages including their ability to transport medications that are both lipophilic^[^ ^171^ ^]^ and hydrophilic^[^ ^172^ ^]^. This results in a reduced frequency of dosing because of the sustained release of drugs. Furthermore, with advancements in material sciences, NPs enhance drug absorption by facilitating cellular uptake and enable targeted delivery to tissues.^[^ ^168^, ^173^ ^]^ Several ocular diseases, including corneal alkali burn, have been successfully treated using NP‐mediated medication delivery.

#### 3.6.1 Peptide and Protein NPs

In the field of peptide and protein‐based NPs, one notable study by Jani et al. focused on albumin NP as a delivery vehicle for pCMV.Flt23K gene therapy. These NPs, after undergoing lyophilization for convenient storage, were administered to mice three weeks post‐surgical alkali injury.^[^ ^48^ ^]^ They demonstrated that the albumin NPs could efficiently enter the cytoplasm of corneal keratocytes without causing toxicity and remain in the corneal tissue for up to four weeks, maintaining effective interceptor levels for up to five weeks. This prolonged retention allowed for sustained gene expression and successfully reducing CNV by ≈40% compared to controls (*p* = 0.035) at the 5‐week time point. This study marks the first instance of sustained gene transfer by NPs in the cornea.^[^ ^48^ ^]^ This breakthrough offers new hope for treating ocular conditions requiring long‐term intervention, especially by reducing the frequency of drug administration while maintaining efficacy.

**Figure 11:**
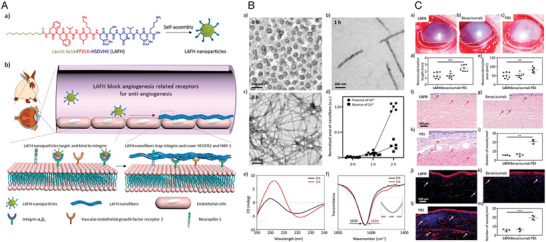
An example of a monotargeting pepnetibody. A, a) Molecular structure of LAFH along with a depiction of LAFH NPs; b) A schematic representation showing how LAFH NPs attach to integrin α_v_β_3_ on endothelial cell surfaces, leading to the formation of LAFH nanofibers. These nanofibers trap integrin α_v_β_3_ and obscure other receptors related to angiogenesis, such as VEGF receptor 2 and neuropilin‐1, ultimately inhibiting neovascularization in a CNV rabbit model. B) LAFH NPs interact with Ca^2+^, leading to their transformation into nanofibers characterized by β‐sheet structures; a–c) TEM images depict LAFH NPs incubated with CaCl_2_, showing a spherical shape at 0 h (a), a combination of spherical and fibrous shapes at 1 h (b), and a predominantly fibrous shape at 2 h (c); d) A quantitative assessment of the normalized area of nanofibers from TEM images of LAFH NPs, both with and without CaCl_2_ incubation, indicates that binding induces fibrillogenesis; e) Circular dichroism (CD) and f) Fourier‐transform infrared (FT‐IR) spectra of LAFH NPs incubated with CaCl_2_ at 0 and 2 h, respectively, reveal the development of a β‐sheet structure. C) In a CNV rabbit model in vivo, LAFH impedes the process of angiogenesis. Reproduced with permission.^[^ ^49^ ^]^ Copyright 2021, American Chemical Society.

Further expanding on NP research, Zhang et al. introduced a novel therapeutic approach using a monotargeting peptidic network antibody (pepnetibody) to treat CNV (**Figure** **11**).^[^ ^49^ ^]^ This approach utilizes a binding‐induced fibrillogenesis peptide (BIF peptide) that forms a fibrous network on endothelial cell membranes, blocking multiple angiogenesis‐related receptors, including integrin α_v_β_3_, VEGFR2, and NRP‐1,^[^ ^49^ ^]^ which effectively inhibits CNV progression. Although some angiogenic pathways may not be targeted, the pepnetibody demonstrated competitive efficacy compared to the monoclonal antibody bevacizumab (Beva), achieving similar anti‐angiogenic effects at a 97.7‐times lower dosage. This study highlights the potential of nanoscale structural design in developing efficient anti‐angiogenic therapies for CNV and related diseases.^[^ ^49^ ^]^

Continuing the exploration of novel NP‐based treatments, Chu et al. explored a novel NP‐based treatment for CNV using GNP‐gp91, a gelatin NP encapsulating the gp91 peptide.^[^ ^50^ ^]^ The gp91 peptide inhibits vessel formation by targeting Nox2 and reducing oxidative stress‐mediated angiogenesis. The developed NPs, with a positive charge, improve corneal retention and drug permeability, offering sustained release for prolonged therapeutic effects.^[^ ^174^ ^]^ In a chemically induced CNV animal model, reduced vessel formation was obtained through the administration of GNP‐gp91 eye drops.^[^ ^175^ ^]^ This formulation presents a promising therapeutic option for CNV, with the advantage of infrequent dosing and convenient topical application for patients.^[^ ^50^ ^]^

Furthermore, Xu and colleagues devised a novel approach for producing metal–protein NPs by coordinating zinc ions with Beva, namely Beva‐Zn^2+^ NPs.^[^ ^51^ ^]^ Beva, a VEGF inhibitor, is commonly used to treat ocular neovascularization but faces challenges such as poor tissue penetration, low encapsulation efficiency, and reduced stability. The formation of Beva‐Zn^2+^ NPs overcome the limitations of Beva (i.e., low stability, poor bioavailability, and limited therapeutic efficacy) and further demonstrate that this facile and universal strategy for generating protein–metal NPs could effectively inhibit CNV induced by alkali burns, offering potential for broader therapeutic applications.

#### 3.6.2 Lipid NPs

Lipid‐based NPs have emerged as promising tools for ocular drug delivery, particularly in the treatment of CNV. Taketani and colleagues developed lipid NPs encapsulating a modified short hairpin RNA (Li‐pshRNA) targeting angiopoietin‐like protein 2 (ANGPTL2),^[^ ^176^ ^]^ a proangiogenic and proinflammatory factor involved in CNV and ocular inflammation.^[^ ^52^ ^]^ In a chemically induced mouse model, ANGPTL2 Li‐pshRNA effectively suppressed CNV by inhibiting ANGPTL2 expression. While RNA interference therapies like Li‐pshRNA face challenges in achieving efficient delivery and may trigger immune responses,^[^ ^177^ ^]^ the use of lipid NPs enhances tissue distribution and penetration. This approach offers a promising therapeutic strategy for managing CNV and ocular inflammation with potentially fewer side effects than current treatments.^[^ ^52^ ^]^

**Figure 12:**
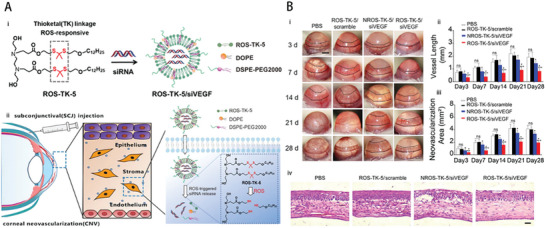
An example lipid NP–drug system based on siRNA for treating CNV. A) Manufacturing process, treatment, and the mechanism; i) ROS‐TK‐5 encapsulated siVEGF via charge–charge interactions, mediated by the thioketal (TK) linkage; ii) In a mouse model of corneal alkali burn‐induced CNV, SCJ injection of ROS‐TK‐5/siVEGF NPs targets the stromal layer, releasing siRNA in response to ROS levels, inducing gene silencing and reducing CNV. B) Inhibition of CNV by ROS‐TK‐5/siVEGF NPs; i) Images of mouse corneas at 3, 7, 14, 21, and 28 days post‐treatment with new blood vessels marketed by dotted lines; ii, iii) Graphs showing vessel length and CNV area over time (mean ± SD; *n* = 24; *p < 0.05, **p < 0.01, ***p < 0.001 compared to PBS, #*p* < 0.05, between ROS‐TK‐1/siVEGF and NROS‐TK‐1/siVEGF); Scale bar = 1 mm. Histological analysis of corneal slices stained with H&E on day 7 (× 400, *n* = 12); Scale bar = 100 µm. Reproduced with permission.^[^ ^53^ ^]^ Copyright 2022, American Chemical Society.

In a complementary study, Liu and colleagues developed a reactive oxygen species (ROS)–responsive lipid NP to deliver siRNA into corneal lesions as a potential therapy for CNV (**Figure** **12**A).^[^ ^53^ ^]^ siRNA is a powerful method for gene silencing, but its hydrophilic nature and negative charge limit cellular uptake. This study demonstrated that elevated ROS levels in CNV facilitated the degradation of the lipid NPs, enabling effective siRNA delivery into human umbilical vein endothelial cells and enhancing gene silencing. In an alkali burn model, subconjunctival (SCJ) injection of these siRNA nanocomplexes significantly reduced VEGF expression, inhibiting CNV formation (Figure 12B).^[^ ^53^ ^]^ This ROS‐sensitive lipid NP approach shows promise as a potential therapeutic strategy for CNV.

In summary, lipid NPs present a versatile and promising platform for delivering RNA‐based therapies, including shRNA and siRNA, to treat CNV and related inflammatory conditions. Both Taketani and Liu's studies demonstrate the enhanced therapeutic potential of this approach for overcoming traditional barriers in ocular drug delivery, such as poor penetration and immune activation. Their ability to modulate gene expression and suppress angiogenic factors positions lipid NPs as an innovative and effective strategy for managing ocular diseases like CNV.

#### 3.6.3 Lipoprotein NPs

Lipoprotein NPs have shown great promise in ocular drug delivery, particularly for targeting intervention for various ocular pathologies. Early studies revealed that the absence of very low‐density lipoprotein receptors (VLDLR) could activate Wnt signaling, contributing to abnormal blood vessel formation in the retina.^[^ ^54^ ^]^ Building on this, Wang and colleagues devised NPs encapsulating the VLDLR extracellular domain (VLN) (VLN‐NP) using poly(lactic‐co‐glycoic acid) (PLGA) as a carrier. PLGA is widely used in drug delivery for its sustained release, biodegradability, and protection of plasmid DNA.^[^ ^178^ ^]^ The results demonstrated that VLN‐NPs allowed for sustained release and expression of VLN over 4 weeks post‐injection in both cultured cells and the retina.^[^ ^54^ ^]^ In models of oxygen‐induced retinopathy and alkali burn‐induced CNV, key markers of Wnt signaling, including the phosphorylation of low‐density lipoprotein receptor‐related protein 6, β‐catenin accumulation, and VEGF, were suppressed.^[^ ^54^ ^]^

High‐density lipoprotein (HDL) NPs have emerged as a versatile platform for treating ocular surface diseases, offering multifunctional therapeutic capabilities.^[^ ^179^ ^]^ Synthetic HDL NPs are engineered to closely mimic the structural and functional properties of native HDL, including the presence of apolipoprotein A‐I (apoA‐I), which binds with high affinity to the scavenger receptor class B type 1 (SR‐B1), a key player in cellular cholesterol metabolism and targeted drug delivery. Furthermore, HDL NPs can be chemically tailored to adjust both surface and core compositions, providing precise control over their therapeutic functions. Besides their extraordinary biocompatibility, the topical administration of HDL NPs to the ocular surface has shown promise in promoting corneal re‐epithelialization after injury and reducing inflammation caused by chemical burns and other stressors. Additionally, HDL NPs function as efficient delivery vehicles for transporting bioactive microRNAs to corneal cells, offering targeted therapeutic benefits.^[^ ^179^ ^]^ These attributes suggest that HDL NPs may play a crucial role in the treatment of ocular surface injuries and inflammation by combining tissue repair with anti‐inflammatory effects.

MicroRNAs have emerged as potential therapeutic agents due to their ability to influence various biological processes. However, their clinical application is limited by challenges related to delivery and stability. Recent studies have shown that synthetic HDL NPs can efficiently deliver therapeutic agents to ocular tissues upon topical application.^[^ ^55^ ^]^ Wang et al. demonstrated that HDL NPs, including those loaded with microRNAs (miR‐HDL NPs), are efficiently taken up by corneal and limbal epithelial cells, as well as stromal keratocytes, when applied to the intact ocular surface.^[^ ^55^ ^]^ Their research revealed that topical application of HDL NPs or miR‐HDL NPs significantly enhanced corneal re‐epithelialization in diabetic mice compared to controls. On the 7th day post‐injury, corneas treated with HDL NPs showed a 40–50% improvement in opacity and surface integrity (*p* < 0.05) compared to those treated with PBS or control NPs. Additionally, HDL NP‐treated corneas exhibited a well‐organized epithelium and stroma, with collagen bundles arranged in a structured manner, reduced inflammatory cell counts, and decreased prominence of stromal keratocytes. In models of alkali burn‐induced inflammation, topical HDL NP treatment resulted in notable clinical, morphological, and immunological improvements.^[^ ^55^ ^]^ These findings highlight HDL NPs as a promising delivery system for microRNAs, offering significant benefits for patients with compromised wound healing, such as those with diabetes or conditions like dry eye syndrome or chemical burns. The application of HDL NPs represents a novel and effective approach for enhancing ocular tissue repair and managing corneal inflammations.

In conclusion, lipoprotein NPs, including VLN and HDL‐based formulations, represent promising therapeutic strategies for addressing neovascularization and corneal inflammation, particularly in the context of ocular chemical burns. Their capacity to target specific molecular pathways, such as Wnt signaling, and efficiently deliver therapeutic molecules like miRNA underscores their potential to revolutionize the treatment of ocular surface diseases. By enhancing wound healing and mitigating inflammation, lipoprotein NPs offer significant benefits, especially for patients facing challenges such as CNV, impaired diabetic wound healing, and inflammation‐induced ocular pathologies. These advancements highlight the potential of lipoprotein NPs to serve as innovative solutions in the evolving landscape of ocular therapeutics.

#### 3.6.4 Glycopolymeric NPs (CS)

CS, a polymer obtained through the deacetylation process of chitin, a complex carbohydrate, is also known as poly‐2‐amino‐2‐deoxy‐D‐glucose. This polymer has widespread application in different nano‐drug‐delivery platforms owing to its multitude of benefits, such as improved compatibility with biological systems, biodegradability, decreased potential for triggering immune responses, and absence of toxicity.^[^ ^180^ ^]^ Furthermore, the positive charge present on CS's surface can effectively engage with the negative charge found in the mucin layer of the ocular surface, fostering molecular attraction, and thereby augmenting mucosal adhesion. This leads to the extension of drug retention on the ocular surface and the amplification of bioavailability.

Kanwar et al. investigated the use of CS‐based NPs in an ex vivo bovine corneal alkali burn model.^[^ ^56^ ^]^ The NPs, crosslinked with sodium tripolyphosphate (STPP), demonstrated potential for treating alkali burns and ocular injuries, although further study is needed to fully assess their clinical application. Further research showed that combining the histone deacetylase inhibitor trichostatin‐A (TSA) and the dominant negative survivin protein (SurR9‐C84A), delivered via ultra‐small CS NPs, improved wound healing, and reduced inflammation in a rat alkali burn model.^[^ ^57^ ^]^

A study by Zahir‐Jouzdani et al. explored the anti‐fibrotic and anti‐angiogenic potential of CS and thiolated–CS NPs.^[^ ^58^ ^]^ They demonstrated that both types of NP could inhibit fibroblast proliferation, myofibroblast differentiation, and neovascularization in a corneal injury model, largely through the suppression of profibrotic cytokines such as TGFβ1 and PDGF. Their study suggest that these NPs could be developed as cost‐effective and non‐toxic treatments to prevent corneal haze, fibrosis, and blindness following chemical injuries to the eye.^[^ ^58^ ^]^

Song et al. synthesized bromfenac‐loaded CS NPs for the treatment of alkali burn‐induced CNV.^[^ ^59^ ^]^ Bromfenac sodium (BF), a non‐steroidal anti‐inflammatory drug (NSAID), was selected for its pain‐relief and anti‐inflammatory properties.^[^ ^59^ ^]^ Nevertheless, BF's effectiveness in treating ocular conditions is constrained by its short duration of action, high frequency of dosing, low patient adherence, and insufficient drug concentration in deep ocular tissues.^[^ ^181^ ^]^ The NPs, created via an ion crosslinking method, exhibited sustained‐release characteristics, allowing for prolonged drug action for up to 48 h. In the rabbit CNV model, the bromfenac‐loaded CS NPs effectively reduced neovascularization and suppressed the expression of pro‐inflammatory markers like cyclooxygenase‐2 and VEGF mRNA. This study underscores the potential of CS‐based NPs for the treatment of CNV by delivering anti‐inflammatory agents with enhanced efficacy.

**Figure 13:**
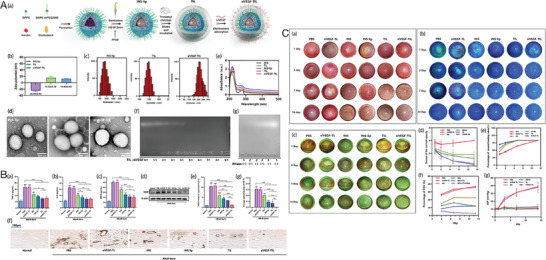
An example of liposome‐trimethyl CS (TMC) NPs to treat corneal chemical burns. A) Preparation and characterization of NPs; a) Illustration outlining the synthesis approach for siVEGF‐TMC‐INS‐liposome (siVEGF‐TIL); b) Zeta potential measurements and c) NP sizes of INS‐lip, TIL, and siVEGF‐TIL (*n* = 3 per group); d) Transmission electron microscopy (TEM) images depicting INS‐lip, TIL, and siVEGF‐TIL (scale bar = 100 nm); e) Ultraviolet (UV)–visible–near infrared (NIR) spectra of INS, TL, INS‐lip, TIL, and siVEGF‐TIL; f) Evaluation of loading capacity using agarose gel electrophoresis, indicating the amount of unbound siVEGF. The quantity of unbound siVEGF decreases with an increasing TIL/siVEGF mass ratio; g) RNase protection assay conducted via agarose gel electrophoresis. siVEGF‐TIL (siVEGF‐trimethyl CS‐coated insulin liposome) is compared with INS‐lip (insulin liposome), TIL (trimethyl CS‐coated insulin liposome), 1 (siVEGF), 2 (siVEGF‐TIL), and 3 (siVEGF‐TIL subjected to shaking for 2 h at 4 °C). B In vivo inhibition of inflammation and neovascularization by NPs. C Clinical evaluation of healing in a corneal alkali burn rat model. Reproduced with permission.^[^ ^60^ ^]^ Copyright 2023, Wiley.

Finally, Xiong et al. explored a novel strategy using liposome‐trimethyl CS NPs to co‐deliver insulin (INS) and siVEGF for the treatment of corneal alkali burns (**Figure** **13**).^[^ ^60^ ^]^ INS, known for its antioxidant properties and ability to promote corneal nerve repair,^[^ ^182^ ^]^ was combined with siVEGF, a molecule that inhibits VEGF to prevent abnormal angiogenesis.^[^ ^68^, ^183^ ^]^ The NPs, acting as carriers, improved the stability of INS and siVEGF while enabling their sustained release at the injury site.^[^ ^184^ ^]^ The results showed significant improvements in wound healing, reduced inflammation, and the inhibition of CNV.^[^ ^185^ ^]^ This study suggests that this dual‐delivery system offers a promising approach to treating severe corneal injuries with long‐term therapeutic effects.^[^ ^60^ ^]^

In summary, CS‐based NPs offer a promising solution for ocular drug delivery thanks to their biocompatibility, biodegradability, and ability to enhance drug retention on the ocular surface. Studies have shown their effectiveness in treating corneal injuries by delivering therapeutic agents, reducing inflammation, and inhibiting fibrosis and neovascularization. These NPs provide sustained drug release and are a potential non‐toxic, cost‐effective option for improving corneal healing, especially in cases involving chemical burns.

#### 3.6.5 Synthetic Polymers

##### Poly(Lactic‐Co‐Glycolic Acid) (PLGA)

PLGA NPs have emerged as a promising platform for ocular drug delivery due to their biocompatibility, biodegradability, and ability to encapsulate a variety of therapeutic agents. Qazi and colleagues developed PLGA NPs loaded with pSEC.shRNA–VEGF‐A plasmids using a double emulsion‐solvent evaporation technique.^[^ ^61^ ^]^ The VEGF‐A gene plays a crucial role in promoting angiogenesis, including vascular endothelial cell migration, proliferation, and increased vascular permeability, all of which contribute to CNV.^[^ ^186^ ^]^ By targeting VEGF‐A expression,^[^ ^187^ ^]^ these NPs effectively reduce CNV, which is a key factor in ocular surface injuries such as chemical burns.^[^ ^188^ ^]^ Although previous studies demonstrated that plasmids encoding small hairpin RNA targeting VEGF‐A can inhibit and even reverse CNV, their therapeutic effects have been limited by the short‐term stability of naked plasmids.^[^ ^189^ ^]^ PLGA NPs offer a solution by providing sustained delivery, protecting the plasmids from degradation, and facilitating their efficient absorption into corneal tissue without triggering immune responses.^[^ ^48^ ^]^ The slow‐release kinetics and high drug‐loading capacity of PLGA NPs enable prolonged therapeutic effects, which are essential for treating chronic ocular conditions. These NPs can deliver not only pharmacological agents^[^ ^190^ ^]^ but also siRNAs^[^ ^191^ ^]^ over extended periods, making them a versatile tool for drug delivery.^[^ ^192^ ^]^ Importantly, the non‐cationic nature of PLGA NPs reduces the toxicity often associated with cationic polymers, further enhancing their safety profile for ocular use. While preclinical studies suggest that PLGA NPs hold significant promise for clinical applications, their full therapeutic potential requires further validation in human trials.^[^ ^193^ ^]^ The pSEC.shRNA–VEGF‐A‐loaded PLGA NPs offer a potent, safe, non‐viral, and sustained approach to gene therapy for reducing ocular neovascularization caused by mechanical alkali injury.^[^ ^61^ ^]^

Chowdhury and colleagues developed PLGA‐based NPs encapsulating PFD to address key clinical concerns following alkali‐induced ocular injuries, such as impaired corneal epithelial regeneration and scarring.^[^ ^62^ ^]^ PFD is known to inhibit TGF‐β‐induced collagen I and alpha‐smooth muscle actin (α‐SMA) synthesis in corneal fibroblasts, reducing myofibroblast formation and collagen production. However, its therapeutic efficacy is limited due to its short half‐life after topical application, necessitating frequent dosing.^[^ ^10b^ ^]^ To address this, these researchers designed biodegradable NPs for sustained release. The NPs demonstrated efficient encapsulation (102.04 ± 66.92 mg/mg of particles) and rapid uptake by corneal fibroblasts within 5 minutes, remaining active for approximately 30 minutes. In an alkali burn model, PFD‐loaded NPs significantly reduced collagen I levels, corneal opacity, and healing time compared to unencapsulated PFD, which had minimal effects. These NPs show potential not only for enhancing wound healing and reducing fibrosis after corneal burns but also for treating other corneal fibrotic conditions.^[^ ^62^ ^]^

Tsai and colleagues developed PLGA NPs to improve the bioavailability of lingzhi (LZH), a traditional Chinese herbal medicine known for its antioxidative and anti‐inflammatory properties.^[^ ^63^ ^]^ LZH has been widely used in clinical treatments for chronic diseases, but like many topical agents, it suffers from low bioavailability when administered via eye drops—typically less than 5%.^[^ ^156^, ^194^ ^]^ To address this issue, LZH‐loaded NPs were created, averaging 184 nm in size, and were found to enhance intracellular retention of LZH in corneal epithelial cells. Chemiluminescence assays demonstrated that the LZH NPs reduced ROS levels, which also decreased inflammation and apoptosis in oxidatively damaged corneal epithelial cells, thereby improving cell viability. In vivo studies using fluorescein and H&E staining confirmed that LZH NPs accelerated wound healing and reduced inflammation in alkali‐burned corneas. These findings suggest that PLGA NPs are an effective delivery system for LZH and hold potential for treating corneal injuries such as alkali burns.

**Figure 14:**
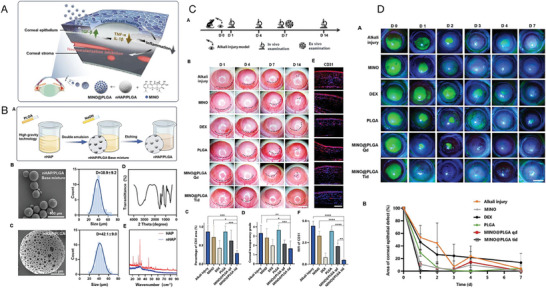
An example drug‐delivery method based on MINO‐loaded nHAP combined with PLGA to enhance the effectiveness of CNV treatment. A) Graphical abstract. B) Schematic diagram depicting the fabrication of nHAP/PLGA in (A); Panels (B) and (C) present Scanning electron microscopy images of the base mixture of nHAP/PLGA along with the corresponding sizes; Section D Fourier transform infrared spectroscopy (FT‐IR) characterization of nHAP; E X‐ray diffraction characterization of both nHAP and HAP. C) CNV is inhibited, and haze is reduced by MINO@PLGA. D) Changes in the area of corneal epithelial injury observed among various groups following an alkali burn. Reproduced with permission.^[^ ^64^ ^]^ Copyright 2024, The Authors.

Li and coauthors developed a novel drug‐delivery system using NPs composed of nano‐hydroxyapatite (nHAP)/PLGA to deliver minocycline (MINO) for the treatment of CNV (**Figure** **14**).^[^ ^64^ ^]^ MINO, known for its anti‐inflammatory properties, has limited bioavailability when applied to the cornea due to its unique structure.^[^ ^195^ ^]^ By incorporation PLGA, the system provides sustained drug release, enhancing MINO's ocular compatibility and efficacy. This sustained release mechanism ensures prolonged therapeutic effects, maintaining optimal drug levels at the treatment site and minimizing adverse reactions. In vivo studies confirmed the effectiveness of the MINO@PLGA nanocomplex in suppressing CNV with low cytotoxicity and strong safety, making it a promising approach for managing CNV‐related ocular conditions.^[^ ^64^ ^]^

##### Polyethylene Glycol (PEG)

PEG NPs have been utilized to improve the solubility and delivery of hydrophobic drugs. Lee et al. investigated PEG NPs loaded with apatinib for inhibiting VEGF‐induced angiogenesis and mitigating CNV.^[^ ^66^ ^]^ Apatinib, a selective inhibitor of VEGF receptor 2 (VEGFR2), works by blocking VEGF‐driven processes in endothelial cells, such as proliferation, migration, and tube formation. However, apatinib's poor water solubility limits its ocular delivery and distribution within the hydrophilic corneal stroma, reducing its therapeutic efficacy.^[^ ^66^ ^]^ To overcome this, the study utilized human serum albumin (HSA)‐PEG NPs to encapsulate apatinib, significantly improving its solubility, biocompatibility, and biodistribution. This delivery system enhanced drug availability in the corneal stroma, showing potential as a treatment for CNV. The findings suggest that HSA‐PEG NPs could be an effective platform for delivering apatinib to prevent and treat CNV.^[^ ^66^ ^]^

Yin and colleagues evaluated the efficacy of a novel nano‐prodrug, DEX‐PEG‐APRPG (stands for Ala‐Pro‐Arg‐Pro‐Gly) (DPA), for targeted treatment of CNV, where the APRPG represents the peptide sequence Ala‐Pro‐Arg‐Pro‐Gly.^[^ ^67^ ^]^ DEX, the key component, acts as an anti‐inflammatory agent to inhibit CNV. However, DEX alone suffers from poor water solubility and limited bioavailability in ocular tissues, requiring frequent dosing and posing risks such as corneal ulcers and glaucoma. To address these issues, the team developed a nano‐prodrug by modifying DEX with APRPG and PEG. This formulation self‐assembles into NPs that enhance DEX's water solubility, target endothelial cells, and inhibit cellular migration and tube formation.^[^ ^67^ ^]^ Additionally, the NP system prolongs drug retention in the eye, allowing for reduced dosage and fewer side effects. This study demonstrates that the DPA nano‐prodrug offers a targeted, effective, and safe approach for treating CNV, providing significant advantages over traditional therapies.

##### Polyethylenimine (PEI)

PEI NPs have been explored for delivering siRNA to target genes involved in corneal scarring and inflammation. Zahir‐Jouzdani and colleagues developed a topical delivery system using TGF‐β_1_ siRNA aimed at reducing dibrosis and inflammation after corneal injuries.^[^ ^68^ ^]^ TGF‐β_1_ is a key factor in inflammation‐induced corneal scarring, often leading to vision loss. The siRNA targets and degrades specific mRNA within the RNA‐induced silencing complex, effectively down‐regulating TGF‐β_1_ expression ^[^ ^196^ ^]^. However, siRNA's negative charge and instability pose challenges for delivery. By encapsulating TGF‐β1 siRNA in PEI NPs, stability and cellular uptake were enhanced. In corneal fibroblast cultures, TGF‐β_1_ siRNA NPs reduced the expression of fibrotic and angiogenic genes, inhibiting fibroblast proliferation. In a murine corneal burn model, topical application of TGF‐β_1_ siRNA NPs effectively reduced fibrosis and angiogenesis, showing promise as a treatment for corneal haze and neovascularization after chemical injury.

In summary, synthetic polymers such as PLGA, PEG, and PEI offer versatile and effective platforms for ocular drug delivery, enhancing the stability, solubility, and targeted delivery of therapeutic agents. These advancements provide significant benefits for treating corneal injuries and diseases, with potential applications in clinical settings.

#### 3.6.6 Liquid Crystalline NPs (LCNPs)

**Figure 15:**
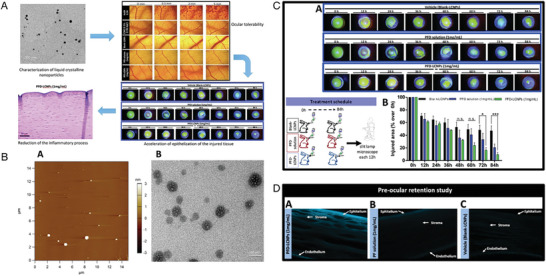
An example of a LCNPs–drug system incorporating PFD to treat corneal chemical burns. A) Graphical abstract. B) Using polarized light microscopy, a photomicrograph captures a liquid crystal with a hexagonal structure. C) Impact of 1 mg mL^−1^ PFD LCNPs on corneal recovery following a chemical injury. D) Pre‐ocular retention indicates that PFD LCNPs facilitate extended drug contact duration on the corneal surface: A) PFD LCNPs at a concentration of 1 mg mL^−1^; B) PFD solution (100 µL) at a concentration of 1 mg mL^−1^, and C) a group treated exclusively with the vehicle (blank LCNPs). Reproduced with permission.^[^ ^69^ ^]^ Copyright 2019, Elsevier.

Silva et al. developed LCNPs incorporating PFD to enhance ocular drug delivery, particularly for corneal chemical burns (**Figure** **15**).^[^ ^69^ ^]^ Despite PFD's short half‐life following topical application, it has shown benefits for corneal wound healing. To address the delivery challenges, monolein (MO) was used as the lipid component due to its non‐toxic, biodegradable, and biocompatible properties, forming well‐organized liquid crystals upon contact with water. MO's liquid crystalline properties allow for controlled release, bio‐adhesion, and the ability to encapsulate hydrophilic and hydrophobic drugs.^[^ ^197^ ^]^ This enhances drug retention at the absorption site and increases interaction with the corneal epithelium.^[^ ^198^ ^]^ In vivo studies using PFD LCNPs on rabbits demonstrated sustained drug release compared to PFD in solution. The nano‐sized, spherical particles showed minimal ocular irritation and good stability at 4 °C. When applied at 1 mg mL^−1^, PFD LCNPs reduced corneal lesions and shortened re‐epithelialization time compared to the control (blank LCNPs). Histological analysis also indicated a reduction in inflammatory cells and decreased MPO activity. These results suggest that PFD LCNPs offer a promising approach for sustained drug delivery in treating corneal injuries.

#### 3.6.7 Inorganic NPs

Among the various inorganic NPs, silica NPs (SiNPs) have emerged as highly promising due to their unique properties, offering numerous advantages. These include a hydrophilic surface that promotes prolonged circulation within the body, exceptional biocompatibility, the flexibility of silane chemistry, the ease of synthesizing them on a large scale, and their cost‐effectiveness in production.^[^ ^199^ ^]^ Silicate, a compound abundant in nature, has been widely employed in the treatment of various diseases, including cancer, cardiovascular conditions,^[^ ^200^ ^]^ Parkinson's disease,^[^ ^201^ ^]^ and choroidal neovascularization.^[^ ^202^ ^]^ SiNPs serve as adaptable carriers for a diverse range of therapeutic substances, including small‐molecule medications, photosensitizers used in photodynamic therapy, peptides, proteins, DNA, and RNA.

Building on this, Mohammadpour and his team were the first to demonstrate the efficacy of topical SiNPs in reducing CNV after a chemical burn.^[^ ^70^ ^]^ After treatment the vascularized cornea area significantly reduced from 85% to 21% with decreased extent of intrastromal vascularization.^[^ ^70^ ^]^ SiNPs are therefore a useful method for preventing CNV after a chemical burn in an experimental context. Further investigation is now necessary to evaluate the effectiveness and safety of this approach for use in human ocular treatments.

To enhance the delivery of anti‐angiogenic agents, Sun et al. developed a formulation using mesoporous SiNPs (MSNs) encapsulating Beva.^[^ ^71^ ^]^ The use of Beva is hindered by its short half‐life, requiring frequent injections, which may lead to poor adherence and increase the risk of ocular complications such as endophthalmitis, elevated IOP, hemorrhage, and retinal detachment.^[^ ^203^ ^]^ Incorporating nanomaterials into Beva extends its residence time in the eye, enabling sustained release, improving efficacy against VEGF‐induced endothelial processes and new blood vessel formation, and ensuring safety with minimal cytotoxicity or tissue toxicity.^[^ ^204^ ^]^ This study introduced an innovative approach using Beva NPs encapsulated in MSN to enhance the effectiveness, duration, and safety of antiangiogenic therapy for ocular conditions. These findings underscore the potential of MSNs as a drug‐delivery system for enhancing antiangiogenic treatments.^[^ ^71^ ^]^

Chen et al. explored the use of cerium oxide NPs (CeNPs) for the treatment of CNV caused by alkali burns.^[^ ^72^ ^]^ They demonstrated that CeNPs, featuring an optimized ratio of Ce^3+^ to Ce^4+^, possess significant anti‐inflammatory and antioxidative properties. The efficacy of these NPs in reducing oxidative stress and inflammatory responses was confirmed, leading to a notable reduction in CNV in both experimental and real‐world scenarios. Unlike traditional therapies, CeNPs provide sustained therapeutic effects, reducing the need for frequent administration and potentially improving patient adherence and treatment outcomes. The results indicate that CeNPs represent a promising nanotechnology‐driven therapeutic approach for managing oxidative stress and inflammation in corneal alkali burns, potentially enhancing treatment outcomes and patient prognosis.^[^ ^72^ ^]^

Wang et al. took a different approach by utilizing siRNA‐loaded NPs, specifically nanoparticle‐encapsulated Fidgetin‐like 2 (FL2) siRNA (FL2‐NPsi) that targets the FL2 gene involved in wound healing, for treating chemical injuries caused by corneal alkaline exposure in rat models.^[^ ^73^ ^]^ These NPs, composed of hydrolyzed tetramethyl orthosilicate, encapsulated rat FL2 siRNA or a negative control siRNA.^[^ ^73^ ^]^ FL2 siRNA is directed against the FL2 gene to enhance cell motility and accelerate wound healing; however, its effectiveness may be hindered by challenges related to delivery and cellular uptake due to its large size and negative charge.^[^ ^73^, ^205^ ^]^ By incorporating FL2 siRNA into NPs (FL2‐NPsi), improved delivery, cellular uptake, and bioavailability were achieved with enhanced treatment efficacy in promoting repair of corneal wounds.^[^ ^206^ ^]^ This strategy offers the advantage of precise and efficient delivery of the therapeutic agent to targeted cells, potentially minimizing off‐target effects, and maximizing therapeutic outcomes.^[^ ^207^ ^]^ Therefore, the use of FL2‐NPsi administered via MP technology demonstrates promising effectiveness in facilitating corneal wound repair, suggesting a novel approach to treating corneal injuries. Further investigation and clinical trials are necessary to validate its therapeutic efficacy in human subjects.

Sun et al. synthesized MSNs encapsulating Beva to improve antiangiogenic treatment following corneal alkali burns.^[^ ^73^ ^]^ Beva (Avastin (Ava); Genentech, Inc., South San Francisco, CA, USA), a recombinant humanized monoclonal anti‐VEGF antibody, typically requires frequent administration due to its limited in vivo duration.^[^ ^208^ ^]^ Encapsulating Beva within MSNs extends its residence time in ocular tissues with a more than 70% encapsulation and loading efficiency. MSN‐encapsulated Beva NPs demonstrated no cytotoxicity and showed superior inhibition of VEGF‐induced endothelial cell proliferation, migration, and tube formation compared to free Beva. In vivo, MSN‐Beva exhibited antiangiogenic effects, reducing neovascularization in a corneal alkali burn model.^[^ ^73^ ^]^ This encapsulation method offers a promising approach to extend Beva's therapeutic duration and improve its shelf life for antiangiogenic treatment.

Finally, Ger et al. developed an NP system targeting multiple aspects of corneal injury by incorporating nanoceria (Ce NPs), spermine‐modified hyaluronan (sH), and insulin‐like growth factor 1 (IGF‐1).^[^ ^74^ ^]^ Nanoceria exhibits therapeutic benefits by reducing oxidative stress, inflammation, apoptosis, and angiogenesis;^[^ ^209^ ^]^ hyaluronan functionalized with spermine improves NP permeability across the ocular barrier; and IGF‐1 stimulates wound healing by promoting cell migration and proliferation.^[^ ^74^, ^210^ ^]^ Despite nanoceria's potential, its bioavailability is limited in conventional eye drops.^[^ ^211^ ^]^ Notably, the nanoceria functionalized with spermine‐modified hyaluronan (I@Ce‐hsH) shows promise in treating corneal alkali burns by addressing multiple pathological mechanisms, offering potential for future ocular therapies.

##### Nanocages

Nanocages, a subset of inorganic NPs,^[^ ^212^ ^]^ are characterized by their hollow or porous structure and are typically made from inorganic materials like metals (i.e., gold and silver) or metal oxides.^[^ ^213^ ^]^ Their ability to encapsulate and release therapeutic agents makes them promising candidates for drug delivery.^[^ ^213^ ^]^

**Figure 16:**
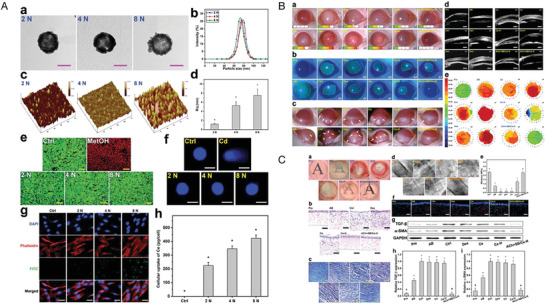
LCNPs with PFD for treating corneal chemical burns. A) Material characterization: a) TEM images, b) DLS spectra, c) surface morphology, d) average roughness; Fluorescence microscopy images: e) live/dead assay, f) comet assay after 2 days of SRCN exposure, g) CLSM images after 4 h of FITC‐SRCN incubation, and h) SRCN cellular uptake efficiency. B) Clinical observations in alkali burn (AB) eye after 8 h and 4 days of treatment with ATS (control), DEX (Dex), and ACh+SB/Ce‐H nano‐formulation: a) slit‐lamp examination, b) corneal fluorescein staining, c) lateral view, d) ultrasound biomicroscopy, and e) corneal topography. C) Tissue analysis at 4 days post‐treatment: a) corneal photos, b) H&E, and c) Masson's trichrome; d) TEM images; e) stromal tissue moduli; f) α‐SMA staining; g) TGF‐β and α‐SMA Western blot analysis; h,i) quantification of TGF‐β and α‐SMA expression. Healthy and AB‐induced groups are included for comparison. Reproduced with permission.^[^ ^75^ ^]^ Copyright 2023, Wiley.

The first use of nanocages in ocular disease treatment was reported by Yang et al. in 2023,^[^ ^75^ ^]^ targeting chemical burns on the ocular surface (**Figure** **16**).^[^ ^75^ ^]^ In their study, ceria nanocages coating poly(l‐histidine) were designed to deliver acetylcholine chloride (ACh) and SB431542, enhancing bioavailability and reducing scar formation.

Ceria nanocages provided antioxidant, anti‐inflammatory, anti‐angiogenic, and anti‐apoptotic effects.^[^ ^75^ ^]^ Traditional administration of ACh and SB431542 is hindered by rapid tear dilution and low bioavailability, requiring frequent dosing. By utilizing nanocages, the study achieved improved corneal penetration, sustained drug release, and pH‐responsive delivery, resulting in enhanced therapeutic efficacy for chemical eye injuries.^[^ ^75^ ^]^

Inorganic NPs, such as SiNPs, MSNs, and CeNPs, have shown significant potential in treating ocular surface injuries, particularly those caused by chemical burns. SiNPs reduce CNV, while MSNs enhance the delivery and efficacy of antiangiogenic therapies like Beva. CeNPs provide sustained anti‐inflammatory and antioxidative effects, addressing oxidative stress and inflammation in corneal injuries. Additionally, novel nanocages, such as ceria nanocages coated with poly(l1‐histidine), offer enhanced drug delivery through controlled release mechanisms, improving therapeutic outcomes. These advancements in NP technology are paving the way for more effective and targeted ocular treatments.

### 3.7 Hydrogels

Hydrogels are water‐based, cross‐linked polymeric networks known for their ability to absorb large amounts of water due to their hydrophilic functional groups.^[^ ^162^ ^]^ They can be synthesized from natural or synthetic polymers and are often engineered for ocular drug delivery to treat various eye conditions. Studies have shown that hydrophilic drugs tend to be released more rapidly from hydrogels.^[^ ^214^ ^]^ Hydrogels form viscoelastic gels through a sol–gel phase transition, which can be triggered by various factors like UV radiation, ion concentration changes, temperature shifts, and pH variations.^[^ ^215^ ^]^

#### 3.7.1 Thermosensitive Hydrogel

Thermosensitive hydrogels have emerged as a promising platform for ocular drug delivery and tissue regeneration due to their unique ability to transition from a liquid to a gel at physiological temperatures.^[^ ^162^, ^208^, ^214^, ^216^ ^]^ This property enables sustained and controlled release of therapeutic agents,^[^ ^215^ ^]^ which is particularly advantageous in treating ocular surface injuries^[^ ^216a^ ^]^ where rapid drug clearance and limited bioavailability are common challenges. Thermosensitive hydrogels not only improve drug retention time and bioavailability but also reduce dosing frequency, enhancing patient compliance. Furthermore, their versatility allows them to be combined with various therapeutic agents, such as anti‐inflammatory drugs, anti‐angiogenic factors, stem cells, and exosomes, to promote tissue repair and regeneration. Recent advancements in nanotechnology have further improved the performance of thermosensitive hydrogels, allowing for enhanced drug stability, targeted delivery, and improved therapeutic outcomes. In this section, we review recent studies that have employed different types of thermosensitive hydrogels for treating ocular chemical injuries, focusing on their composition, mechanism of action, and therapeutic efficacy. The studies are categorized based on the primary material used in the hydrogel, namely cellular/protein‐enhanced, CS‐based, PLGA‐PEG‐PLGA‐based, polysaccharide‐based, and nanocomposite hydrogels.

##### Cellular and Protein‐Enhanced Thermosensitive Hydrogels

**Figure 17:**
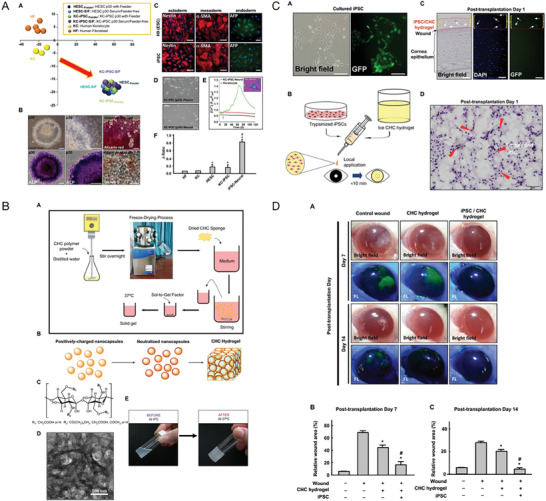
Thermosensitive hydrogel incorporating iPSCs for treating corneal chemical burns. A) Differentiation potential of iPSCs reprogrammed from human corneal keratocytes in a serum‐ and feeder‐free culture system. B) CHC nanoscale hydrogel preparations: A) Preparation steps for CHC nanoscale hydrogels; B) Structure of nanocapsules formed by CHC hydrogel with amphiphatic properties; C) CHC hydrogels' structural formula; D) TEM images showing self‐assembled CHC nanocapsules at higher temperatures; E) Thermosensitive sol‐to‐gel transition in CHC hydrogels. C) Effectiveness of iPSC/CHC hydrogel combination in ex vivo transplantation: A) iPSC monolayer cultured in vivo; B) Application of iPSC/CHC hydrogel on corneal injury; C) Cross‐sectional view showing seamless bio‐scaffold formulation; D) H & E staining revealing nanostructures of CHC capsules with iPSCs. D) Impact of iPSC/CHC hydrogel therapy on corneal wound healing in a chemical injury model. Reproduced with permission.^[^ ^77^ ^]^ Copyright 2012, Elsevier.

This category includes iPSCs‐loaded, exosome‐loaded, and progranulin (PGRN)‐loaded hydrogels. Their name reflects the incorporation of cells (iPSCs), cell‐derived components (exosomes), and regenerative proteins (PGRN), emphasizing their roles in enhancing therapeutic efficacy and promoting corneal regeneration. The combination of cells, cell‐derived components (exosomes), and regenerative proteins with thermosensitive hydrogels can prolong retention time, enhance therapeutic efficacy, and promote corneal regeneration. Chien et al. investigated the use of human corneal keratocyte‐derived induced pluripotent stem cells (iPSCs) combined with a thermo‐responsive injectable nanogel made of carboxymethyl‐hexanoyl CS (CHC) for corneal regeneration (**Figure** **17**).^[^ ^77^ ^]^ While iPSCs alone face challenges with retention in corneal tissue, the CHC nanogel improved delivery and retention; the amphiphilic properties of the CHC nanogel facilitated the sustained presence of iPSCs within a nanogel delivery system. Additionally, using surgical remnants for iPSC generation introduces a sustainable and personalized treatment strategy.^[^ ^77^ ^]^

In another study, an exosome‐loaded nanohydrogel enriched with miRNA 24‐3p significantly enhanced corneal repair, showing potential for acellular therapy.^[^ ^86^ ^]^ Exosomes derived from adipose tissue mesenchymal stem cells were used as nanocarriers to deliver miRNA 24‐3p, which plays a crucial role in enhancing corneal cell migration. The study addressed challenges such as poor penetration and rapid degradation of miRNA 24‐3p when used alone. Exos, composed of proteins, lipids, and miRNAs, improved the stability, availability, and targeted delivery of miRNA 24‐3p, thus extending its therapeutic effects. This Exos‐mediated delivery system proved effective in promoting corneal repair, offering a promising cell‐free therapy for ocular surface injuries.^[^ ^86^ ^]^

A pluronic F‐127 thermosensitive hydrogel carrying PGRN was also shown to promote epithelialization, reduce inflammation, and improve corneal regeneration.^[^ ^89^ ^]^ PGRN, a macromolecule that promotes epithelialization, reduces inflammation, and enhances axonal regeneration, typically faces the challenge of rapid elimination from the ocular surface, requiring frequent application.^[^ ^89^ ^]^ The hydrogel's temperature‐sensitive properties allow for sustained release of PGRN, improving corneal healing, reducing inflammation, and promoting tissue regeneration. This system offers a promising therapeutic option for treating severe corneal injuries.^[^ ^89^ ^]^

##### CS‐Based Thermosensitive Hydrogels

CS‐based hydrogels show good biocompatibility and can deliver anti‐inflammatory drugs or stem cells, promoting corneal repair and reducing neovascularization.

Natural phenolic compounds are well known for their antioxidant properties but face the challenge of low ocular bioavailability when applied topically. Tsai et al. developed a ferulic acid (FA) CS‐based thermosensitive hydrogel that accelerated corneal wound healing through antioxidant and anti‐inflammatory mechanisms.^[^ ^79^ ^]^ The hydrogel demonstrated sustained release, with 28% of FA released over 24 h. Tang et al. developed a gelatin‐CS thermosensitive hydrogel with stromal cell‐derived factor‐1 alpha (SDF‐1 alpha) that significantly improved corneal repair by attracting mesenchymal stem cells (MSCs).^[^ ^81^ ^]^ The hydrogel provided controlled release of recombinant human SDF‐1 alpha (rhSDF‐1 alpha), enhancing its stability and bioactivity, which promoted the regeneration of corneal epithelium.^[^ ^81^ ^]^

An indomethacin (IND)‐loaded poloxamer‐hyaluronic acid (HA)‐CS hydrogel has also been developed, offering sustained drug release, and promoting the healing of corneal burns.^[^ ^88^ ^]^ The use of poloxamer enabled gelation at physiological temperature, while HA provided mucoadhesive properties and hydration.^[^ ^217^ ^]^ IND, an NSAID with higher potency and lower toxicity compared to other NSAIDs, aimed to reduce inflammation.^[^ ^218^, ^219^ ^]^ However, IND's low water solubility and alkaline instability posed challenges for topical use.^[^ ^220^ ^]^ The hydrogel formed nanomicelles at room temperature and transitioned to a gel at body temperature, displaying sustained drug release, biological compatibility, and improved corneal wound healing compared to commercial treatments. This system holds promise for enhancing ocular burn management by improving drug delivery and promoting wound recovery.^[^ ^88^ ^]^ Further research is needed to fully understand the molecular mechanisms behind its therapeutic effects.

Wang et al. developed a 4D‐printed CS‐based thermosensitive hydrogel (4D‐CTH) loaded with limbal epithelium stem cells (LESCs) that enhanced corneal epithelial regeneration and repair.^[^ ^90^ ^]^ The 4D‐CTH encapsulates LESCs, improving their delivery, retention, and viability at the injury site, and thus promoting corneal epithelium repair.^[^ ^221^ ^]^ By incorporating CS, the hydrogel provides a stable platform for stem cell therapy, supporting cell attachment, proliferation, and differentiation.^[^ ^222^ ^]^ Although challenges such as biocompatibility and cost remain, 4D‐CTH's adjustable structure and uniform pore size significantly enhance stem cell delivery and wound repair, offering a promising therapeutic approach.^[^ ^223^ ^]^ Overall, this research demonstrates the potential of 4D‐CTH for improving stem cell transplantation efficiency and accelerating corneal injury repair.^[^ ^90^ ^]^

##### PLGA‐PEG‐PLGA‐Based Thermosensitive Hydrogels

PLGA‐PEG‐PLGA hydrogels are commonly used for long‐term drug release, demonstrating good biocompatibility and drug‐loading capacity. They significantly reduce the frequency of drug administration, making them suitable for treating ocular surface chemical injuries.

**Figure 18:**
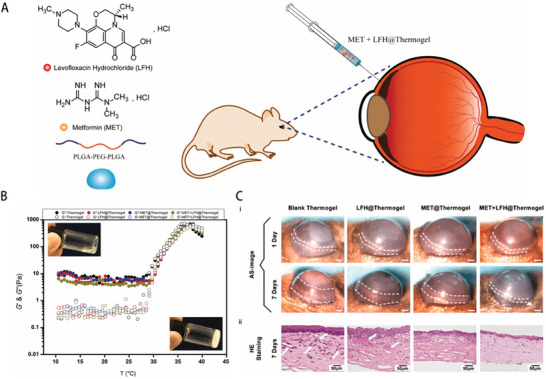
An example hydrogel–drug system involving MET + LFH@Thermogel. A) Diagram illustrating the administration of ME + LFH@Thermogel via SCJ injection. B) Drug‐loaded polymer/water system displaying temperature‐dependent fluctuations in storage (G') and loss (G") moduli. At a concentration of 3 mg mL^−1^ for both MET and LFH, the accompanying visuals depict the existence of MET + LFH@Thermogel in a sol state at room temperature (25 °C), transitioning to a gel state at body temperature (37 °C). C) Effectiveness of various treatments in combating CNV using an alkali‐burn‐induced corneal model; i) Illustrative photographs depict the therapeutic impact on corneas at 1 and 7 days across various treatment groups; ii) Representative histological images stained with H&E from different treatment groups at the 7‐day mark after corneal alkali burn. Reproduced with permission.^[^ ^82^ ^]^ Copyright 2019, Drug Delivery.

In one study, a PLGA‐PEG‐PLGA hydrogel was used to treat CNV caused by alkali burns, with drug release lasting up to one month.^[^ ^82^ ^]^ Liu et al. developed a continuous delivery mechanism for Metformin (MET) and levofloxacin hydrochloride (LFH) using a thermosensitive PLGA‐PEG‐PLGA hydrogel administered via SCJ injection (**Figure** **18**).^[^ ^82^ ^]^ Upon reaching body temperature, the drug‐loaded hydrogel transitioned from liquid to gel, enabling controlled drug release. In vitro tests confirmed low toxicity and good biocompatibility, with drug release lasting up to one month.^[^ ^82^ ^]^ In vivo, the combined MET+LFH treatment significantly reduced neovascularization and suppressed inflammatory and angiogenic cytokines, demonstrating the system's potential for effectively managing ocular neovascularization.

Challenges in delivering ophthalmic drugs arise not only from inadequate solubility of hydrophobic treatments but also from the unique architecture and physiology of the eye, which lead to limited permeability and rapid drug elimination. In one study, a PLGA‐PEG‐PLGA hydrogel loaded with DEX effectively treated choroidal neovascularization following alkali burns, significantly improving drug efficacy.^[^ ^83^ ^]^ PLGA‐PEG‐PLGA, known for its biocompatibility, increased DEX solubility by 5.2 times, forming a gel at body temperature.^[^ ^83^ ^]^ The thermogel provided sustained drug release in vitro and, in a rat model, significantly improved healing and reduced neovascularization. This in situ gelling system offers a promising approach for prolonged ophthalmic drug delivery.

A PLGA‐PEG‐PLGA hydrogel combined with TNF‐α and VEGF inhibitors was also found to provide long‐term anti‐inflammatory and anti‐neovascular effects, promoting wound healing on the ocular surface.^[^ ^87^ ^]^ The study utilized adalimumab, a TNF‐α inhibitor that reduces inflammation, and aflibercept, a VEGF inhibitor that prevents angiogenesis. Using either inhibitor alone proved less effective in addressing both inflammation and neovascularization. The system employed biodegradable PLGA‐PEG‐PLGA hydrogel, which facilitated the sustained release of therapeutic antibodies over three months. This prolonged, controlled release improved bioavailability while minimizing systemic exposure. The combined inhibition of TNF‐α and VEGF effectively prevented CNV and protected retinal and optic nerve tissues, offering a promising treatment approach for severe ocular injuries.^[^ ^87^ ^]^

##### Polysaccharide‐Based Thermosensitive Hydrogels

Polysaccharide‐based hydrogels exhibit good biocompatibility and tunable gelation properties, showing potential in nano drug‐delivery systems and stem cell therapies. In one case, a polysaccharide‐based hydrogel combined with MSCs, which also falls under the category of cellular‐enhanced thermosensitive hydrogels, was found to promote tissue regeneration in ocular surface alkali burns.^[^ ^78^ ^]^ The polysaccharide hydrogel served as a scaffold, improving MSC survival, integration, and therapeutic effectiveness. It also controlled growth factor release, maintained a healing environment, and enhanced tissue regeneration.^[^ ^78^ ^]^ As previously before, a 4D‐printed polysaccharide‐based CS hydrogel has also been used to deliver limbal epithelial stem cells (LSCs), significantly enhancing corneal epithelial repair.^[^ ^90^ ^]^

##### Nanocomposite Thermosensitive Hydrogels

A doxycycline temperature‐sensitive hydrogel (DTSH) was used to improve CNV and wound healing by prolonging drug retention and stability.^[^ ^76^ ^]^ DTSH is composed of doxycycline, hydroxypropyl‐β‐cyclodextrin, poloxamer 407, and poloxamer 188 in a mass ratio of 1:24:220:35, respectively.^[^ ^76^ ^]^ Doxycycline inhibits MMPs, VEGF, and inflammatory factors like iNOS and IL‐1β, while Beva directly targets VEGF to reduce neovascularization. The controlled, sustained release of doxycycline through DTSH provided enhanced stability and bioavailability, offering synergistic treatment for CNV and promoting corneal healing more effectively than topical Beva or DTSH alone, presenting a promising therapeutic approach.^[^ ^76^ ^]^

A suramin (SUR) and Beva‐loaded nanocomposite hydrogel effectively inhibited CNV. Quinteros et al. used SUR and Beva in a poloxamer hydrogel to extend antiangiogenic effects, leading to improved histological outcomes in CNV.^[^ ^80^ ^]^ SUR, an anti‐parasitic with antiangiogenic properties, and Beva, a VEGF inhibitor, were combined to extend their therapeutic effects given their respective rapid clearance limit and short half‐life when used alone.^[^ ^80^ ^]^ The hydrogel, made with poloxamer 407, undergoes gelation at body temperature, enabling controlled drug release and prolonged retention in corneal tissue. This approach reduced neovascularization and improved histological outcomes compared to liquid drug forms.^[^ ^80^ ^]^

In another study, a nintedanib‐loaded nano‐thermosensitive hydrogel (NNTH) gradually released the drug at 37 °C, significantly reducing CNV.^[^ ^85^ ^]^ Liu et al. developed a nano‐formulation of nintedanib to enhance its bioavailability and maintain its concentration in eye drops and gels.^[^ ^85^ ^]^ Their study evaluated different ophthalmic formulations of nintedanib, including a water‐based solution, nintedanib nano‐thermo‐reversible hydrogel (NNTH), and DEX ointment in a rat model of corneal alkali burns.^[^ ^85^ ^]^ The thermo‐reversible hydrogel remained a liquid at 16 °C but solidified at 37 °C, releasing the drug more gradually compared to free nintedanib (FN). Over 24 h, NNTH released only 53.2% ± 6.25% of the drug, demonstrating a slower, sustained release. This controlled release helped reduce ocular neovascularization and could improve patient compliance and clinical outcomes. Thus, NNTH shows promise as a more convenient and effective therapeutic option for treating neovascularization.

A nintedanib‐loaded thermo‐sensitive hydrogel (NTH) also demonstrated sustained drug release at 37 °C, inhibiting neovascularization and reducing inflammatory cytokine expression.^[^ ^84^ ^]^ Nintedanib, a tyrosine kinase inhibitor targeting VEGFR2, has shown potential in reducing choroidal neovascularization.^[^ ^224^ ^]^ However, its rapid clearance limits its long‐term efficacy. The hydrogel is composed of nintedanib, poloxamer 407, and an artificial tear solution containing sodium chloride, sodium bicarbonate, potassium chloride, and calcium chloride dihydrate.^[^ ^84^ ^]^ The thermo‐sensitive hydrogel addresses this by undergoing gelation at body temperature (37 °C), enabling sustained drug release.^[^ ^225^ ^]^ This study demonstrated that NTH reduced neovascularization progression, decreased the affected area, and lowered CD31 and VEGFR2 expression levels.^[^ ^84^ ^]^ This approach highlights the potential of nano‐thermosensitive hydrogels in improving drug delivery and clinical outcomes for ocular injuries. ^[^ ^226^ ^]^

In conclusion, thermosensitive hydrogels, particularly those enhanced with cells or proteins, as well as those based on CS, PLGA‐PEG‐PLGA, polysaccharides, and nanocomposite, offer versatile platforms for sustained drug release and tissue repair in ocular surface treatments. Cellular and protein‐enhanced hydrogels improve regenerative therapies by enhancing cell delivery and therapeutic efficacy. CS‐based hydrogels excel in biocompatibility and are effective when combined with anti‐inflammatory drugs or stem cells. PLGA‐PEG‐PLGA hydrogels, with their extended drug release capabilities, are ideal for reducing dosing frequency in complex ocular conditions. Polysaccharide‐based hydrogels, especially when integrated with emerging technologies like 4D printing, have shown promise in promoting tissue regeneration. The incorporation of nanotechnology into nanocomposite hydrogels enhances drug stability and prolongs therapeutic effects, making them effective in managing inflammation and neovascularization. These diverse hydrogel systems, in combination with nanotechnology, are pushing the boundaries of ocular surface injury treatment, providing advanced solutions to long‐standing challenges.

#### 3.7.2 In Situ Hydrogel

In situ hydrogels are those administered initially in a liquid or semi‐liquid state, following which they undergo gelation at the designated site, thereby resulting in gel formation. The exploration of these materials for treating ocular surface chemical burns began around 2010. Anumolu and colleague developed doxycycline‐loaded PEG hydrogels to heal vesicant‐induced ocular wounds in rabbit corneas.^[^ ^91^ ^]^ The PEG‐based hydrogel transitions from liquid to gel at physiological pH, ensuring prolonged contact with the corneal surface and providing sustained doxycycline release for up to 7 days. The formulation enhanced drug effectiveness through prolonged contact time and improved mechanical resistance, leading to better wound healing than doxycycline in solution. Overall, the study concluded that PEG‐based doxycycline hydrogels significantly improve corneal healing after vesicant injuries, presenting an effective therapeutic option for ocular mustard injuries.^[^ ^91^ ^]^

Expanding the application of hydrogel, Zhang and colleagues investigated the topical efficacy of Gly‐thymosin β_4_ (Gly‐Tβ_4_) in treating rabbit corneal alkali burns, finding that Gly‐Tβ_4_ solutions yielded better corneal recovery than hydrogel formulations.^[^ ^92^ ^]^ Gly‐Tβ_4_, a biomimetic variant of natural thymosin β_4_, promotes tissue healing and regeneration after alkali burns;^[^ ^227^ ^]^ however, hydrogel forms of Gly‐Tβ4 may cause irritation and slow drug release, impacting therapeutic efficacy.^[^ ^92^ ^]^ This in situ hydrogel formulation was composed of Gly‐Tβ_4_, poloxamers 188 and 407, and phosphate‐buffered solution, with purified water as the solvent. Notably, direct contact with the hydrogel may irritate burned corneas and impede the rapid release of Gly‐Tβ_4_. The study also showed that Gly‐Tβ_4_ solutions accelerated corneal healing more effectively than hydrogel compositions due to reduced irritation and faster drug release. As this was a preliminary investigation, the hydrogel formulation did not outperform the solution, indicating a need for further refinement of the hydrogel design.^[^ ^92^ ^]^

Currently, there is no ideal framework for the implanting LSCs to assist in corneal regeneration after corneal alkali burns.^[^ ^93^ ^]^ To address this, Xu et al. developed an alginate–CS hydrogel (ACH) for LSC transplantation.^[^ ^93^ ^]^ This hydrogel forms through the self‐crosslinking of carboxymethyl CS (CMCTS) and sodium alginate (SAD) via Schiff's base reactions, eliminating the need for additional chemical crosslinking agents. The in situ hydrogel exhibits excellent transparency, significant swelling capacity, biocompatibility, and compatibility with LSCs, making it suitable for tissue engineering. As such, this in situ hydrogel presents a promising scaffold for LSC transplantation with rapid tissue regeneration and wound‐healing properties, offering an effective strategy for repairing corneal injuries from alkali burns and other types of severe ocular damage.

Bioadhesive hydrogel could serve as an effective carrier for the sustained release of various therapeutics. Xu et al. developed a cysteine (Cys)‐modified thiolated γ‐polyglutamic acid (PGA‐Cys) hydrogel, while the thiol groups could bond with the mucin layer of the cornea to give bioadhesion and sustained release of keratinocyte growth factor (KGF) to improve corneal repair.^[^ ^94^ ^]^ KGF promotes the proliferation and differentiation of epithelial cells, aids in wound healing, safeguards cells, and prevents apoptosis.^[^ ^94^ ^]^ This study revealed that PGA‐Cys hydrogel significantly enhances the reparative effects of KGF on damaged corneas by improving bioadhesion and ensuring sustained release.^[^ ^94^ ^]^

**Figure 19:**
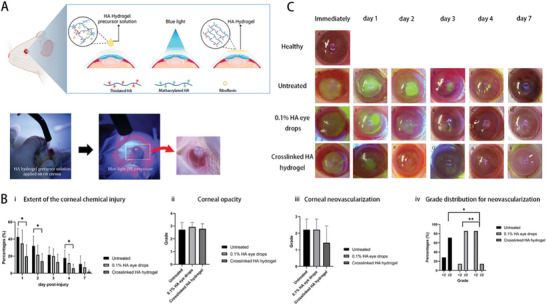
An example drug‐delivery system utilizing in situ hydrogel formation incorporating medications into HA hydrogels. These hydrogels undergo crosslinking triggered by blue light through a thiol‐ene reaction. A) Illustration presenting the creation of a HA hydrogel by means of a thiol‐ene reaction activated by blue light. This chemical process entails the interaction between thiol‐modified HA and methacrylated HA in the presence of riboflavin phosphate. The visuals demonstrate how the thiol‐ene reaction, triggered by blue light, produces the on‐site HA hydrogel formation on the corneal surface of a rat. B, i–iv) Quantitative outcomes from both the injured and treated groups, encompassing measurements of the corneal chemical burn severity (i), corneal opacity (ii), CNV (iii), and grade distribution for neovascularization (iv). C) Illustrative visuals portraying corneal conditions following various treatments across four groups over six distinct time intervals. The four groups comprise a healthy cornea (A), an untreated group (B–G), a group treated with 0.1% HA eye drops (H–M), and a crosslinked HA hydrogel‐treated group (N–S). The six time points correspond to immediately post‐injury as well as days 1, 2, 3, 4, and 7 following the injury. Reproduced with permission.^[^ ^96^ ^]^ Copyright 2022, Elsevier.

Studies have demonstrated the anti‐angiogenic and anti‐inflammatory properties of MSCs as well as their secreted factors, known as the secretome. Nevertheless, applying MSCs topically or utilizing their secretome encounters obstacles concerning delivery and swift elimination. Na and co‐researchers found that a four‐arm PEG‐N‐hydroxysuccinimide and collagen hydrogels could act as effective encapsulation carriers for MSCs, allowing a consistent stay of MSCs to the corneal surface.^[^ ^95^ ^]^ This approach significantly accelerated the healing of epithelial wounds and decreased corneal scarring compared to untreated controls.^[^ ^95^ ^]^ HA, a glycosaminoglycan characterized by a substantial molecular mass and is also a key component of the extracellular matrix, demonstrates notable viscosity^[^ ^228^ ^]^ and enhances tear‐film stability, reducing drainage from the ocular surface.^[^ ^229^ ^]^ Approximately 90% of applied HA is washed out within an hour of administration.^[^ ^230^ ^]^ Park and colleagues evaluated the therapeutic benefits of one in situ HA hydrogel triggered by blue light in a rat model with alkali‐induced corneal injury (**Figure** **19**).^[^ ^96^ ^]^ They observed enhanced corneal re‐epithelialization on the first, second, fourth, and seventh days following injury. Moreover, decreased CNV and a more organized collagen configuration were obtained. Interestingly, the administration of 0.1% HA eye drops effectively lowered the expression levels of α‐SMA, MMP9, and IL1‐β, indicating an anti‐inflammatory effect of the hydrogel itself. HA‐based in situ hydrogels could endow the sustained stay as a carrier and provide synergistic therapeutic effects through re‐epithelization and anti‐inflammatory properties.^[^ ^96^ ^]^

In situ hydrogels represent a significant advancement in the treatment of ocular surface chemical burns and other corneal injuries due to the special location of eyes. Biocompatibility is a primary concern, and FDA‐approved materials like PEG are expected to significantly accelerate clinical translation. Increasing bioadhesiveness will improve the duration of contact with the ocular surface, and so developing ways to increase adhesion will be a highly important avenue of future research.

#### 3.7.3 Other Hydrogels

Corneal damage, resulting from diseases or injuries, ranks as the second leading cause of global blindness after cataracts.^[^ ^231^ ^]^ Treatment typically involves corneal transplants, with full‐thickness PKP being the standard approach, although partial‐thickness lamellar keratoplasty may be an option if the endothelium is intact, showing high success rates.^[^ ^232^ ^]^ A major challenge in many countries is the shortage of quality donor tissue.^[^ ^106^ ^]^ Additionally, outcomes are often unsatisfactory in high‐risk cases, where the recipient's corneal bed is infected or neovascularized, leading to higher graft failure rates.^[^ ^233^ ^]^

In response to these challenges, innovative hydrogel‐based strategies have been developed for ocular surface repair. Yang and colleagues introduced a chemically‐modified, cross‐linked hyaluronan derivative (CMHA‐SX) designed to accelerate re‐epithelialization, improve tissue thickness, and enhance tissue organization in rabbit models of corneal epithelial abrasions and alkali burns.^[^ ^97^ ^]^ Hackett et al. further explored recombinant human collagen‐phosphorylcholine (RHCIII‐MPC) hydrogels for treating severe corneal injuries.^[^ ^98^ ^]^ In their study involving rabbit models with alkali burns, all implants achieved full epithelial coverage post‐surgery. Notably, RHCIII‐MPC implants resisted vascular ingrowth and promoted endogenous cell and nerve repopulation.

In pursuit of novel keratoprostheses, in 1998, Hicks et al. evaluated poly (2‐hydroxyethyl methacrylate) (PHEMA) for restoring corneal integrity and visual function in a rabbit alkali burn model.^[^ ^234^ ^]^ Their findings suggested that PHEMA keratoprostheses effectively promote corneal healing and improve visual outcomes in rabbits with alkali‐induced corneal injuries, demonstrating the potential of this approach in treating severe corneal damage in humans.^[^ ^234^ ^]^ Xiang et al. later refined this approach, developing a T‐style PHEMA keratoprosthesis with enhanced mechanical durability and improved integration with host tissues through surface modifications.^[^ ^99^ ^]^ In this case, the surface of the hydrogel was tailored with HA and cationized gelatin to enhance cell adhesion and biointegration.^[^ ^99^ ^]^

To combat CNV, Huang et al. developed a hydrogel combining MPEG‐PCL micelles with α‐cyclodextrin through a “host–guest” interaction to co‐deliver DEX sodium phosphate (Dexp) and Ava.^[^ ^100^ ^]^ This formulation demonstrated prolonged retention, excellent biocompatibility, and effective inhibition of CNV by reducing key inflammatory markers in vitro and in vivo. Zhou et al. devised a polymer‐based delivery system for infliximab, designed to address the limitations of systemic administration and improve ocular bioavailability. The sustained release of infliximab forms a porous polydimethylsiloxane scaffold, showing promise for reducing inflammation and neovascularization.^[^ ^101^ ^]^

Luo et al. introduced cryogels composed of gelatin and ascorbic acid (AA) for corneal stromal tissue engineering.^[^ ^102^ ^]^ By mixing gelatin with varying levels of AA and then cross‐linking with carbodiimide, the cryogel was successfully formed. This combination stimulated keratocyte growth while maintaining tissue transparency and promoting regeneration in an alkali burn model.^[^ ^102^ ^]^ Similarly, Chen and colleagues developed a gelatin methacryloyl (GelMA) hydrogel eye patch incorporating amniotic extract (AE) to inhibit symblepharon formation following ocular alkali injuries, showing enhanced epithelial healing and reduced inflammation.^[^ ^103^ ^]^ GelMA promoted cell adhesion, migration, proliferation, and wound healing, while the AE prevented viral transmission, reduced inflammation, promoted repair, and reduced scarring.^[^ ^103^ ^]^ GelMA is modified with methacrylic anhydride substitution through photo‐crosslinking and slowly releases the loaded AE during degradation, efficiently inhibiting symblepharon formation in rabbits through the facilitation of epithelial healing, mitigation of inflammation, and provision of a durable framework for tissue regeneration.^[^ ^103^ ^]^

Further advancements include the development of synthetic corneal implants by Simpson et al., whose collagen‐like peptide‐polyethylene glycol (CLP‐PEG) hydrogels demonstrated effective nerve regeneration and inhibition of dendritic cell activation in mini‐pig alkali burn models.^[^ ^104^ ^]^ Synthetic corneal implants made from collagen‐like peptide‐polyethylene glycol (CLP‐PEG) and the anti‐inflammatory polymer, 2‐methacryloyloxyethyl phosphorylcholine (MPC), showed promising results;^[^ ^104^ ^]^ CLP‐PEG, stabilized by DMTMM, provides structural support, while MPC reduces inflammation, edema, opacity, and neovascularization, promoting nerve regeneration and corneal sensitivity recovery. While MPC alone is insufficient, its combination with the CLP‐PEG scaffold enhances tissue regeneration and mechanical stability.^[^ ^104^ ^]^ The hydrogels combining CLP‐PEG and MPC successfully promoted epithelial cell growth, inhibited dendritic cell activation, and reduced inflammation, making them a promising alternative to human donor corneas for corneal regeneration and inflammation control.^[^ ^104^ ^]^ Huang et al. also designed a monolith‐hydrogel composite for sustained triamcinolone acetonide (TA) delivery, effectively controlling inflammation and inhibiting CNV.^[^ ^105^ ^]^ TA, the active agent, reduces inflammation and induces vasoconstriction. The composite structure consists of an organic molecular cage with methacrylate groups and acetylated gelatin, forming a porous framework for sustained drug release.^[^ ^105^ ^]^ The acetylated gelatin hydrogel enhances TA loading and release, improving biocompatibility and reducing tissue swelling. This study highlights the potential of these composites for controlled TA delivery for inhibiting CNV, with broader biomedical applications.^[^ ^105^ ^]^

**Figure 20:**
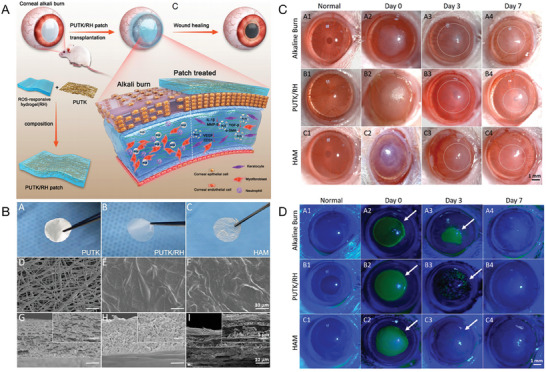
An example drug‐delivery system utilizing a ROS‐scavenging hydrogel (RH) incorporating PUTK into a PUTK/RH patch to promote corneal healing after chemical burns. A) Diagrammatic representation of a RH/PUTK patch and its use in vivo for treating alkali burns in the cornea. When employed as a wound patch and material for scavenging ROS, implanted the patch on rat corneas injured by alkali burns accelerates epithelial regeneration; reduces inflammation in the stroma; and decreases the expression of inflammation‐related genes (IL‐1β and MMP‐9), angiogenesis (VEGF and CD‐31), and fibrotic scarring (TGF‐β and α‐SMA), thereby enhancing corneal transparency recovery. B) Morphologies and microstructural characteristics of the PUTK membrane, PUTK/RH patch, and HAM; Digital images (A–C) and Scanning electron microscopy images depicting surface features (D–F) and cross‐sectional views (G–I). Insets show magnified views. C) Observation using slit‐lamp microscopy of rat corneas, following alkali burns, transplanted with PUTK/RH patches and HAM. White circles outline the wound bed post‐transplantation. D) Photographs (A–C) of rat corneas transplanted with HAM and PUTK/RH patches after alkali burn, stained with fluorescein. Reproduced with permission.^[^ ^106^ ^]^ Copyright 2023, Elsevier.

Oxidative stress, driven by ROS, is a key factor in the pathology of ocular alkali injuries.^[^ ^235^ ^]^ The oxidized form of deoxyguanosine, 8‐oxo‐2‐deoxyguanosine (8‐oxo‐dG), indicates oxidative damage and has shown anti‐inflammatory effects similar to corticosteroids in ocular alkali burn models.^[^ ^236^ ^]^ However, its short ocular retention time limits efficacy, requiring frequent high‐concentration applications that may reduce patient compliance and increase the risk of systemic toxicity.^[^ ^237^ ^]^ Shi et al. addressed this by developing a dual‐network biomaterial combining thioketal‐infused polyurethane (PUTK) nanofibers with a crosslinked polymer of poly (ethylene glycol) methyl ether methacrylate, glycidyl methacrylate, thioketal diamine, and 3,3′‐dithiobis(propionohydrazide) to promote epithelial regeneration and reduce scarring in alkali‐burned corneas (**Figure** **20**).^[^ ^106^ ^]^ Their PUTK/RH patch showed excellent transparency, tensile strength, water affinity, and antioxidant activity against both 2,2‐di(4‐*tert*‐octylphenyl)‐1‐picryl‐hydrazyl (DPPH) and H_2_O_2_. In rat models, the PUTK/RH patch reduced inflammation; downregulated genes related to inflammation, neovascularization, and scarring; and promoted corneal healing with less scarring. These results demonstrate the potential of the PUTK/RH patch for treating ocular chemical injuries.^[^ ^106^ ^]^

Long et al. developed an injectable hydrogel composed of HA, tannic acid, and 2‐formylbenzeneboronic acid (FPBA) integrated with STB‐loaded polymer NPs for treating CNV.^[^ ^107^ ^]^ The key components are STB, which inhibits angiogenesis, and tannic acid, which provides antioxidant properties, and the FPBA enables their crosslinking, forming the hydrogel structure. Polymer NPs made from polylactic‐co‐glycolic acid (PLGA) were used to encapsulate STB, allowing for controlled drug release and improved ocular bioavailability.^[^ ^107^ ^]^ This system ensured a sustained release of STB, enhancing its anti‐angiogenic effects in vitro and in vivo, offering a promising therapeutic approach for CNV and associated oxidative stress.^[^ ^107^ ^]^ Zhang et al. developed a composite hydrogel loaded with deoxyribonuclease I (DNase I) to regulate neutrophil extracellular traps (NETs) and inhibit neovascularization after corneal alkali burns.^[^ ^108^ ^]^ The hydrogel consists of CS NPs loaded with DNase I and synthesized methacryloylated filipin proteins, which form a gel upon light exposure. DNase I disrupts NETs to reduce inflammation and neovascularization, but repeated administration is required for effectiveness, increasing the risk of infection and complicating adhesion to the corneal surface. The hydrogel provides sustained release of DNase I, allowing localized, continuous treatment to regulate NETs and inhibit neovascularization. This approach shows promise for improving visual outcomes and reducing complications from corneal chemical injuries.^[^ ^108^ ^]^

Lastly, Wang et al. devised a novel ocular delivery system, Imatibib (IMB)@glycymicelle‐hydrogel, for treating corneal alkali burns.^[^ ^109^ ^]^ IMB, the active ingredient, was encapsulated in glycyrrhizin‐containing glycymicelles to improve solubility and target CNV and inflammation.^[^ ^238^ ^]^ Due to IMB's poor solubility, the formulation incorporated hydroxypropyl methylcellulose (HPMC) and sodium hyaluronate to extend retention on the eye and provide sustained drug release. The system significantly accelerated corneal healing, restored corneal sensitivity, reduced opacity, and inhibited CNV.^[^ ^109^ ^]^ Na et al. explored the combined use of epidermal growth factor (EGF) and KGF within a HA hydrogel to improve corneal wound healing.^[^ ^110^ ^]^ EGF supports epithelial regeneration, while KGF aids in stromal layer regeneration, with their combined effects promoting efficient healing of both corneal layers. Using HA as the hydrogel base allows for the controlled, sustained release of these growth factors at the wound site, overcoming the limitations of rapid clearance and frequent dosing associated with conventional treatments. This approach enhances therapeutic efficacy and offers a promising strategy for corneal wound management.^[^ ^110^ ^]^

In summary, the development and evaluation of various hydrogel systems represents a promising advancement in the management of corneal injuries and diseases. Each approach offers unique advantages, from enhancing healing through chemical modifications and cross‐linking to incorporating advanced drug‐delivery systems and biomaterials. These innovations address critical challenges such as poor donor tissue availability, high‐risk graft scenarios, and CNV. By integrating novel materials, controlled drug release mechanisms, and targeted therapeutic agents, these studies highlight significant progress toward more effective treatments for corneal damage and related ocular conditions. The continued exploration of these technologies holds potential for improving visual outcomes and expanding therapeutic options for patients worldwide.

### 3.8 Dendrimers

Dendrimers exhibit a precisely defined structure, resembling highly branched, tree‐like synthetic macromolecules. They feature a core at the center, a repeating unit interior, and an exterior adorned with multiple functional end groups.^[^ ^239^ ^]^ Their unique architecture allows for high uniformity, multivalency, and the capacity to carry diverse molecules, making them ideal for drug delivery, diagnostics, and nanotechnology applications.^[^ ^240^ ^]^ Dendrimers, with their customizable surface functionalities, nanoscale dimensions, and solubility in water, have the capability to improve the stability, solubility, and bioavailability of medicinal substances. This confers notable benefits in the realm of targeted and prolonged delivery mechanisms.^[^ ^241^ ^]^ There is a scarcity of research on utilizing dendrimer materials for treating corneas damaged by alkali burns—only a single study was found during our investigation.

**Figure 21:**
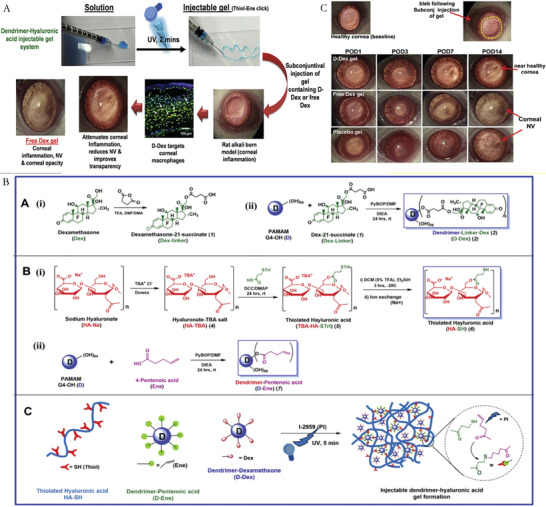
An example drug‐delivery system utilizing G4‐PAMAM dendrimers incorporating DEX into dendrimer‐DEX (D‐Dex) for extended application and enhanced treatment of inflammation in the cornea after chemical burns. A) Graphical abstract. B) Preparation of dendrimer conjugates and injectable gel components: A) Synthesis of (D‐Dex) conjugates; B) Synthesis of injectable gel components (i) thiolated hyaluronic acid (HA‐SH), and production of HA modified with thiol groups (ii) dendrimer‐pentenoic acid (D‐Ene) conjugates. C) Schematic representation of the formation of an injectable gel loaded with D‐Dex through thiol‐ene click chemistry. C) Clinical efficacy of subconjunctival D‐Dex and Free DEX gel for corneal opacity and neovascularization. Reproduced with permission.^[^ ^111^ ^]^ Copyright 2017, Elsevier.

Soiberman et al. developed a dendrimer‐DEX (D‐Dex) gel for sustained SCJ injection to manage corneal inflammation (**Figure** **21**).^[^ ^111^ ^]^ G4‐PAMAM dendrimers were chosen as nanocarriers to improve the solubility, bioavailability, and targeted delivery to inflamed corneal macrophages of DEX. The system effectively reduced inflammation compared to free DEX, accompanied by minimized side effects including increased IOP and corneal toxicity. The dendrimer‐based delivery systems could supply sustained drug release, further lowering the required administration frequency, which gives a promising, targeted treatment choice for corneal inflammation with enhanced efficacy and reduced side effects.^[^ ^111^ ^]^ Future research could explore the broader applications of dendrimers in ocular therapies and other treatments.

### 3.9 Nanocomplex

**Figure 22:**
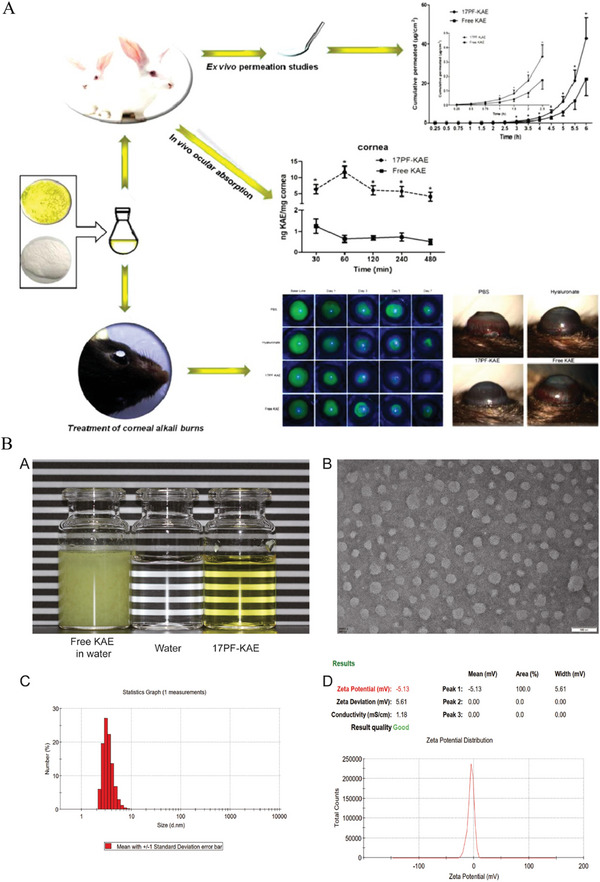
An example nanocomplex–drug system based on the 17PF‐KAE nanocomplex. A) Graphical abstract of the PVP‐17PF and KAE complexing‐based ocular therapeutic formulation. Ex vivo permeation experiments for 17PF‐KAE and the unformulated KAE solution are illustrated, showing the cumulative time of permeation and their corresponding permeation profiles. The capacity for ocular absorption in vivo was evaluated by quantifying concentrations of KAE in rabbit eye tissues following the administration of four doses (50 µL per dose given at 10‐min intervals) (*p < 0.05 in comparison to a KAE solution of 4.5 mg mL^−1^ without formulation; *n* = 6). Cornea fluorescein staining was used to represent the recovery of corneal injury after alkali burns. Slit‐lamp photo shows the area of CNV on day 7 after injury under different treatments. B) Characterization of the ophthalmic solution 17PF‐KAE, which has a 17PF weight ratio of 18:1: A) Visual appearance of the solution; B) Complex morphology observed via TEM at a magnification of ×50000 (scale bar = 100 nm); C) Size distribution of the nanocomplexes; D) Zeta potential measurement of the 17PF‐KAE ophthalmic solution. Reproduced with permission.^[^ ^112^ ^]^ Copyright 2020, Elsevier.

Building on advancements in ocular drug delivery, Zhang et al. introduced a novel ocular therapeutic using the complexation of polyvinylpyrrolidone (PVP), K‐17PF (17PF), and Kaempferol (KAE) to enhance the solubility of KAE, a compound with limited aqueous solubility (**Figure** **22**).^[^ ^112^ ^]^ This formulation, prepared via a simple thin‐film hydration method, transformed KAE into a transparent ophthalmic solution with excellent dispersion in aqueous environments.^[^ ^242^ ^]^ The 17PF‐KAE complex showed high tolerance, improved permeability across ocular barriers, enhanced antioxidative and anti‐inflammatory effects, and showed superior efficacy in treating corneal alkali burns. This approach offers a promising eye‐drop formulation for managing oxidative stress and inflammation, particularly in corneal chemical injuries.^[^ ^112^ ^]^

In another innovative development, Zhou et al. introduced a multifunctional delivery system using self‐assembling tetrahedral DNA nanostructures (TDNs) for targeted drug delivery and treatment.^[^ ^113^ ^]^ TDNs were loaded with therapeutic agents such as antisense peptide nucleic acid (asPNA), DNA aptamers, and small‐molecule drugs like paclitaxel and wogonin. Enhanced drug stability, improved targeting, and reduced cytotoxicity were achieved by introducing the delivery systems. In vivo studies demonstrated that TDNs enhanced drug accumulation at corneal alkali burn sites, significantly reducing inflammation and accelerating healing, making it a promising strategy for treating corneal injuries.^[^ ^113^ ^]^

In another development, Yin et al. explored therapeutic contact lenses incorporating KAE into bovine serum albumin (BSA) fibrous films to treat corneal chemical injuries.^[^ ^114^ ^]^ The BSA/KAE films are highly transparent, water‐rich, and enable sustained drug release, addressing KAE's limitations of low water solubility and short retention times on the ocular surface. KAE, known for its antioxidant and anti‐inflammatory properties, aids in reducing inflammation and inhibiting neovascularization. The inclusion of BSA nanomaterials improves bioavailability, prolongs retention, and enhances therapeutic efficacy, making it an option for corneal wound treatment.^[^ ^114^ ^]^

Lastly, Cao et al. developed a pH‐responsive polymer carrier, TPPA, to deliver siRNA targeting VEGFA to inhibit CNV.^[^ ^115^ ^]^ While siVEGFA silences VEGFA expression, its delivery faces challenges including poor membrane permeability and degradation. Using the pH‐responsive nature of TPPA aids siRNA release within cells. This study demonstrates that TPPA has potential as an effective siRNA carrier, offering an approach for CNV treatment and providing insights into the design of polymers for anti‐angiogenesis therapies.^[^ ^115^ ^]^

Recent research on nanocomplexes highlights significant advancements in ocular drug‐delivery systems. Zhang et al. demonstrated the enhanced solubility and efficacy of KAE through complexation, while Zhou et al. showed the potential of TDNs for targeted drug delivery in treating corneal injuries. Yin et al. improved drug retention and release with therapeutic contact lenses, and Cao et al. introduced pH‐responsive polymers for effective siRNA delivery. Future research should focus on optimizing these nanocomplex systems for broader applications, exploring their potential in treating a range of ocular conditions and further improving their stability and efficacy for clinical use.

### 3.10 Nanofibers

Nanofibers are ultrafine fibers with diameters typically ranging from 1 to 1000 nm. Their unique properties include a high aspect ratio, a large surface area to volume ratio, and distinct mechanical, optical, and electrical characteristics.^[^ ^243^ ^]^ These features make nanofibers highly versatile, enabling their application across various fields, such as tissue engineering, drug delivery, filtration, and energy storage.^[^ ^244^ ^]^ The materials used for nanofiber production include electrospinning, self‐assembly, with electrospinning being the most commonly used method due to its simplicity, flexibility, and scalability.^[^ ^245^ ^]^ These properties position nanofibers as promising candidates for biomedical applications, particularly in ocular therapy, where their structural and functional benefits can aid in the treatment of eye injuries and diseases.

Cejkova et al. investigated the use of nanofiber scaffolds for delivering MSCs to treat corneal alkali burns.^[^ ^116^ ^]^ Their study showed that nanofiber scaffolds improved MSC retention and viability at the injury site, enhancing therapeutic outcomes. MSCs delivered via these scaffolds effectively reduced oxidative stress, inflammation, and neovascularization, leading to faster corneal healing. This approach addresses the limitations of using MSCs alone, such as low survival rates, and demonstrates the potential of nanofiber scaffolds for treating corneal chemical injuries.^[^ ^116^ ^]^

**Figure 23:**
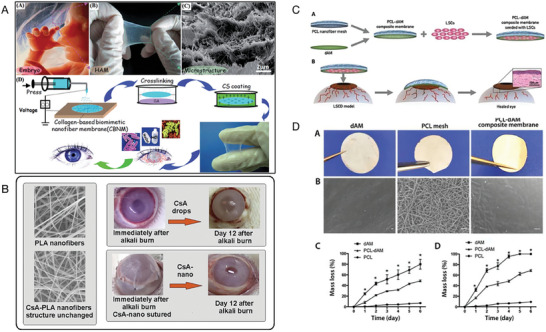
Three studies on drug‐delivery systems using nanofibers to enhance treatment of ocular surface chemical burns. A) Procedure for fabricating CS‐based nanofibrous membranes (CBNMs) that replicate the microstructure and architecture of devitalized tissue membranes, such as human amniotic membrane (HAM), through electrospinning and CS modification for corneal damage treatment: A–C) Base‐side macroscopic morphology and microstructure of HAM, consisting of randomly arranged nanoscale collagen fibers; D) Procedure for CBNM fabrication. Reproduced with permission.^[^ ^117^ ^]^ Copyright 2014, Royal Society of Chemistry. B) CsA‐loaded electrospun nanofibers were applied to alkali‐injured ocular surfaces, presenting a novel method for reducing inflammation and neovascularization in rabbit corneas damaged by alkali burns. Reproduced with permission.^[^ ^119^ ^]^ Copyright 2016, Elsevier. C) A composite membrane was fabricated by incorporating an electrospun mesh of poly(ε‐caprolactone) (PCL) nanofibers to strengthen the decellularized amniotic membrane (dAM) sheet via covalent bonding at the interface. This method aimed to maintain the distinctive bioactivity of dAM. In an experimental rabbit model of limbal stem cell (LSC) deficiency caused by an alkaline burn, the PCL‐dAM composite membrane exhibited enhanced characteristics in repairing corneal damage. It facilitated the transplantation of LSCs, promoted re‐epithelialization, and mitigated inflammation and neovascularization. D) Overall look and deterioration patterns of the PCL‐dAM composite membrane: A) Visual characteristics and B) Scanning electron microscopy images of dAM, PCL, and the PCL‐dAM composite membranes created using the PCL fiber lattice and dAM; C,D) Disintegration profiles of the three specimens in PBS and when exposed to an enzyme (1 U of pancreatin), respectively. *p < 0.001 compared with dAM or PCL lattice. Reproduced with permission.^[^ ^120^ ^]^ Copyright 2019, Elsevier.

Continuing the innovation in nanofiber technology, Ye et al. developed biomimetic nanofibrous membranes using collagen and CS to enhance wound healing for chemically damaged corneas.^[^ ^117^ ^]^ The combination of collagen and hyaluronate provides structural support, while CS improves mechanical properties and promotes selective cell adhesion. The nanofiber membranes, produced through electrospinning and surface modification, are designed to adhere specifically to epithelial cells and fibroblasts. Compared to traditional dressings, these CS‐modified collagen‐based biomimetic nanofibrous membranes offer better transparency, mechanical strength, and biological effectiveness, making them a promising option for treating corneal injuries^[^ ^117^ ^]^ (**Figure** **23**A).

Using a needleless electrospinning technique, nanofiber scaffolds were fabricated from the biocompatible polymer poly(L‐lactic) acid (PLA).^[^ ^246^ ^]^ Cejkova and colleagues employ these nanofiber scaffolds as a delivery platform for bone‐marrow‐derived MSCs and tissue‐specific LSCs. In this research, the therapeutic efficacy of MSCs was contrasted with that of LSCs in a rabbit model of ocular injury, revealing similar efficacy in enhancing the regeneration of the cornea.^[^ ^118^ ^]^ Incorporating nanofiber scaffolds improved the retention and continuous release of MSCs and LSCs at the injury location, fostering their therapeutic impact by facilitating cellular engraftment, diminishing inflammation, and bolstering corneal regeneration. Therefore, MSCs derived from bone marrow exhibit therapeutic effects comparable to tissue‐specific LSCs in promoting corneal healing, suggesting their potential as an alternative source of stem cells for ocular surface regeneration when tissue‐specific LSCs are unavailable or difficult to obtain.^[^ ^118^ ^]^

Further enhancing nanofiber applications, Cejkova et al. explored the use of CsA‐loaded nanofibers to reduce inflammation and inhibit neovascularization in alkali‐injured rabbit corneas.^[^ ^119^ ^]^ CsA, known for its anti‐inflammatory properties, was encapsulated in PLA nanofibers to enhance bioavailability and provide sustained release (Figure 23B). The approach improved CsA's retention on the ocular surface, reducing the need for frequent dosing. The nanofibers effectively suppressed inflammation, minimized neovascularization, and promoted corneal healing, showing potential as a novel treatment for chemical burns on the cornea.^[^ ^119^ ^]^

Zhou et al. investigated a nanofiber‐enhanced dAM to enhance limbal stem cell transplantation for treating corneal epithelial lesions in rabbits (Figure 23C).^[^ ^120^ ^]^ The composite consisted of dAM, known for its regenerative and anti‐inflammatory properties, strengthened by electrospun bioabsorbable PCL nanofibers to improve mechanical durability (Figure 23D). The addition of the PCL nanofibers addressed the limitations of dAM alone, such as weak mechanical strength and rapid degradation.^[^ ^120^ ^]^ This enhanced membrane showed improved stability, supporting LSC transplantation, reducing inflammation, and promoting corneal healing.^[^ ^120^ ^]^

Chen and colleagues developed a biocompatible nanofiber incorporating a VEGF peptide to inhibit CNV.^[^ ^121^ ^]^ This nanofiber is composed of a NapFF peptide conjugated to the bioactive HRH peptide, which binds to VEGFR to prevent abnormal vascular growth.^[^ ^121^ ^]^ Using the HRH peptide alone poses challenges due to instability and low bioactivity, necessitating high doses.^[^ ^121^ ^]^ By applying self‐assembling peptide amphiphile nanotechnology, the nanofibers enhanced the peptide's bioactivity and stability, improving anti‐angiogenic effects. The approach shows promise for treating ocular vascular diseases with enhanced therapeutic efficacy.^[^ ^121^ ^]^

In a novel approach, Razavi and colleagues developed a dual‐drug transporter using core‐shell nanofibers for treating alkali burns in the cornea, incorporating curcumin in the core and vancomycin in the shell.^[^ ^122^ ^]^ Curcumin is known for its anti‐inflammatory and anti‐angiogenic properties, and vancomycin has strong bactericidal effects, whereas their individual efficacy is limited.^[^ ^247^ ^]^ The researchers employed coaxial electrospinning to create the nanofibers, with silk fibroin loaded with curcumin in the core and a CS‐polyvinyl alcohol (PVA) mixture containing vancomycin in the shell. The design aimed to enhance drug release duration and bioavailability.^[^ ^122^ ^]^ The integrated nanomaterials promoted corneal re‐epithelialization, reduced inflammation, and prevented CNV. Overall, the study highlights the potential of this dual‐drug transporter as an effective treatment strategy for corneal alkali burns.^[^ ^122^ ^]^

Nanofibers are proving to be a versatile and effective tool in the treatment of ocular surface chemical injuries. Their ability to enhance drug delivery, support stem cell therapies, and improve wound healing underscores their potential in advancing ocular surface treatments. Future research should focus on optimizing these nanofiber‐based strategies, exploring their long‐term effects, and evaluating their clinical applicability. Innovations in nanofiber technology could lead to more effective, personalized treatments for ocular surface chemical injuries, ultimately improving patient outcomes and recovery.

### 3.11 Nanozymes

Nanozymes, which are nanomaterials exhibiting enzyme‐like catalytic properties, can transform enzyme substrates into various products.^[^ ^248^ ^]^ Compared to natural enzymes, they offer several advantages including their simplicity and cost‐effectiveness of synthesis, resilience in harsh environments, and the ability for direct surface modification alongside multifunctional catalytic capabilities.^[^ ^248^, ^249^ ^]^ Given these characteristics, nanozymes become particularly valuable in the field of nanomedicine, especially for addressing ailments associated with oxidative stress and inflammation, such as ocular disorders. They can effectively cross ocular barriers, enabling targeted and sustained therapy. ^[^ ^248^ ^]^ Nanozymes have shown potential in improving drug delivery, therapeutic efficacy, and disease management, often in combination with soft biomaterials like contact lenses and microneedles.^[^ ^248^ ^]^

**Figure 24:**
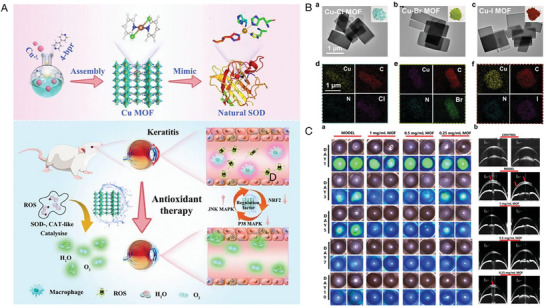
An example metal–organic framework (MOF) nanozyme as a drug‐delivery system to treat ocular surface chemical burns. A) Schematic illustration depicting the synthesis process of copper‐based MOF nanozymes that emulate superoxide dismutase for treating chemical burns on the cornea. B) Analysis of Cu‐X MOFs: a–c) TEM images of the Cu‐X MOFs. The insets include photographs of the powder samples; d–f) Corresponding EDS mapping data. C In the alkali burn animal model, Cu–Cl MOF was found to mitigate corneal damage: a) Images obtained from broad‐beam lighting and fluorescein staining of the ocular surface treated with varying concentrations of Cu–Cl MOF on days 1, 3, 5, 7, and 10 following the alkali burn; b) Representative AS‐OCT images illustrating the anterior segment of the ocular surface, with the red arrow highlighting defects or irregularities in the corneal epithelium. Reproduced with permission.^[^ ^123^ ^]^ Copyright 2023, Springer.

Tang et al. investigated halogen‐coordinated copper‐based metal‐organic frameworks (Cu‐X MOFs) as biomimetic catalysts for treating chemical corneal burns by modulating their enzymatic activity through precise coordination of halogen atoms.^[^ ^123^ ^]^ The primary components are copper nodes coordinated with halogens (Cu‐X), specifically Cu–Cl, Cu–Br, and Cu–I, with Cu–Cl showing the highest efficacy. These structures mimic superoxide dismutase (SOD), which converts harmful ROS into harmless compounds. However, Cu–Cl alone faces challenges such as instability and limited effectiveness in biological systems without a proper delivery mechanism. Their study utilized nanotechnology to integrate Cu‐X MOFs as nanozymes, which are nano‐sized enzyme mimics. The MOFs were synthesized using copper sulfate pentahydrate (CuSO4·5H_2_O), sodium X (X = Cl, Br, or I), and 4,4′‐bipyridine.^[^ ^250^ ^]^ These nanozymes exhibit enhanced enzyme‐like behavior, biological compatibility, and the ability to scavenge ROS, thereby reducing oxidative stress and preventing cell death and inflammation in corneal epithelial cells. The incorporation of Cu‐X MOFs significantly improved the stability, bioavailability, and catalytic efficiency of copper nodes, suggesting a novel therapeutic strategy for addressing oxidative stress‐related ocular diseases (**Figure** **24**).

### 3.12 Nano‐Composite Materials

To enhance corneal epithelial cell transplantation and wound healing, Xu et al. explored the development of blended membranes composed of CMCTS, gelatin, and HA.^[^ ^124^ ^]^ The introduction of gelatin or CS can lead to excessive swelling, which diminishes mechanical stability and effectiveness in tissue regeneration without proper cross‐linking or blending.^[^ ^124^ ^]^ CMCTS serves as the primary active ingredient, promoting corneal epithelial cell attachment and proliferation while facilitating wound healing. The combination of CMCTS, gelatin, and HA results in a transparent, biodegradable, and biocompatible membrane that supports corneal epithelial cell growth and enhances corneal wound healing and epithelial reconstruction. The findings demonstrated that these blended membranes effectively promote the proliferation of corneal epithelial cells and significantly improve recovery and regeneration of corneal injuries in rabbits following alkali burns.^[^ ^124^ ^]^

**Figure 25:**
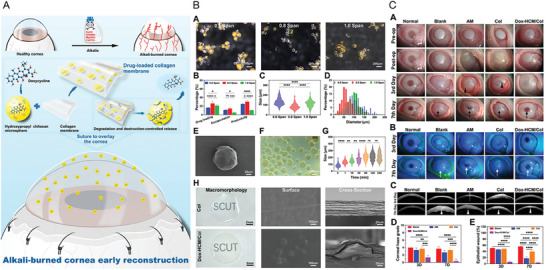
Nano‐composite materials for drug delivery in treating ocular surface chemical burns. A) Schematic of Dox‐HCM/Col, where Dox‐HCMs are incorporated into collagen (Col) to regulate doxycycline (Dox) release and facilitate the early reconstruction of alkali‐burned corneas. B) Optimization and initial characterization of Dox‐HCM and Dox‐HCM/Col: A) Different preparation conditions' impact on the macromorphology of Dox‐HCM; B) Variation in drug loading, encapsulation efficiency, and productivity among different Dox‐HCM formulations; C) Particle size analysis of various Dox‐HCM formulations; D) Size distribution of microspheres with varying Span concentrations; E) Microscopic features of Dox‐HCM prepared using Span 0.8; F) Macromorphology of Dox‐HCM under Span 0.8 in DPBS at 37 °C for 2 h; G) Swelling behavior over time and size distribution analysis; H) Macroscopic and microscopic observations of Col and Dox‐HCM/Col, including the influence of Span 80. C) Transplantation of Dox‐HCM/Col onto rabbit corneas with alkali burns to assess tissue reconstruction and postoperative outcomes: A) Pre‐operative slit lamp microscopic images demonstrating the extent of corneal injury (indicated by a black dashed circle), and post‐operative photographs confirming the therapeutic potential of Dox‐HCM/Col; B) Corresponding corneal fluorescein staining images; C Representative AS‐OCT examination images; D) Corneal haze grade bar graph; E) Quantification of corneal epithelial wounds. Reproduced with permission.^[^ ^125^ ^]^ Copyright 2023, Elsevier.

In a related development, Liu et al. developed a drug‐loaded collagen membrane to treat alkali‐induced corneal burns by fostering an optimal environment for epithelial cell growth and enabling controlled drug release, thereby accelerating corneal regeneration.^[^ ^125^ ^]^ The active ingredient, doxycycline (Dox), selectively inhibits MMPs, which play a crucial role in tissue degradation and remodeling.^[^ ^251^ ^]^ However, the use of Dox for treating corneal burns faces challenges due to its limited bioavailability and rapid loss when administered systemically or as eye drops.^[^ ^237^ ^]^ Therefore, the formulation combined Dox with hydroxypropyl CS microspheres (HCM) and collagen membrane (Col), effectively inhibiting MMP‐9 and ‐13, promoting corneal re‐epithelialization, and ensuring sustained drug release. Thus, the incorporation of nanomaterials allowed for prolonged therapeutic effects and enhanced drug availability on the corneal surface. The Dox‐HCM/Col membrane effectively inhibited MMP activity, promoted corneal healing, and accelerated reconstruction, presenting a promising therapy for corneal alkali burns (**Figure** **25**).^[^ ^125^ ^]^

The advancements in nano‐composite materials for the treatment of ocular surface chemical injuries highlight their potential in enhancing therapeutic outcomes. The development of multifunctional membranes and drug‐delivery systems, as exemplified by the studies on blended membranes and drug‐loaded collagen matrices, offers significant improvements in corneal wound healing and regeneration. Future research should focus on optimizing the mechanical properties, drug release profiles, and biocompatibility of these materials. Additionally, exploring the integration of drug‐delivery systems with emerging technologies, such as personalized medicine and real‐time monitoring, could further enhance treatment efficacy and improve patient outcomes in the management of ocular surface injuries.

## 4 Advantages of Nanotechnology for Treating Ocular Diseases

### 4.1 Targeted Delivery

Nanotechnology offers notable advantages in targeted drug delivery, particularly in ocular surface chemical injuries. Unlike traditional drug therapies, nanomaterials can localize precisely at lesion sites through surface modifications, enhancing treatment efficacy. These targeted delivery capabilities make nanomaterials highly beneficial for treating ocular diseases.

#### 4.1.1 Localized Drug Delivery

Nanomaterials enable precise delivery of therapeutic agents directly to damaged ocular surface tissues, ensuring higher drug concentrations at the treatment site and enhancing efficacy. This localized delivery, facilitated by nanotechnology, allows for treatments that were previously systemic to be administered directly to the eye, reducing side effects and enhancing therapeutic outcomes.

#### 4.1.2 Prolonged Drug Retention

Nanotechnology can enhance drug retention on the ocular surface through surface modifications and drug encapsulation, thereby providing prolonged treatment efficacy. For instance, lipid vesicles adhere to the cornea and effectively deliver poorly soluble drugs.^[^ ^128^ ^]^ Unlike traditional eye drops, which are quickly washed away, nanotechnology extends drug retention times, reducing dosing frequency, maintaining consistent drug levels, and improving patient compliance.

#### 4.1.3 Targeting Damaged Tissues

Functionalized surfaces and selective drug carriers enable nanomaterials to target damaged ocular tissues specifically. Surface‐modified NPs can bind to damaged cells through specific receptors, increasing local drug concentrations while reducing impact on healthy tissues. For instance, positively charged lipid vesicles adhere to the negatively charged mucin layer on the corneal epithelium, enhancing drug absorption.^[^ ^19^ ^]^

#### 4.1.4 Reduced Systemic Side Effects

Nanomaterials minimize systemic drug distribution, which lowers the risk of systemic side effects. This is particularly important for ocular surface injuries as it focuses the therapeutic effect on the damaged area while minimizing adverse effects elsewhere in the body.

In summary, introducing nanomaterials to treat ocular surface chemical injuries can enhance local drug concentration and efficacy, reduce systemic side effects, and selectively target damaged tissues through specialized design and functionalization.

### 4.2 Biocompatibility

Nanomaterials like biodegradable polymer NPs, liposomes, and hydrogels are highly compatible with biological systems due to their ability to break down into harmless byproducts. Their biocompatibility is enhanced by optimizing their composition and structure to reduce immune responses and inflammation. In treating ocular surface chemical injuries, this biocompatibility is evident in several key aspects.

#### 4.2.1 Non‐Toxicity and Non‐Irritation

Nanomaterials used should not induce toxicity or irritation to ocular tissues. Biocompatible nanomaterials are designed to be non‐toxic and non‐irritating to ensure they do not exacerbate the injury or cause adverse reactions. Local application also allows for treatment with lower concentrations of the drug to treat ocular surface diseases.

#### 4.2.2 Biodegradability

Biodegradable nanomaterials break down into non‐toxic byproducts after performing their function. This characteristic reduces the risk of long‐term accumulation and potential harm to the eye.

#### 4.2.3 Minimal Immune Response

Biocompatible nanomaterials are engineered to minimize immune responses or inflammatory reactions upon administration to the ocular surface, ensuring that the treatment does not trigger unnecessary immune reactions that could impair healing or cause discomfort.

#### 4.2.4 Compatibility with Ocular Physiology

The physical and chemical properties of nanomaterials are tailored to match the physiological environment of the eye. This includes factors such as pH sensitivity, osmolarity, and interaction with ocular fluids to maintain stability and effectiveness during treatment.

#### 4.2.5 Long‐Term Safety

Biocompatible nanomaterials are assessed for their long‐term safety profile, ensuring that they do not pose risks such as chronic inflammation, fibrosis, or tissue damage over extended periods of use.

In summary, biocompatibility in the context of nanomaterials for treating ocular surface chemical injuries ensures their safety and compatibility with ocular tissues, minimizing adverse effects while promoting effective therapeutic outcomes.

### 4.3 Enhanced Healing

Nanotechnology can enhance tissue healing through precise drug delivery and may further directly support repair processes. Nanofiber scaffolds with embedded growth factors offer structural support and encourage the proliferation and migration of corneal epithelial cells, accelerating wound closure. Additionally, NPs can deliver antioxidants to combat oxidative stress and protect ocular tissues. The benefits of enhanced healing with nanomaterials when treating ocular surface chemical injuries are evident in several key areas.

#### 4.3.1 Promotion of Cell Proliferation and Migration

Nanomaterials can be engineered to encourage the proliferation and migration of corneal epithelial cells or other cells on the ocular surface. This accelerates the regeneration of damaged tissues and promotes faster healing.

#### 4.3.2 Support for Tissue Regeneration

Nanofiber scaffolds and hydrogels are examples of nanomaterials that offer physical support for tissue regeneration; they create an environment conducive to the growth and organization of new cells, aiding in the repair of injured ocular tissues.

#### 4.3.3 Delivery of Growth Factors and Bioactive Molecules

Nanomaterials can act as carriers for growth factors, cytokines, or other bioactive molecules essential for tissue repair and regeneration. By delivering these molecules directly to the injury site, nanomaterials enhance the biological processes involved in healing.

#### 4.3.4 Reduction of Oxidative Stress

Some nanomaterials have antioxidant properties or can deliver antioxidants to the damaged ocular surface. By mitigating oxidative stress, these materials protect cells from further damage and create a more favorable healing environment.

#### 4.3.5 Improved Wound Closure and Barrier Function

Nanomaterials can facilitate faster wound closure and enhance the barrier function of the ocular surface. This decreases the risk of infection and reduces exposure to external irritants, thus supporting the overall healing process.

#### 4.3.6 Anti‐Inflammatory and Anti‐Angiogenic Effects

Certain nanomaterials exhibit anti‐inflammatory properties and can inhibit neovascularization. These features help reduce inflammation on the ocular surface and prevent abnormal blood vessel formation, promoting a smoother healing process.

#### 4.3.7 Anti‐Ferroptosis Mechanism

Nanomaterials loaded with drugs can also promote cell survival by inhibiting ferroptosis, an iron‐dependent form of cell death. Ferroptosis plays a significant role in oxidative stress and cell damage. By targeting ferroptosis, nanomaterials can further protect ocular cells and enhance healing effectiveness.

In conclusion, nanomaterials improve the treatment of ocular surface chemical injuries by accelerating healing through various mechanisms. They enhance cell proliferation and migration, support tissue regeneration, deliver bioactive molecules, reduce oxidative stress, and improve wound closure and barrier function. Additionally, they provide anti‐inflammatory and anti‐angiogenic effects and inhibit ferroptosis, leading to the faster and more effective recovery of the ocular surface.

## 5 Current Research Challenges and Limitations of Nanotechnology to Treat Ocular Injuries

### 5.1 Long‐Term Safety

Encouraging initial results motivate researchers to further investigate the long‐term safety of nanotechnology in treating ocular injuries. Important concerns include the metabolic pathways of nanomaterials in the eye, the potential toxicity with prolonged exposure, and the impact on the immune systems. Long‐duration animal studies and clinical trials are essential for evaluating the safety profile of nanomaterials over extended periods to treat ocular surface diseases.

### 5.2 Cost of Production

The complex and costly nature of nanomaterial manufacturing hinders their broad clinical application. Developing efficient, cost‐effective synthesis methods and scalable production techniques is crucial. Reducing the cost of production is a key step toward the widespread clinical adoption of nanotechnology.

### 5.3 Quality Control and Standardization

The biological effects of nanomaterials when treating ocular surface injuries are significantly influenced by their physical and chemical properties, such as particle size, shape, and surface characteristics. Ensuring the safety and efficacy of nanomaterials necessitates strict quality control and standardized production processes. Presently, the lack of uniform standards and regulations is a major challenge. Future efforts should aim at creating universally accepted standards and testing procedures to ensure the consistency and reliability of nanomaterials.

## 6 Future Prospects

### 6.1 Expanded Applications

The efficient application of nanotechnology for treating chemical injuries to the ocular surface provides valuable insights into its potential use in addressing various other eye ailments. For instance, nanomaterials could potentially tackle challenging conditions such as glaucoma and macular degeneration by employing precise methods of drug delivery and gene therapy, thereby introducing novel approaches to treatment. The adaptability and efficacy of nanotechnology indicate numerous opportunities for integration into the management of eye diseases.

### 6.2 Multidisciplinary Collaboration

Collaborative efforts across different fields such as pharmacology, materials science, ophthalmology, and bioengineering are crucial for advancing nanotechnology. By promoting interdisciplinary collaboration, we can accelerate technological progress, facilitating the transition of nanomaterials from experimental labs to clinical applications. Future initiatives need to focus on enhancing cooperation among various disciplines to promote the use of nanotechnology in medical contexts.

### 6.3 Development of New Materials and Technologies

The emphasis in future research should be on developing novel nanomaterials and technologies to enhance therapeutic results. For example, advanced and new nanomaterials like nanozyme and nanocages were introduced to treat ocular surface chemical burns. Other advanced nanomaterials with the ability to release medications in response to environmental signals (such as alterations in pH and temperature) offer tailored and accurate therapies. Furthermore, nanocarriers integrating gene‐editing techniques offer potential for gene therapy use in ophthalmic treatments. Currently, nanotechnology in the treatment of ocular surface diseases is primarily focused on drug delivery. Utilizing nanomaterials and nanotechnology to produce corneal substitute materials may be a significant direction for future development.

## 7 Conclusions

Ocular surface chemical injuries present considerable challenges, frequently leading to long‐term visual damage and extended healing times. Among these, alkali burns to the cornea are especially difficult to treat. Current treatments for corneal chemical burns typically involve single‐action drugs with a wide range of options, frequent administration schedules, potential side effects, and limited effectiveness. Nanomedicine and the delivery of drugs at the nanoscale herald a new era in treatment strategies; they aim to revolutionize methods by enhancing safety, compatibility with biological systems, drug availability, therapeutic outcomes, and patient adherence. Simultaneously, they seek to reduce dosage requirements, rates of elimination, and the complexity of metabolic processes. This innovative approach represents a promising avenue for developing next‐generation therapies tailored to address the complexities of ocular surface chemical injuries.^[^ ^252^ ^]^

Recent advancements have witnessed the development of various nanocarrier systems, including liposomes, nanoemulsions, nanomicelles, NWs, NLCs, NPs, hydrogels, dendrimers, nanocomplexes, nanofibers, nanozymes, and nano‐composite materials, aimed at addressing the limitations of conventional drugs for treating ocular surface chemical burns. Nanotechnology has demonstrated considerable potential in treating ocular surface chemical injuries, complementing conventional treatment approaches by leveraging advantages such as targeted delivery, biocompatibility, and healing promotion. This review has explored the utilization and advancements of nanomedicine in treating ocular surface chemical burns, emphasizing nano‐formulations that include both traditional medications and natural compounds.

As the field of conventional and nanomedicine drug‐delivery systems continues to evolve, ongoing research endeavors are anticipated to yield promising outcomes, further enhancing treatment options for ocular surface chemical burns. However, challenges including long‐term safety, production costs, and quality control must be addressed. Looking ahead, through interdisciplinary collaboration and technological innovation, nanotechnology is poised to assume a pivotal role in addressing ophthalmic diseases. By studying the development of nanotechnology in treating ocular surface chemical injuries, we have demonstrated significant progress in treating ophthalmic diseases, serving as a microcosm of advancements in the field.

## Conflict of Interest

The authors declare no conflict of interest.
